# Endothelial Lon protease 1 facilitates the redox balance to prevent glomerulosclerosis by acting on superoxide dismutase 2 ubiquitination

**DOI:** 10.1016/j.redox.2025.103929

**Published:** 2025-11-19

**Authors:** Xiaolu Zhang, Shuzhen Li, Shanshan Li, Bing Liu, Guixia Ding, Mengqiu Wu, Yue Zhang, Songming Huang, Wei Gong, Zhanjun Jia, Aihua Zhang

**Affiliations:** a Department of Nephrology, Children's Hospital of Nanjing Medical University, Nanjing, 210008, China; b Nanjing Key Lab of Pediatrics, Children's Hospital of Nanjing Medical University, Nanjing, 210008, China; c Jiangsu Key Laboratory of Early Development and Chronic Diseases Prevention in Children, Nanjing Medical University, Nanjing, 210029, China; d State Key Laboratory of Reproductive Medicine and Offspring Health, Nanjing Medical University, Nanjing, 211166, China; e Department of Child Health Care, Children's Hospital of Nanjing Medical University, Nanjing, 210008, China

**Keywords:** LONP1, SOD2, Endothelial cell, Redox balance, Glomerulosclerosis

## Abstract

Endothelial injury is an early event in chronic kidney disease (CKD) leading to renal hemodynamic disorders and even glomerulosclerosis. During this process, both oxidative stress and inflammation originating from injured endothelial cells can initiate pathogenic cell-to-cell interactions via a paracrine mechanism. Accumulating evidence underscores the pivotal role of mitochondrial dysfunction as a crucial mechanism underlying endothelial dysfunction. Lon protease 1 (LONP1) is a mitochondrial protease that plays a key role in maintaining mitochondrial homeostasis; however, its role in endothelial dysfunction-related renal disease is unknown. In CKD patients and mice subjected to 5/6 nephrectomy (5/6Nx), we observed decreased LONP1 expression in glomerular endothelial cells. Interestingly, endothelial cell-specific heterozygous knockout of LONP1 exacerbated glomerulosclerosis and aggravated renal function decline, proteinuria, hypertension and kidney inflammation in 5/6Nx mice. Mechanistically, our results suggest that the loss of LONP1 strikingly increased reactive oxygen species (ROS) levels by promoting the ubiquitination of mitochondrial superoxide dismutase 2 (SOD2); which in turn led to mitochondrial dysfunction and inflammation within endothelial cells. Additionally, the increase in mitochondrial ROS and subsequent production of inflammatory cytokines from damaged endothelial cells further trigger mesangial cell proliferation and podocyte injury, which together result in glomerulosclerosis and CKD progression. Taken together, our findings identify LONP1 as a therapeutic target for balancing glomerular redox, alleviating inflammation, and retarding glomerulosclerosis.

## Highlights

• LONP1 is deficient in the glomerular endothelial cells, and plays a protective role in glomerulosclerosis progression.
• LONP1 acts as a novel binding protein for SOD2 to stabilize its expression by protecting against SOD2 ubiquitination.
• LONP1 deficiency causes redox imbalance, mitochondrial dysfunction, and cellular inflammatory injury.
• LONP1 deficiency in endothelial cells exerts paracrine effects on mesangial cells and podocytes.
• Endothelial cell-specific heterozygous knockout of LONP1 exacerbates glomerulosclerosis and CKD progression.

## Graphical abstract

**Figure:**
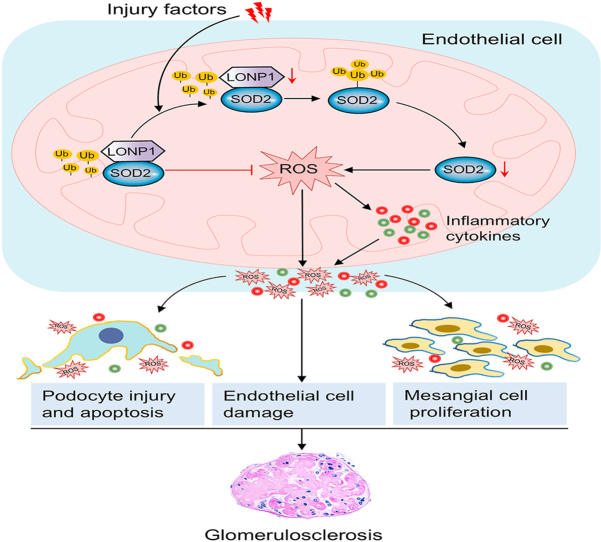

## 1 Introduction

Chronic kidney disease (CKD), a long-term irreversible renal injury with multiple aetiologies, has become a global public health problem characterized by a high morbidity rate, high medical costs, and increasing mortality [[1], [2], [3]]. According to epidemiological statistics, approximately 700 million patients have been diagnosed with CKD in the world, about one-fifth of which are in China [4,5]. Therefore, it is necessary to identify early intervention targets for preventing and treating CKD. Due to the highly vascularized nature of the kidney, disruption of vascular endothelial cell homeostasis plays a promoting role in renal fibrosis and CKD [[6], [7], [8], [9]]. And the damaged glomerular endothelial cells are the vital initiator of glomerulosclerosis characterized by glomerular capillary loop collapse, mesangial cell proliferation, and inflammatory cell infiltration. Among the various causes of endothelial cell damage, mitochondrial homeostasis imbalance has received increasing attention in recent years. Imbalanced mitochondrial homeostasis triggers a series of oxidative stress and inflammatory responses, thereby disrupting the vascular endothelial cell barrier and participating in the pathological process of CKD renal fibrosis [[10], [11], [12], [13]]. Therefore, it is important to investigate the regulatory mechanisms of mitochondrial homeostasis in vascular endothelial cells during the CKD process.

Lon protease 1 (LONP1) is a key protease that maintains mitochondrial homeostasis, primarily responsible for degrading misfolded and oxidized modified proteins, functioning as a molecular chaperone, and binding to mitochondrial DNA (mtDNA) [14,15]. LONP1 is widely expressed in various tissues and organs and is closely associated with various diseases. Due to the high conservation in the evolution, LONP1 gene mutations lead to serious genetic diseases, such as a rare autosomal dominant syndrome Cerebro-Oculo-Dento-Auditory Syndrome (CODAS) that is characterized by abnormalities in the brain, eyes, teeth, ears, and bones [16]. Previous studies have shown that mouse embryos with systemic homozygous loss of LONP1 are lethal, suggesting its important role in the growth and development of mouse embryos [17,18]. In addition, while LONP1 expression in cardiac mitochondria increases with age, its protease activity gradually decreases [19]. In the early stage of lung fibroblast aging induced by H_2_O_2_, LONP1 expression is obviously increased to degrade abnormal oxidative proteins, but LONP1 expression decreases over time, causing the accumulation of oxidized proteins and leading to cellular aging [20]. These previous results suggest that a decrease in LONP1 expression or activity might be a direct pathogeny of cellular aging. Another study has demonstrated that overexpression of LONP1 in wild-type mice protects the heart from ischemia–reperfusion (I/R) injury, whereas the heterozygous knockout of LONP1 eliminates its protective effect on the myocardium, indicating that LONP1 is an endogenous cardioprotective factor [21]. Additionally, it has shown that disuse muscle atrophy is associated with decreased mitochondrial LONP1 levels in both humans and mice, and the specific knockout of LONP1 in mouse skeletal muscle leads to the abnormal protein degradation disorders in muscle mitochondria and activates autophagy and lysosomal degradation programs, resulting in muscle fibers thinning [22]. Our previous studies revealed that conditional knockout of LONP1 in mouse kidney podocytes resulted in mitochondrial dysfunction, leading to podocytopathy and glomerular injury [23]. Moreover, LONP1 targets HMGCS2 in renal tubular epithelial cells has been found to protect mitochondrial function and inhibit tubulointerstitial fibrosis [24]. However, given the importance of the endothelial barrier and differences in protein function, the role of endothelial LONP1 in CKD has not been fully studied.

Mitochondria are the main sites of reactive oxygen species (ROS) production, and abnormal ROS accumulation causes oxidative damage to cellular proteins, lipids and nucleic acids, leading to structural and functional damage [25,26]. LONP1 is a key enzyme that resists mitochondrial oxidative stress and maintains the redox balance [27,28]. However, no relevant reports on the pathway through which LONP1 inhibits ROS production, fights oxidative stress-induced damage, protects vascular endothelial cell mitochondrial homeostasis, and thus plays a protective role in CKD are available. Antioxidant enzymes are important active oxygen-scavenging systems in organisms [29,30]. They are a collective term for superoxide dismutase (SOD), thioredoxin peroxidase (TPX), glutathione peroxidase (GSH), and catalase (CAT), among which SOD is the primary protein that clears free radicals and widely exists in animals, plants, and microorganisms [31,32]. SOD is divided into SOD1, SOD2, and SOD3 according to the metal cofactors, and SOD2 is the only one SOD that is exclusively located in mitochondria [33,34]. Studies have shown that SOD2 plays an important role in preventing oxidative stress-related diseases, such as inflammation, cancer, cardiovascular diseases, and kidney disease [[35], [36], [37], [38], [39]]. In this study, we observed a striking increase in mitochondrial ROS levels in endothelial cells with LONP1 silencing. Moreover, bioinformatic analysis of LONP1 and key mitochondrial antioxidant proteins, revealed potential binding sites between LONP1 and SOD2. Therefore, we hypothesize that an interaction between LONP1 and SOD2 may play an important role in the pathogenesis of glomerulosclerosis.

In this study, we observed the downregulation of LONP1 in endothelial cells of fibrotic kidney tissues from CKD patients and 5/6Nx model mice. The decreased expression of LONP1 in endothelial cells can lead to redox imbalance by protecting against SOD2 ubiquitination. The redox imbalance causes mitochondrial dysfunction and inflammatory damage of endothelial cells, and damaged endothelial cells indirectly injury the mesangial cells and podocytes, together aggravates the process of glomerulosclerosis.

## 2 Material and methods

### 2.1 Patients and study approval

Renal biopsy samples were collected from patients diagnosed with CKD in the Department of Nephrology, and nontumor renal tissues were from patients with renal cell carcinoma in the Department of Urology of Children's Hospital of Nanjing Medical University. The clinical features of CKD patients and pathologic diagnosis of renal biopsy are shown in Table S1. The collection of human tissues was conducted in accordance with the reporting guideline of the Biospecimen Reporting for Improved Study Quality (BRISQ) [40]. All experiments involving human tissue obtained approval from the Institutional Review Committee of Children's Hospital of Nanjing Medical University (project number: 201902041-1).

### 2.2 Animals

C57BL/6J wild-type male mice (approximately 8–10 weeks and weighing 25–30g) were purchased by GemPharmatech Co., Ltd (Nanjing, China). Endothelial cell-specific LONP1 knockout mice (LONP1^flox/+^; Tek-Cre, short for hetero cKO) were generated from LONP1^flox/flox^ mice (obtained from Professor Lu Bin, Wen Zhou Medical University, China) and C57BL/6J strain Tek-Cre mice (purchased from the Jackson Laboratory, Bar Harbor, Maine, USA). Mice were maintained in the temperature-controlled (19–21 °C) standardized SPF level experimental animal barrier facility with a 12-h light‒dark cycle at the Experimental Animal Center of Nanjing Medical University. Mice were fed a standard rodent diet and allowed access to drinking water freely. All animal experiments were conducted in accordance with the ARRIVE 2.0 reporting guidelines [41] approved by the Ethics Committee for the Use of Laboratory Animals at Nanjing Medical University (project number: 2310085).

### 2.3 Five-six nephrectomy (5/6Nx) model

Male LONP1^flox/flox^ and LONP1^flox/+^; Tek-Cre mice aged 8–10 weeks, weighing approximately 25–30 g, were randomly divided into four groups (WT group, WT+5/6Nx group, hetero cKO group, and hetero cKO+5/6Nx group). The 5/6Nx surgery was performed on model group as described previously [42]. Briefly, the mice were anaesthetized with isoflurane gas, the left kidney was separated via a left flank incision, the upper and lower poles were resected, and the entire right kidney was removed through a second surgery via a right flank incision after 1 week [42]. After 4 weeks, the blood pressure of the mice was measured with a tail-cuff system (BP-2000; Visitech Systems, Apex, NC). Twenty-four-hour urine samples were collected to detect urinary protein levels, and blood urea nitrogen (BUN) and serum creatinine (Cr) levels were measured on an automatic biochemical analyzer.

### 2.4 MnTBAP treatment

Male LONP1^flox/+^; Tek-Cre mice aged 8–10 weeks, weighing approximately 25–30 g, were randomly selected to establish the 5/6Nx model. They were divided into three groups (hetero cKO group, hetero cKO+5/6Nx group, and hetero cKO+5/6Nx + MnTBAP group). At 9 weeks after the 5/6Nx surgery, the hetero cKO+5/6Nx + MnTBAP group were intraperitoneally injected with 10 mg/kg/d MnTBAP (MCE, HY-126397) diluted in saline, while the hetero cKO+5/6Nx group received intraperitoneal injections of saline at the same dose and frequency. The mice were sacrificed 2 h after the final injection.

### 2.5 Tissue histopathology

Paraffin-embedded renal tissue sections were used for hematoxylin-eosin (HE), periodic acid-Schiff (PAS), Masson's trichrome, immunofluorescence (IF) and immunohistochemical (IHC) staining as previously described [24,[43], [44], [45]]. The antibodies used for IHC staining and IF are listed in Table S2. All the signals of HE, PAS, Masson, and IHC staining were detected with Olympus BX51 microscope (Olympus, Center Valley, PA) or KFBIO KF-PRO-020 digital slide scanner (KFBIO, Ningbo, China). The signals of IF staining were detected with Zeiss LSM 710 laser scanning confocal microscope (Carl Zeiss AG, Oberkochen, Germany) or Leica STELLARIS 5 laser scanning confocal microscope (Leica Microsystems CMS GmbH, Wetzlar, Germany). A minimum of five fields at × 400 magnification for each kidney tissue sample were examined via microscopy. A modified scoring system was used to assess the degree of fibrosis from 0 to 5 (0, no fibrosis; 1, fibrosis involving 5–10 % of the area; 2, fibrosis involving 10–25 % of the area; 3, fibrosis involving 25–50 % of the area; 4, fibrosis involving 50–75 % of the area; 5, fibrosis involving 75–100 % of the area) [46]. To ensure the objectivity of the evaluation, all histological scoring and quantitative analysis were performed under single-blind conditions, where the analysts were unaware of the experimental groupings.

### 2.6 Transmission electron microscopy

Samples were fixed with electron microscopy fixative (Servicebio, G1124) to evaluate the morphological changes in podocytes in kidney tissues and the intracellular ultrastructure of podocytes. After postfixing with 1 % OsO4 in 0.1 mol/l phosphate buffer, ultrathin sections (60 nm) were cut on a microtome, placed on copper grids, stained with uranyl acetate and lead citrate. The samples were examined with an electron microscope (JEOL JEM-1010; Tokyo, Japan), as previously described [47]**.**

### 2.7 Cell culture experiments

Mouse aortic endothelial cells (MAECs) were purchased from Jennio Biotech Co., Ltd (Guangdong, China), mouse podocytes (MPCs) were obtained from Ding Jie, Peking University (Beijing, China), and mouse mesangial cells (MCs) were obtained from the China Center for Type Culture Collection (Wuhan, China). Primary human arterial endothelial cells (HAECs) were purchased from Lixing Biotech Co., Ltd (Nanjing, China) and immortalized HAECs were purchased from Immocell Biotech Co., Ltd (Xiamen, China). MAECs and MCs were cultured in DMEM supplemented with 10 % FBS and 1 % penicillin‒streptomycin. MPCs were cultured in RPMI 1640 supplemented with 10 % FBS and 1 % penicillin‒streptomycin. HAECs were cultured in the complete human aortic endothelial cell medium. All the cells were cultured at 37 °C with 5 % CO_2_. The cell lines were identified by STR analysis. The primary cells were identified by IF assay.

Cells were transfected with LONP1 or SOD2 plasmids or an shRNA targeting LONP1 using Lipofectamine 2000 (Invitrogen, Thermo Fisher Scientific, Halethorpe, MD; 11668019) according to the manufacturer's instructions and then stimulated with 0.1 μM angiotensin II (Ang II) (Sigma-Aldrich, St. Louis, MO, USA; 4474-91-3) or control buffer for 24 h. The human LONP1 shRNA (sc-97290-SH) was purchased from Santa Cruz, and the mouse LONP1 shRNA (sequence: GCTGCATACAAGATCGTAA) was obtained from RiboBio (Guangzhou, China).

Cells were treated with 50 μM MnTBAP (MCE, HY-126397) for 12 h and then transfected with shRNA of LONP1 or treated with Ang II. MCs and MPCs were treated with MAECs culture supernatant for 24 h.

### 2.8 Western blot assays

Cells and renal tissues were lysed in RIPA buffer (Beyotime Biotechnology, P0013K) containing a protease inhibitor cocktail (Roche, 0469313200). A BCA protein assay kit (Beyotime Biotechnology, P0012) was used to determine the protein concentration. Lysates were separated by SDS‒PAGE. The antibodies used for Western blot are listed in Table S2. The blots were visualized with a gel imaging system (Bio-Rad, USA). The densitometry analysis was performed using Image Lab Software (Bio-Rad, USA).

### 2.9 RNA extraction and real-time quantitative PCR (qRT‒PCR)

RNA was extracted from cells with TRIzol reagent (TaKaRa, 9109). Reverse transcription was performed using HiScript II Q RT SuperMix for qPCR (Vazyme, R222-01) according to the manufacturer's instructions. qRT‒PCR was performed on a Roche LightCycler 96 assay system (Roche, Switzerland) using AceQ qPCR SYBR Green Master Mix (Vazyme, Q131-02). The temperature cycling conditions were 95 °C for 10 min, followed by 40 cycles of 95 °C for 15 s, 60 °C for 1 min, 95 °C for 10 s, 65 °C for 1 min, 97 °C for 1 s, and 37 °C for 30 s. The relative mRNA expression was normalized to the relative expression of GAPDH or 18S RNA and calculated via the ΔΔCt method. Primer 5.0 software (http://Frodo.wi.mit.edu) was used to design the primers, and their sequences are shown in Table S3.

### 2.10 MDA assay

The MDA assay was performed using a lipid oxidation (MDA) detection kit (Beyotime Biotechnology, S0131S). Briefly, cells were lysed on ice and centrifuged at 12,000 rpm for 10 min (4 °C). The cell supernatants were performed MDA assay following the manufacturer's instructions. The concentration of MDA was calculated from a standard curve, and MDA levels were calculated by MDA concentration (μmol/L) ∗ urine volume (L).

### 2.11 Reactive oxygen species (ROS) assay

Cells after treatment were incubated in the serum-free DMEM with 10 μM DCFH-DA (Beyotime Biotechnology, S0033S) at 37 °C in the dark for 20 min. Next, the cells were washed by serum-free DMEM for 3 times, and collected after trypsin digestion. To ensure the accuracy and comparability of the data, a fixed total number of cells was collected for each sample during the data collection phase. The mean fluorescence intensity (MFI) of DCF fluorescence, indicative of the average ROS level, was measured using a CytoFLEX flow cytometer (Beckman Coulter, Brea, CA, USA). Data analysis was performed with CytExpert software.

### 2.12 MitoSOX assay

Cells after treatment were incubated in the preheated serum-free DMEM with 500 nM MitoSOX working solution (Invitrogen, M36008) at 37 °C in the dark for 30 min. Next, the cells were washed by preheated serum-free DMEM for 3 times, and collected after trypsin digestion. The average fluorescence intensity of MitoSOX level was measured using CytoFLEX (Beckman Coulter, Brea, CA, USA), and data were analyzed with CytExpert.

### 2.13 MitoTracker staining

The MitoTracker staining was performed using MitoTracker Red CMXRos kit (Meilun Biotechnology Co., Ltd, MB6046). According to the manufacturer's instructions, cells after treatment were incubated with the preheated serum-free DMEM with 200 nM MitoTracker working solution at 37 °C in the dark for 20 min, and washed by preheated serum-free DMEM for 3 times. After fixation, transparency, and DAPI staining, the fluorescence signal of MitoTracker was observed, photographed and recorded by Zeiss LSM 710 laser scanning confocal microscope (Carl Zeiss AG, Oberkochen, Germany) or Leica STELLARIS 5 laser scanning confocal microscope (Leica Microsystems CMS GmbH, Wetzlar, Germany).

### 2.14 Extraction of mouse glomerular endothelial cells

Male mice were euthanized by cervical dislocation, and immersed in 75 % ethanol for 5 min. Kidneys extracted from mice were quickly immersed in pre-chilled PBS containing 1 % antibiotics and washed twice. The renal cortex was separated and cut into small pieces of 1–3 mm, subsequently digested in the 1 mg/ml collagenase II (containing DNase I) (Solarbio, C8150) and 1.2 U/ml dispase II (Roche, 65558200) at 37 °C for 1.5 h. The strain tissue-solution mix was filtered through a 100 μm cell strainer into a sterile container. After secondary digestion with collagenase II (containing DNase I) and dispase II, the glomeruli containing solution was filtered through a 40 μm cell strainer and centrifuged at 1000 rpm for 2 min. 3–5 vol of red blood cell lysis buffer (Beyotime, C3702) were added to the sediment. After centrifugation at 300*g* for 10 min, CD31 microbeads (Miltenyi Biotec, 130-097-418) were used to separate endothelial cells according to the instructions [48,49].

### 2.15 EdU staining

The EdU-488 Cell Proliferation Detection Kit (Beyotime Biotechnology, C0071S) was applied to perform Edu staining. The cells were incubated with serum-free DMEM and equal volume of EdU working solution at 37 °C in the dark for 2 h. The cells were subsequently fixed, permeabilized, and washed. Next, the cells were incubated with Click reaction mixture in the dark for 30 min, and incubated with Hoechst 33342 (1:1000) for 10 min. Finally, fluorescence detection was performed under a Zeiss LSM 710 laser scanning confocal microscope (Carl Zeiss AG, Oberkochen, Germany), and positive stained cells were photographed and recorded.

### 2.16 Co-immunoprecipitation (Co-IP)

Cells were lysed with IP cell lysis buffer (Beyotime Biotechnology, P0013J). Protein A/G magnetic beads (MCE, HY-K0202) were premixed with the whole-cell lysate and incubated at 4 °C for 2 h. Then, the whole-cell lysate was mixed with mouse anti-Flag antibody (20 μg/ml) or normal mouse IgG and incubated at 4 °C for 2 h. Newly treated beads were then added and mixed with whole-cell lysate overnight at 4 °C. On the second day, the beads were washed 4 times and eluted with 1 × SDS loading buffer (Beyotime Biotechnology, P0015L) for Western blot analysis.

### 2.17 Annexin-V-FITC and PI staining

Cell suspension was stained with FITC-Annexin V and PI according to the manufacturer's instructions (BD Pharmingen, 6119909). Flow cytometry was used to analyzing cell apoptosis by CytoFLEX (Beckman Coulter, Brea, CA, USA), and data were analyzed with CytExpert.

### 2.18 Seahorse XF analysis

The mitochondrial function was assessed using the Seahorse XF Cell Mito Stress Test Kit (Agilent, 103015-100) on a Seahorse XF Pro Analyzer. Prior to the assay, 10,000 primary HAECs per well and 15,000 MAECs per well were seeded into an XFe96 cell culture microplate and transfected with LONP1 plasmid or shRNA for LONP1, and treated with Ang II for 24 h. The sensor cartridge was hydrated with XF calibrant solution (Agilent, 100840-000) at 37 °C overnight. 1 h before the assay, the cell medium was replaced with pre-warmed XF assay medium (PH 7.4) (Agilent, 103575-100) supplemented with 10 mM glucose (Agilent, 103577-100), 1 mM pyruvate (Agilent, 103578-100), and 2 mM glutamine (Agilent, 103579-100), and the plate was incubated at 37 °C in a non-CO_2_ incubator. For the Mitochondrial Stress Test, 1.5 μM oligomycin (Port A), 1.0 μM FCCP (Port B), and 0.5 μM rotenone/antimycin A (Port C) were loaded into the corresponding ports. The assay protocol consisted of a 30 min calibration period followed by 3 baseline measurement cycles and 3 measurement cycles after each injection. After the assay, the oxygen consumption rate (OCR) values were normalized to cell counts (pmol/min/10^3^ cells) [50].

### 2.19 Ubiquitination analysis

Cells were co-transfected with LONP1-Flag, SOD2-HA, or the Ub-MYC plasmid for 24 h. Ubiquitin proteasome inhibitor was added to the cells for 4 h before the collection. The cells were lysed on ice and the resulting cell lysate was immunoprecipitated with an anti-HA antibody. Changes in MYC tag protein expression were observed by SDS-PAGE.

### 2.20 Transcriptome analysis using RNA-seq

Transcriptome analysis using RNA-sequencing (RNA-seq) was performed as published [51]. Raw data of transcriptome analysis have been deposited on the Sequence Read Archive (SRA) database (https://www.ncbi.nlm.nih.gov/sra/) with the accession number PRJNA1174991 and PRJNA1176378.

### 2.21 Molecular docking

Molecular docking was performed on the screened receptor protein peptide complexes using the Gromacs 2020 software package. The AMBER99SB-ILDN force field parameters were used for the protein and the TIP3P dominant water model was selected. The correlation analysis was performed using the trjconv module. The binding free energy of the ligands and proteins was calculated using the gMMPBSA method with the Gromacs 2020 program.

### 2.22 Surface plasmon resonance (SPR)

The recombinant human LONP1 protein and SOD2 protein for detecting molecular interactions were obtained from General Biol (Anhui, China). The binding affinity of LONP1 for SOD2 was determined using a Biacore T200 SPR biosensor system with a CM5 chip (GE Healthcare, Chicago, IL). The recombinant human LONP1 protein was covalently coupled to the activated CM5 chip. Recombinant human SOD2 protein was diluted from 10 to 0.3125 μM in PBS/0.01 % Tween 20 buffer and injected at 20 μl/min. The association and dissociation time were set to 240 s and 420 s respectively. Equilibrium constants (KDs) were calculated using Biacore T200 evaluation software (GE Healthcare, Chicago, IL).

### 2.23 Biolayer interferometry (BLI)

The recombinant human LONP1 protein and SOD2 protein for detecting molecular interactions were obtained from General Biol (Anhui, China). The binding affinity of LONP1 for SOD2 was determined using an Octet R8 instrument (Sartorius, Germany) with a high-precision streptavidin 2.0 biosensor. The activated chip was placed into the analysis buffer containing the biotinylated LONP1 solidified material for 10 min. And the chip bound to the biotinylated LONP1 solidified material reacted with the SOD2 analyte at 0, 2.5, 5, and 10 μM for 90 s. Equilibrium constants (KDs) were calculated through a steady-state fitting analysis.

### 2.24 Microscale thermophoresis (MST)

The recombinant human LONP1 protein and SOD2 protein for detecting molecular interactions were obtained from General Biol (Anhui, China). The binding affinity of LONP1 for SOD2 was measured using a Monolith NT.115 instrument (NanoTemper Technologies, Germany). The LONP1 protein was fluorescently labelled with the NHS Label Kit (NanoTemper Technologies, MO-L011) according to the manufacturer's protocol. SOD2 protein was diluted to the indicated concentrations (from 2.85 μM to 22.8 μM) and incubated with 86 nM labelled LONP1 protein for 10 min. The samples were loaded into NanoTemper glass capillaries, and microthermophoresis was performed with 80 % light-emitting diode power and 80 % MST. Equilibrium constants (KDs) were calculated with NanoTemper software.

### 2.25 Statistical analysis

The data are described as the means ± SEMs. GraphPad Prism software version 9.3 (San Diego, CA) was utilized for conducting statistical analyses. A two-tailed *t*-test was used to compare differences between two groups. A one-way analysis of variance (ANOVA) was used to compare differences among multiple groups. The quantification of immunostaining intensity was performed utilizing the ImageJ software. ∗*P* < 0.05, ∗∗*P* < 0.01, ∗∗∗*P* < 0.001, and ∗∗∗∗*P* < 0.0001. *P* = 0.05 was considered statistically significant.

## 3 Results

### 3.1 LONP1 is downregulated in glomerulosclerotic kidney tissues and damaged endothelial cells

**Fig. 1:**
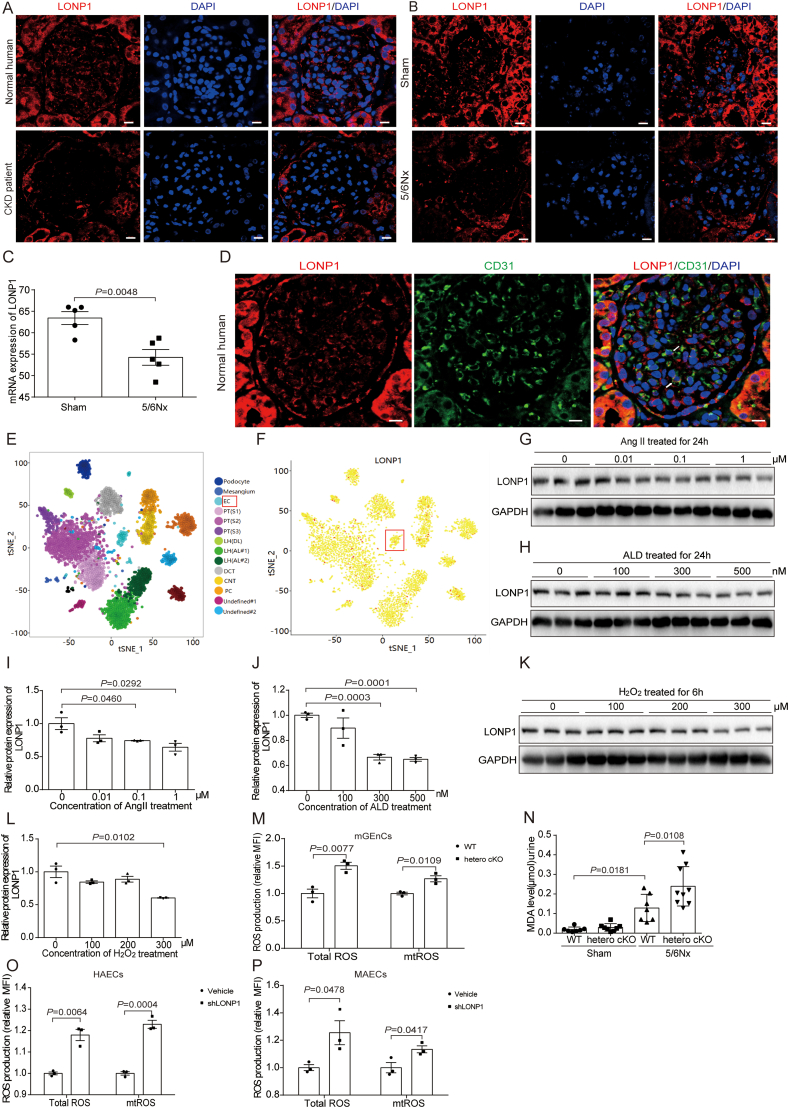
LONP1 is downregulated in glomerulosclerotic kidney tissues and damaged endothelial cells. A) IF staining of LONP1 in the normal human and CKD patient kidneys. Scale bar, 10 μm. B) IF staining of LONP1 in the sham and 5/6Nx mice kidneys. Scale bar, 10 μm. C) RNA sequencing analysis showed the mRNA level of LONP1 in the kidney of sham and 5/6Nx mice (*n* = 5). D) IF staining of LONP1 and CD31 in the normal human kidneys. LONP1 (red), CD31 (green), DAPI (blue), and co-localized position (white arrows). Scale bar, 10 μm. E, F) Humphreyslab database (http://humphreyslab.com/SingleCell/) showed the expression of LONP1 in various cell populations of healthy human kidney tissues. G, I) LONP1 expression of the MAECs induced by Ang II at concentration of 0, 0.01, 0.1, 1 μM was determined by Western blot. GAPDH was used as a control (*n* = 3). Dot plots represent quantitative densitometric data from Western blot. H, J) LONP1 expression of the MAECs induced by ALD at concentration of 0, 100, 300, 500 nM was determined by Western blot. GAPDH was used as a control (*n* = 3). Dot plots represent quantitative densitometric data from Western blot. K, L) LONP1 expression of the MAECs induced by H_2_O_2_ at concentration of 0, 100, 200, 300 μM was determined by Western blot. GAPDH was used as a control (*n* = 3). Dot plots represent quantitative densitometric data from Western blot. M, O, P) Quantification of the MFI of DCF (indicating total reactive oxygen species [ROS]; *n* = 3) and mitoSOX (indicating mitochondrial ROS [mtROS]; *n* = 3) using flow cytometry in glomerular vascular endothelial cells extracted from WT and hetero cKO mice (M), HAECs (O), and MAECs (P). N) MDA level in the urine of WT and hetero cKO mice after 5/6Nx (*n* = 7–9). EC, Endothelial cell; CKD, chronic kidney disease; 5/6Nx, 5/6 nephrectomy; Ang II, Angiotensin; ALD, Aldosterone; H_2_O_2_, hydrogen peroxide; mtROS, mitochondrial ROS; mGEnCs, Mouse Glomerular Endothelial Cells; MAECs, Mouse aortic endothelial cells; HAECs, Human aortic endothelial cells.

IF staining showed that the expression of LONP1 in the glomeruli of CKD patients and 5/6Nx mice model was significantly decreased, compared with that in the controls (Fig. 1A and B). RNA sequencing revealed the same expression trend as the IF staining of sham and 5/6Nx mice (Fig. 1C). Through a kidney single-cell database (http://humphreyslab.com/SingleCell/), we found that LONP1 was expressed in multiple cell populations of healthy human kidney tissue, including endothelial cell populations (Fig. 1E and F). It was further verified by coimmunostaining for LONP1 and CD31, which is a specific marker of vascular endothelial cell (Fig. 1D), and the results showed that LONP1 was downregulated by 35 % in pediatric CKD samples, with inter-patient variability (27 %–44 % range) likely reflecting disease heterogeneity (Supplementary Fig. 1A and 1B). Besides, the reduction of LONP1 was also observed in podocytes and renal tubular epithelial cells in 5/6Nx mice (Supplementary Fig. 1C–1F). In vitro, the expression of LONP1 was downregulated in the endothelial cell injury models induced by different concentrations of Angiotensin II (Ang II), aldosterone (ALD), and hydrogen peroxide (H_2_O_2_) in vitro (Fig. 1G-L). It indicated an important role of endothelial cell LONP1 in the process of CKD.

LONP1 is a key enzyme for maintaining mitochondrial redox balance, although homozygous knockout of LONP1 leads to embryonic lethality [27]. We extracted glomerular endothelial cells from hetero cKO mice and detected significant increases in total ROS and mitochondrial ROS (mtROS) levels compared with those in WT mice (Fig. 1M). The malondialdehyde (MDA) level in urine was significantly higher in the hetero cKO 5/6Nx mice than those in the wild-type (WT) 5/6Nx mice (Fig. 1N). Similarly, knockdown of LONP1 in both HAECs and MAECs significantly aggravated the release of total ROS and mtROS (Fig. 1O and P).

### 3.2 Endothelial LONP1 directly binds to SOD2 to alleviate oxidative stress

**Fig. 2:**
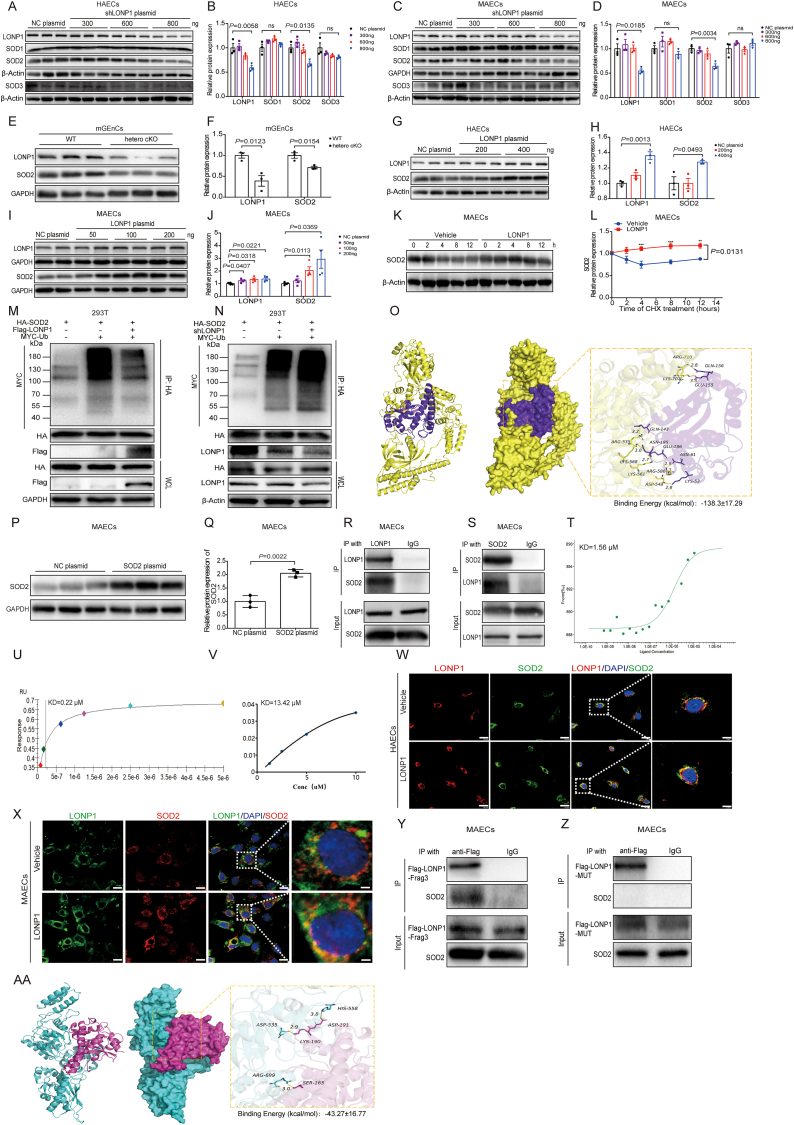
Endothelial LONP1 directly binds to SOD2 to alleviate oxidative stress. A, B) Western blot of LONP1, SOD1, SOD2, and SOD3 in primary HAECs transfected with NC plasmid, 300, 600, and 800 ng shRNA of LONP1 for 24 h. β-Actin was used as a control (*n* = 3). Dot plots represent quantitative densitometric data from Western blot. C, D) Western blot of LONP1, SOD1, SOD2, and SOD3 in MAECs transfected with NC plasmid, 300, 600, and 800 ng shRNA of LONP1 for 24 h. GAPDH was used as a control of LONP1, SOD1 and SOD2, β-Actin was used as a control of SOD3 (*n* = 3). Dot plots represent quantitative densitometric data from Western blot. E, F) Western blot of LONP1 and SOD2 in glomerular vascular endothelial cells extracted from WT and hetero cKO mice. GAPDH was used as a control (*n* = 3). Dot plots represent quantitative densitometric data from Western blot. G, H) Western blot of LONP1 and SOD2 in primary HAECs transfected with NC plasmid, 200, and 400 ng LONP1 overexpression plasmid for 24 h. β-Actin was used as a control (*n* = 3). Dot plots represent quantitative densitometric data from Western blot. I, J) Representative Western blot of LONP1 and SOD2 in MAECs transfected with NC plasmid, 50, 100, and 200 ng LONP1 overexpression plasmid for 24 h. GAPDH was used as a control (*n* = 4). Dot plots represent quantitative densitometric data from Western blot. K, L) Representative Western blot of SOD2 in MAECs transfected with NC plasmid and LONP1 overexpression plasmid, and treated with CHX for 0, 2, 4, 8, 12 h. β-Actin was used as a control (*n* = 3). Dot plots represent quantitative densitometric data from Western blot. M) Ubiquitination experiment of 293T cells transfected with LONP1 overexpression plasmid, SOD2 overexpression plasmid, and Ub plasmid. N) Ubiquitination experiment of 293T cells transfected with shRNA for LONP1, SOD2 overexpression plasmid, and Ub plasmid. O) Molecular interactions between the LONP1 and SOD2 by Molecular Dynamics Analysis. P, Q) Western blot of SOD2 in MAECs transfected with NC plasmid and SOD2 overexpression plasmid. GAPDH was used as a control (*n* = 3). Dot plots represent quantitative densitometric data from Western blot. R, S) Co-IP analysis of interaction between LONP1 and SOD2 in MAECs. T-V) Physical interaction between LONP1 and SOD2 protein performed by MST (T), SPR (U), and BLI (V). W) IF co-localization analysis of LONP1 and SOD2 in primary HAECs. LONP1 (red), SOD2 (green), DAPI (blue), and co-localized position (White dashed box). Scale bar, 10 μm. X) IF co-localization analysis of LONP1 and SOD2 in MAECs. LONP1 (green), SOD2 (red), DAPI (blue), and co-localized position (White dashed box). Scale bar, 10 μm. Y) Co-IP analysis of interaction between LONP1-Frag3 and SOD2 in MAECs. Z) Co-IP analysis of interaction between LONP1-MUT and SOD2 in MAECs. AA) Molecular interactions between the LONP1-MUT and SOD2 by Molecular Dynamics Analysis. NC, Negative Control; LONP1-Frag3, LONP1-Fragment3; LONP-MUT, LONP1-mutant; mGEnCs, Mouse Glomerular Endothelial Cells; MAECs, Mouse aortic endothelial cells; HAECs, Human aortic endothelial cells.

We investigated the expression of SOD1, SOD2, and SOD3 after LONP1 deficiency to verify the correlation between LONP1 and SOD. Western blot revealed that SOD2 expression was significantly decreased, while SOD1 and SOD3 was not significantly changed after knocking down LONP1 in both HAECs and MAECs (Fig. 2A-D, Supplementary Fig. 2A and 2B). Additionally, SOD2 expression was significantly reduced in glomerular endothelial cells isolated from endothelial cell-specific heterozygous LONP1 knockout mice (Fig. 2E and F). In contrast, after overexpressing LONP1, SOD2 levels were significantly elevated in both HAECs and MAECs (Fig. 2G-J, Supplementary Fig. 2C and 2D). Protein degradation experiments further revealed an obvious slow degradation rate of SOD2 after overexpressing LONP1 (Fig. 2K and L). To further clarify the mechanism by which LONP1 stabilizes SOD2 expression, we performed ubiquitination experiments after overexpressing or knocking down LONP1. It indicated that the overexpression of LONP1 could significantly reduce the level of ubiquitinated SOD2, and vice versa (Fig. 2M and N). As shown in Fig. 2O, SOD2 bound the amino acid residues ARG535, LYS568, LYS561, ASP543, ARG586, LYS707 and ARG710 on LONP1, with a binding energy of −138.30 ± 17.29 kcal/mol. We transfected the SOD2 overexpression plasmid into MAECs, and Western blot showed that SOD2 expression increased by approximately 51.31 % (Fig. 2P and Q). Co-IP experiment revealed that LONP1 interacted with SOD2 (Fig. 2R and S). The binding affinities (KD values) between the recombinant SOD2 protein and LONP1 protein was quantified to be 1.56 μM, 0.22 μM, and 13.42 μM by MST, SPR, and BLI assays respectively (Fig. 2T-V). And IF staining showed co localization of LONP1 and SOD2 in both HAECs and MAECs (Fig. 2W and X). Co-IP subsequently revealed the interaction between SOD2 and the truncated LONP1 (named LONP1-Frag3), which contains the putative binding sites (Fig. 2Y). However, Co-IP did not show an interaction between SOD2 and the LONP1 mutant (named LONP1-MUT), in which each binding site was mutated to the amino acid residue with the lowest binding affinity (Fig. 2Z). As shown in Fig. 2AA, the binding energy of the LONP1 mutant with SOD2 decreased from −138.30 ± 17.29 kcal/mol to −43.27 ± 16.77 kcal/mol by molecular docking. These results suggested that endothelial LONP1 could stabilize SOD2 expression by protecting against SOD2 ubiquitination, thereby reducing the abnormal release of ROS.

### 3.3 Endothelial cell-specific heterozygous knockout of LONP1 aggravates glomerulosclerosis and inflammation in a CKD model induced by 5/6Nx

Hetero cKO mice were generated via the Cre/LoxP recombinase system (Supplementary Fig. 3A and 3B). Western blot showed that compared with the WT mice, the expression of LONP1 in the glomerular endothelial cells of hetero cKO mice was significantly decreased, while the expression of CD31 was significantly increased (Supplementary Fig. 3C–3E). It had no significant differences in the morphology of the aorta, heart, and kidney performing HE staining, as well as the serum BUN and Cr and urine microalbumin levels between WT and hetero cKO mice (Supplementary Fig. 3F–3L).

**Fig. 3:**
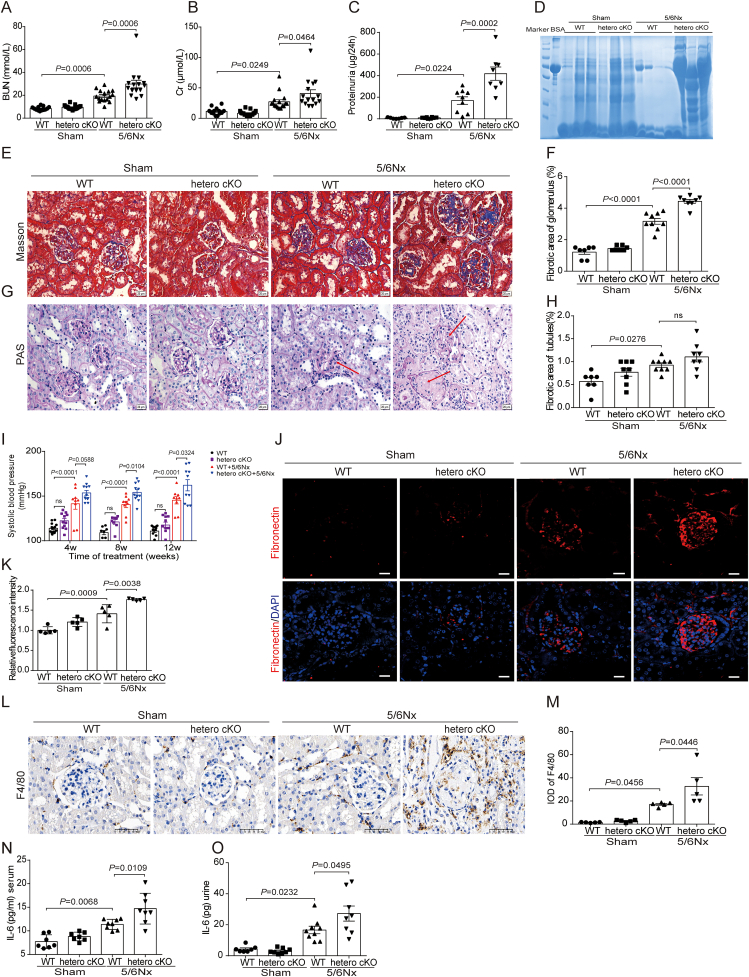
Heterozygous knockout of endothelial cell LONP1 aggravates glomerulosclerosis and inflammation in CKD induced by 5/6Nx. A, B) Analysis of BUN (A) and Cr (B) (*n* = 13–17). C) ELISA of urinary microalbumin (*n* = 7–9). D) Coomassie blue staining of representative urine samples by sodium dodecyl sulfate polyacrylamide gel electrophoresis. E) Masson's trichrome staining of WT and hetero cKO mice after 5/6Nx (*n* = 7–9, Scale bar, 20 μm). F, H) Fibrotic area of glomerulus (F) and tubules (H) statistics of Masson's trichrome staining in WT and hetero cKO mice after 5/6Nx (*n* = 7–9). G) PAS staining of WT and hetero cKO mice after 5/6Nx (*n* = 7–9, Scale bar, 20 μm). Pathological changes of glomerulosclerosis (red arrows). I) Systolic blood pressure of WT and hetero cKO mice after 5/6Nx 4 w, 8 w, and 12 w detected by tail-cuff (*n* = 8–15). J) IF staining of Fibronectin in WT and hetero cKO mice after 5/6Nx (*n* = 5, Scale bar, 20 μm). K) Relative fluorescence intensity statistics of IF staining (*n* = 5). L) IHC staining of F4/80 in WT and hetero cKO mice after 5/6Nx (*n* = 5, Scale bar, 50 μm). M) IHC semi-quantitative IOD analysis of F4/80 (*n* = 5). N, O) The IL-6 levels of serum (N) (*n* = 7–8) and urine (O) (*n* = 7–9) in WT and hetero cKO mice after 5/6Nx were detected by ELISA. IOD, Integral Optical Density.

Further studies revealed that heterozygous knockout of LONP1 in endothelial cells significantly aggravated 5/6Nx-induced renal injury and fibrosis. As shown in Fig. 3A, B, and 3I, the serum BUN and Cr levels, and systolic blood pressure of the hetero cKO 5/6Nx mice were significantly higher than those of the WT 5/6Nx mice. ELISA (Fig. 3C) and Coomassie blue staining (Fig. 3D) revealed that the urine protein content of the hetero cKO 5/6Nx mice was significantly higher than that of the WT 5/6Nx mice. Masson's trichrome staining (Fig. 3E, F, 3H) and PAS staining (Fig. 3G) revealed that the heterozygous knockout of LONP1 in endothelial cells further exacerbated glomerular basement membrane (GBM) thickening, mesangial cell and matrix proliferation, and collagen fibre deposition in WT 5/6Nx mice. Compared with those of WT 5/6Nx mice, IF staining confirmed that the expression of the fibrosis marker fibronectin in the kidneys of hetero cKO 5/6Nx mice was significantly elevated (Fig. 3J and K). The expression of F4/80, which is a marker of macrophage maturation and participates in inflammatory cell infiltration, was significantly increased in the kidneys of hetero cKO 5/6Nx mice compared with those in WT 5/6Nx mice via IHC staining (Fig. 3L and M). Our results also revealed that the level of the IL-6 was significantly increased in the serum and urine of hetero cKO 5/6Nx mice compared with WT 5/6Nx mice (Fig. 3N and O).

### 3.4 LONP1 knockdown promotes Ang II-induced mitochondrial dysfunction and inflammatory damage in vascular endothelial cells

**Fig. 4:**
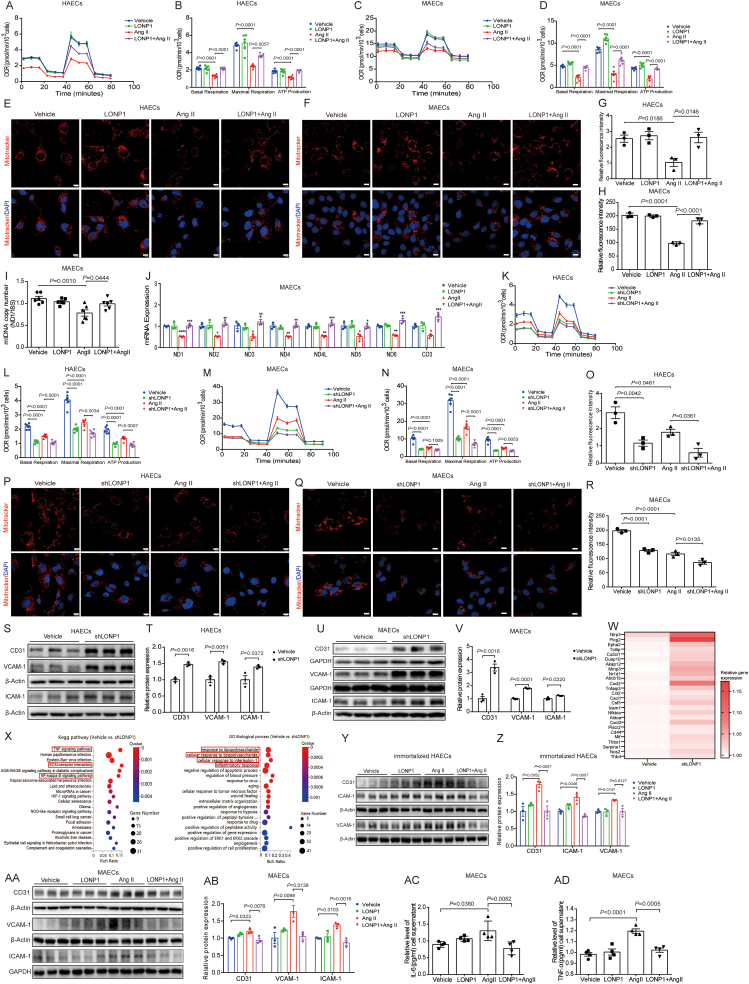
Knockdown of LONP1 promotes Ang II-induced mitochondrial dysfunction and inflammatory damage in vascular endothelial cells. A) OCR of primary HAECs transfected with LONP1 overexpression plasmid and treated with Ang II (*n* = 6). B) Quantification of basal respiratory, maximal respiratory, and ATP production in Fig. A. C) OCR of MAECs transfected with LONP1 overexpression plasmid and treated with Ang II (*n* = 6). D) Quantification of basal respiratory, maximal respiratory, and ATP production in Fig. C. E, F) IF staining of MitoTracker in primary HAECs (E) and MAECs (F) transfected with LONP1 overexpression plasmid and treated with Ang II (*n* = 3, Scale bar, 10 μm). G, H) Relative fluorescence intensity of MitoTracker in primary HAECs (G) and MAECs (H) transfected with LONP1 overexpression plasmid and treated with Ang II (*n* = 3). I) mitochondrial DNA (mtDNA) was assessed by quantitative polymerase chain reaction, and the number of mitochondrial genome copies (detected by the ND1 primer) were normalized to 18S ribosomal RNA (*n* = 6). J) mRNA expression of genes encoded by the mitochondrial genome in MAECs transfected with LONP1 overexpression plasmid and treated with Ang II (*n* = 3). K) OCR of primary HAECs transfected with shRNA of LONP1 and treated with Ang II (*n* = 6). L) Quantification of basal respiratory, maximal respiratory, and ATP production in Fig. K. M) OCR of MAECs transfected with shRNA of LONP1 and treated with Ang II (*n* = 6). N) Quantification of basal respiratory, maximal respiratory, and ATP production in Fig. M. O) Relative fluorescence intensity of MitoTracker in primary HAECs transfected with shRNA of LONP1 and treated with Ang II (*n* = 3). P, Q) IF staining of MitoTracker in primary HAECs (P) and MAECs (Q) transfected with shRNA of LONP1 and treated with Ang II (*n* = 3, Scale bar, 10 μm). R) Relative fluorescence intensity of MitoTracker in MAECs transfected with shRNA of LONP1 and treated with Ang II (*n* = 3). S, T) Western blot of CD31, VCAM-1, and ICAM-1 in primary HAECs transfected with shRNA of LONP1 for 24 h. β-Actin was used as a control (*n* = 3). Dot plots represent quantitative densitometric data from Western blot. U, V) Western blot of CD31, VCAM-1, and ICAM-1 in MAECs transfected with shRNA of LONP1 for 24 h. β-Actin was used as a control of ICAM-1, GAPDH was used as a control of CD31 and VCAM-1 (*n* = 3). Dot plots represent quantitative densitometric data from Western blot. W) Heatmap of upregulated inflammatory factors between the Vehicle and shLONP1 groups. X) Kyoto Encyclopedia of Genes and Genomes (KEGG) enrichment analysis and Gene Ontology (GO) analysis showed that differential signaling pathways were identified by the differential genes between the Vehicle and shLONP1 groups. Y, Z) Western blot of CD31, VCAM-1, and ICAM-1 in immortalized HAECs transfected with LONP1 plasmid and treated with Ang II. β-Actin was used as a control (*n* = 3). Dot plots represent quantitative densitometric data from Western blot. AA, AB) Western blot of CD31, VCAM-1, and ICAM-1 in MAECs transfected with LONP1 plasmid and treated with Ang II. β-Actin was used as a control of CD31 and VCAM-1, GAPDH was used as a control of ICAM-1 (*n* = 3). Dot plots represent quantitative densitometric data from Western blot. AC, AD) The IL-6 (AC) and TNF-α (AD) levels of cell medium in MAECs transfected with LONP1 plasmid and treated with Ang II were detected by ELISA (*n* = 4).

To further verify the role of LONP1 in mitochondrial function, we measured the OCR in HAECs and MAECs using the Seahorse XF Analyzer. Compared to controls, Ang II treatment markedly suppressed mitochondrial respiration and ATP production in both HAECs and MAECs, while LONP1 overexpression effectively suppressed this effect (Fig. 4A-D). Subsequently, we observed that the reduction in mitochondrial tracer probe fluorescence intensity induced by Ang II in HAECs and MAECs was reversed by LONP1 overexpression (Fig. 4E-H). We consistently found that LONP1 overexpression prevented the reduction in mtDNA copy number and the mRNA levels of multiple mitochondrial-encoded genes induced by Ang II (Fig. 4I and J). Conversely, LONP1 knockdown caused a decrease in OCR and mitochondrial tracker probe fluorescence intensity, and the deficiency of LONP1 could further intensified these mitochondrial impairment events induced by Ang II in both MAECs and primary HAECs (Fig. 4K-R). Moreover, we investigated whether mitochondrial dysfunction exacerbates inflammatory responses. The Western blot results revealed that LONP1 knockdown significantly increased the levels of the inflammation-related indicators CD31, vascular cell adhesion molecule-1 (VCAM-1) and intercellular cell adhesion molecule-1 (ICAM-1) in both HAECs and MAECs (Fig. 4S–V). Transcriptome analysis revealed that differentially expressed genes resulting from LONP1 knockdown in MAECs were enriched in the inflammation related pathways (Fig. 4X). And heatmaps revealed that 24 inflammatory factors, including NLRP3, Plcg2, Tollip, Dusp10, etc., were upregulated in MAECs after knocking down LONP1 (Fig. 4W). In contrast, the overexpression of LONP1 markedly alleviated the increased expression of CD31, VCAM-1, and ICAM-1 in both immortalized HAECs and MAECs induced by Ang II (Fig. 4Y–4AB). The ELISA also revealed that the secretion of IL-6 and tumor necrosis factor-α (TNF-α) in the supernatant of MAECs increased induced by Ang II, whereas the overexpression of LONP1 decreased these indicators (Fig. 4AC, 4AD). It suggested that LONP1 overexpression improved Ang II-induced mitochondrial dysfunction and the inflammatory responses.

### 3.5 SOD2 supplementation alleviates glomerulosclerosis and inhibits the vascular endothelial cells inflammation response

To further evaluated whether exogenous supplementation with SOD2 improved glomerulosclerosis and inhibited the endothelial cellular injury. We first applied SOD2 mimetic MnTBAP to the 5/6Nx model of wild-type mice. Masson's staining revealed collagen fiber deposition in 5/6Nx wild-type (WT) mice, which was markedly attenuated by MnTBAP treatment. This reduction in fibrosis was particularly evident from the decreased glomerulosclerosis (Supplementary Figs. 4A, 4B, 4D). Consistent with this, PAS staining further demonstrated that MnTBAP administration inhibited glomerular basement membrane thickening, as well as mesangial cell and matrix proliferation in 5/6Nx WT mice (Supplementary Fig. 4C). Supplementary Fig. 4E–4G showed that MnTBAP treatment effectively lowered the elevated levels of serum Cr, systolic blood pressure, and urine protein in 5/6Nx WT mice. Consistently, IF staining confirmed that MnTBAP significantly suppressed the upregulation of Fibronectin, particularly within glomeruli (Supplementary Fig. 4H and 4I). Furthermore, IHC staining showed a marked reduction in F4/80-positive cells in the kidneys of MnTBAP-treated 5/6Nx WT mice (Supplementary Fig. 4J and 4K).

**Fig. 5:**
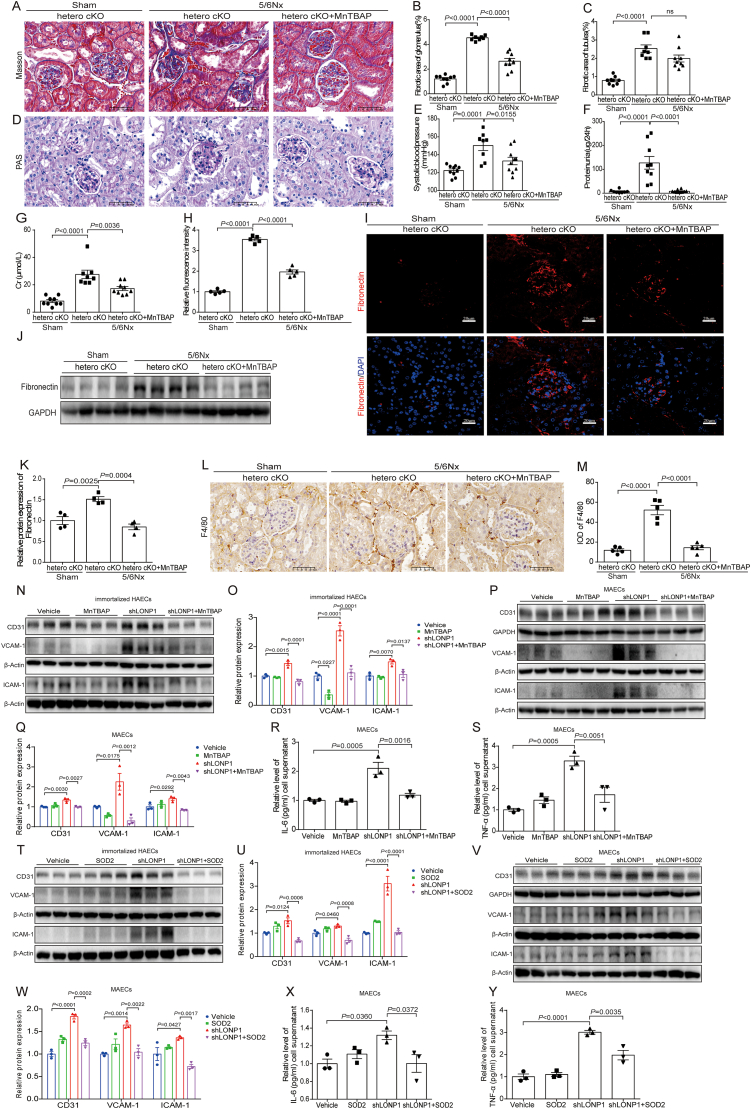
SOD2 supplementation alleviates glomerulosclerosis and inhibits the vascular endothelial cells inflammation response. A) Masson's trichrome staining in different groups (*n* = 8–9, Scale bar, 50 μm). B, C) Fibrotic area of glomerulus (B) and tubules (C) statistics of Masson's trichrome staining in different groups (*n* = 8–9). D) PAS staining in different groups (*n* = 8–9, Scale bar, 50 μm). E) Systolic blood pressure in different groups detected by tail-cuff (*n* = 8–10). F) ELISA of urinary microalbumin in different groups (*n* = 8–10). G) Analysis of Cr in different groups (*n* = 8–9). H) Relative fluorescence intensity statistics of IF staining of Fibronectin (*n* = 5). I) IF staining of Fibronectin in different groups (*n* = 5, Scale bar, 20 μm). J, K) Western blot of Fibronectin in different groups. GAPDH was used as a control (*n* = 4). Dot plots represent quantitative densitometric data from Western blot. L) IHC staining of F4/80 in different groups (*n* = 5, Scale bar, 50 μm). M) IHC semi-quantitative IOD analysis of F4/80 (*n* = 5). N, O) Western blot of CD31, VCAM-1, and ICAM-1 in immortalized HAECs transfected with shRNA of LONP1 and treated with MnTBAP. β-Actin was used as a control (*n* = 3). Dot plots represent quantitative densitometric data from Western blot. P, Q) Western blot of CD31, VCAM-1, and ICAM-1 in MAECs transfected with shRNA of LONP1 and treated with MnTBAP. GAPDH was used as a control of CD31, β-Actin was used as a control of VCAM-1 and ICAM-1 (*n* = 3). Dot plots represent quantitative densitometric data from Western blot. R, S) The IL-6 (R) and TNF-α (S) levels of cell medium in MAECs transfected with shRNA of LONP1 and treated with MnTBAP were detected by ELISA (*n* = 3). T, U) Western blot of CD31, VCAM-1, and ICAM-1 in immortalized HAECs co-transfected with shRNA of LONP1 and SOD2 overexpression plasmid. β-Actin was used as a control (*n* = 3). Dot plots represent quantitative densitometric data from Western blot. V, W) Western blot of CD31, VCAM-1, and ICAM-1 in MAECs co-transfected with shRNA of LONP1 and SOD2 overexpression plasmid. GAPDH was used as a control of CD31, β-Actin was used as a control of VCAM-1 and ICAM-1 (*n* = 3). Dot plots represent quantitative densitometric data from Western blot. X, Y) The IL-6 (X) and TNF-α (Y) levels of cell medium in MAECs co-transfected with shRNA of LONP1 and SOD2 overexpression plasmid were detected by ELISA (*n* = 3). IOD, Integral Optical Density.

Furthermore, we investigated the effect of MnTBAP on glomerulosclerosis of 5/6Nx models exacerbated by LONP1 knockdown. As shown by Masson's staining, we observed greater collagen deposition in the glomerulus of hetero cKO mice induced by 5/6Nx than that in the sham group, and MnTBAP could significantly reduce the collagen deposition (Fig. 5A-C). PAS staining revealed that endothelial cell-specific heterozygous knockout of LONP1 significantly aggravated pathological manifestations of glomerulosclerosis in 5/6Nx mice, whereas MnTBAP treatment reversed these pathological abnormalities (Fig. 5D). We found that the systolic blood pressure, urinary protein level, and serum Cr level in the hetero cKO 5/6Nx mice were significantly reduced after MnTBAP treatment (Fig. 5E-G). IF staining and Western blot consistently revealed that MnTBAP significantly decreased the expression of fibronectin in the glomerulus (Fig. 5H-K). Additionally, F4/80 was significantly decreased in the kidneys of hetero cKO 5/6Nx mice treated with MnTBAP, compared with those in the hetero cKO 5/6Nx mice via IHC staining (Fig. 5L and M).

In vitro, Western blot showed that MnTBAP significantly inhibited the increase of CD31, VCAM-1, and ICAM-1 in immortalized HAECs and MAECs after knocking down LONP1 (Fig. 5N-Q). Consistent with Western blot results, the inflammatory factors of IL-6 and TNF-α in the supernatant of MAECs induced by LONP1 knockdown were significantly decreased by MnTBAP treatment (Fig. 5R and S). We next co-transfected MAECs with shRNA of LONP1 and SOD2 overexpression plasmid, and Western blot showed that overexpression of SOD2 also suppressed the increased expression of CD31, VCAM-1, and ICAM-1, and elevated secretion of IL-6 and TNF-α in the supernatant induced by LONP1 knockdown (Fig. 5T-Y). Furthermore, SOD2 overexpression rescued approximately 67 %, 58 %, and 82 % of the elevations in ROS, the impairments in OCR, and the reductions in MitoTracker intensity caused by LONP1 knockdown, respectively (Supplementary Fig. 5). These results indicated that SOD2 supplementation could improve the effect of LONP1 deficiency on aggravating glomerulosclerosis and endothelial cell injury.

### 3.6 Knockdown of endothelial cell LONP1 facilitates the Ang II-induced activation and proliferation of mesangial cells

**Fig. 6:**
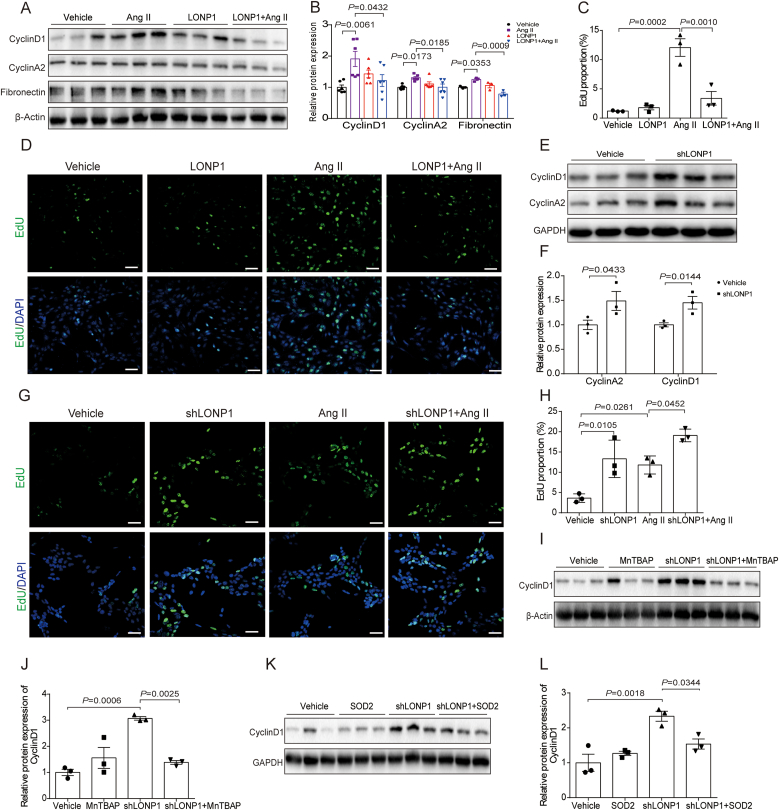
Knockdown of endothelial cell LONP1 facilitates Ang II-induced activation and proliferation of mesangial cells. A, B) Western blot of CyclinD1, CyclinA2 (*n* = 6), and Fibronectin (*n* = 3) in MCs induced by culture supernatant of MAECs transfected with LONP1 plasmid and treated with Ang II. β-Actin was used as a control. Dot plots represent quantitative densitometric data from Western blot. C, D) Representative micrographs with IF staining and the MFI of EdU in MCs induced by culture supernatant of MAECs transfected with LONP1 plasmid and treated with Ang II (*n* = 3, Scale bar, 40 μm). E, F) Western blot of CyclinD1 and CyclinA2 in MCs induced by culture supernatant of MAECs transfected with shRNA of LONP1. GAPDH was used as a control (*n* = 3). Dot plots represent quantitative densitometric data from Western blot. G, H) Representative micrographs with IF staining and the mean fluorescence intensity of EdU in MCs induced by culture supernatant of MAECs transfected with shRNA of LONP1 and treated with Ang II (*n* = 3, Scale bar, 40 μm). I, J) Western blot of CyclinD1 in MCs induced by culture supernatant of MAECs transfected with shRNA of LONP1 and treated with Ang II. β-Actin was used as a control (*n* = 3). Dot plots represent quantitative densitometric data from Western blot. K, L) Western blot of CyclinD1 in MCs induced by culture supernatant of MAECs co transfected with shRNA of LONP1 and SOD2 overexpression plasmid. GAPDH was used as a control (*n* = 3). Dot plots represent quantitative densitometric data from Western blot.

To further examine the indirect effects of LONP1 intervention in MAECs on MCs, we cultured MCs with MAECs culture supernatant. As shown in Fig. 6A and B the overexpression of LONP1 in MAECs significantly inhibited the expression of Cyclin D1, Cyclin A2, and Fibronectin in MCs induced by the Ang II-treated MAECs culture supernatant. EdU staining revealed that the number of EdU-positive MCs increased significantly in the Ang II group, but LONP1 overexpression dramatically mitigated this effect (Fig. 6C and D). In contrast, the expression of Cyclin D1 and Cyclin A2 in MCs, as well as the percentage of EdU-positive MCs were obviously increased in the shLONP1 group (Fig. 6E-H). In addition, the role of SOD2 supplementation in MAECs–MCs interaction was verified by Western blot. Compared with the shLONP1 group, the Cyclin D1 expression was significantly decreased in the MCs treated by culture supernatant of MAECs which were applied MnTBAP or transfected with SOD2 plasmid after knocking down LONP1 (Fig. 6I-L).

### 3.7 Knockdown of endothelial cell LONP1 facilitates Ang II-induced podocytes injury

**Fig. 7:**
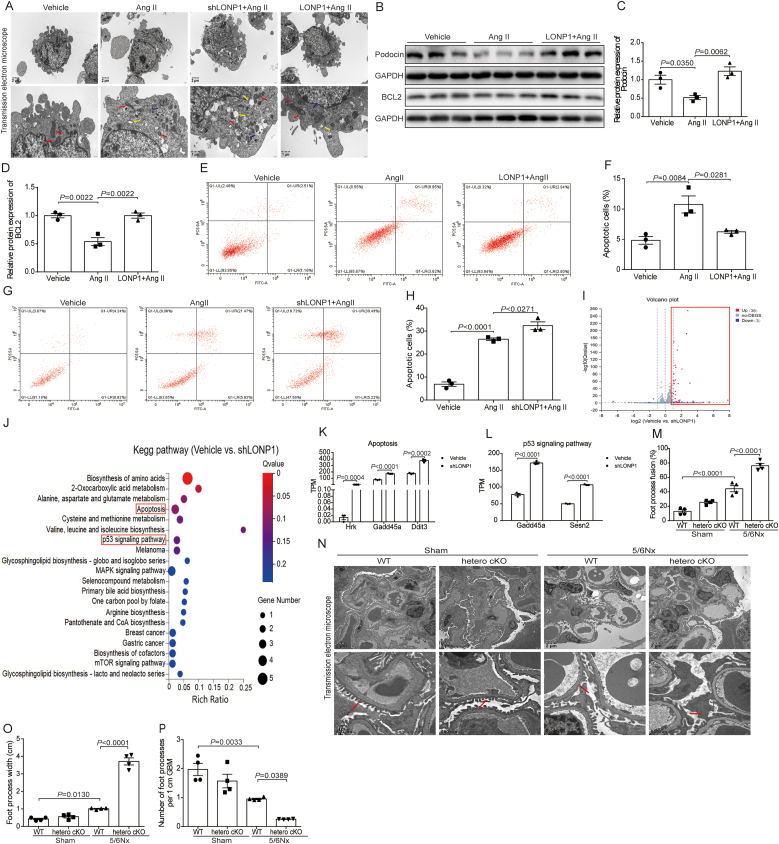
Knockdown of endothelial LONP1 facilitates Ang II-induced podocytes injury. A) Representative images of TEM on MPCs induced by culture supernatant of MAECs (*n* = 3, Scale bar, 2 μm and 1 μm). Mitochondria (red arrows), cytoplasmic vacuole (yellow arrows), and autophagosome (blue arrows). B-D) Western blot of Podocin and BCL2 in MPCs induced by culture supernatant of MAECs transfected with LONP1 plasmid and treated with Ang II. GAPDH was used as a control (*n* = 3). Dot plots represent quantitative densitometric data from Western blot. E, F) Representative images for fluorescence-activated cell sorting (FACS) analysis after annexin V and PI staining in MPCs induced by culture supernatant of MAECs transfected with LONP1 plasmid and treated with Ang II. Quantitative analysis of apoptotic cells (*n* = 3). G, H) Representative images for FACS analysis after annexin V and PI staining in MPCs induced by culture supernatant of MAECs transfected with shRNA of LONP1 and treated with Ang II. Quantitative analysis of apoptotic cells (*n* = 3). I) Volcano plot of differentially expressed genes between vehicle and shLONP1 groups (*n* = 3). J) KEGG pathway analysis of differentially expressed genes between two groups of MPCs induced by culture supernatant of MAECs transfected with shRNA of LONP1 and NC plasmid (*n* = 3). K, L) Expression of differentially expressed genes between vehicle and shLONP1 groups of MPCs in apoptosis (K) and p53 signaling pathway (L) (*n* = 3). M) Analysis of the percentage of foot process fusion in Fig. N (*n* = 4). N) Representative images of TEM on renal tissues of WT and hetero cKO mice after 5/6Nx (*n* = 4, Scale bar, 2 μm and 500 nm). O) Analysis of foot processes width in Fig. N (*n* = 4). P) Analysis of the number of foot processes per 1 cm GBM in Fig. N (*n* = 4).

To further examine the indirect effects of LONP1 intervention in MAECs on MPCs, we cultured MPCs with MAECs culture supernatant. The TEM analysis showed that the pathological changes of mitochondrial ridge swelling, cytoplasmic vacuolation and autophagy were more serious in shLONP1+Ang II group, but milder in LONP1+Ang II group, compared with the Ang II group (Fig. 7A). As shown in Fig. 7B-F, the overexpression of LONP1 in MAECs significantly inhibited the decrease of Podocin and BCL2 expression and the apoptosis of MPCs exposed to Ang II-treated MAECs culture supernatant. Conversely, knocking down LONP1 in MAECs further exacerbated the apoptosis of MPCs induced by Ang II-treated MAECs culture supernatant (Fig. 7G and H). Moreover, the RNA sequencing results revealed that 41 differentially expressed genes between vehicle and shLONP1 groups were also involved mainly in apoptosis related pathways, such as apoptosis (Hrk, Gadd45a, Ddit3) and p53 signaling pathway (Gadd45a, Sesn2) (Fig. 7I-L). Consistent with the in vitro results, foot process exfoliation and fusion occurred in the kidney tissue of WT 5/6Nx mice, whereas heterozygous knockout of endothelial cell-LONP1 further aggravated the pathological changes in the mouse kidney foot process detected by TEM analysis (Fig. 7M-P).

## 4 Discussion

Glomerulosclerosis is a final common pathological manifestation of end-stage CKD. Given the highly vascularized nature of the kidney, microvascular endothelial cells play a key role in regulating the glomerular filtration barrier (GFB) [52,53]. Damaged vascular endothelial cells act as the principal factors in initiating and mediating glomerulosclerosis [6,7]. Therefore, protecting vascular endothelial cells from damage is essential for preventing the development and progression of CKD. In this study, we found that LONP1 deficiency in endothelial cells disrupted the mitochondrial redox balance and aggravated glomerulosclerosis by protecting against SOD2 ubiquitination, while LONP1 overexpression alleviated cellular inflammatory injuries.

Mitochondria are the main site of ROS production, and the abnormal ROS release causes disruption of redox balance, mitochondrial dysfunction and cellular damage [54]. The quantity and function of mitochondrial antioxidant proteins are essential for scavenging reactive oxygen radicals and maintaining the redox balance [55]. LONP1, a major mitochondrial protease, is involved in degrading abnormal mitochondrial proteins, especially oxidative damaged and misfolded proteins [14]. Previous studies have shown that complete deletion of LONP1 in mice results in embryonic lethality [17]. Reduced expression or activity of LONP1 causes mitochondrial dysfunction, which is closely associated with various diseases [14,56]. Our previous studies showed that LONP1 expression in kidney tissues of CKD patients was significantly reduced, and its absence in podocytes caused podocytopathy, and in tubular epithelial cells led to mitochondrial dysfunction and renal fibrosis [23,24,57]. In this study, we found a significant decrease of endothelial cell LONP1 expression in glomerulus from CKD patients, compared with the controls. And in the in vitro models, the LONP1 expression was correlated negatively with the concentration of stimulus, suggesting a key role of endothelial LONP1 in glomerulosclerosis. Therefore, we demonstrated the specific role and mechanism of endothelial LONP1 in glomerulosclerosis in CKD, by using endothelial cell LONP1 cKO mice.

Strikingly, a remarkable increase in total ROS and mtROS was observed in the glomerular endothelial cells extracted from endothelial cell LONP1 cKO mice. To clarify the regulatory mechanism of LONP1 in ROS release, we detected the expression of cellular antioxidant proteins in MAECs after knocking down LONP1. We found that mitochondrial antioxidant protein SOD2 was markedly downregulated in the MAECs after knocking down LONP1, and glomerular endothelial cells extracted from LONP1 cKO mice, compared with that in the controls. By performing Co-IP, protein degradation experiment, molecular docking, and molecular interaction assays, we confirmed for the first time that LONP1 bound with SOD2 to stabilize SOD2 expression, rather than the previously extensively researched functions of LONP1 [15]. SOD2 is known to convert superoxide anion (O^2−^) into oxygen and hydrogen peroxide (H_2_O_2_), thereby preventing oxidative damage caused by free radicals and maintaining cellular homeostasis, which plays an important role in disease research [58]. Previously, the systemic loss of SOD2 caused severe damage to the heart, lungs, and nervous system of mice [59]. SOD2 played a protective role in kidney disease and could be identified as an indicator of severity and prognosis [39]. Considering that most eukaryotic proteins are degraded by the ubiquitin–proteasome pathway, we speculate that LONP1, primarily relying on its molecular chaperone function, binds to SOD2 within the matrix. This interaction, through spatial hindrance and/or induction of conformational changes, masks the ubiquitination sites on SOD2, thereby effectively preventing its recognition and tagging by intra-mitochondrial E3 ubiquitin ligases, thereby blocking the degradation of SOD2, which was confirmed by ubiquitination experiments. This ‘guardian’ role of LONP1 contrasts with its traditional protein degradation function, highlighting the diversity of its roles in maintaining mitochondrial protein homeostasis. These findings suggested that LONP1 interacted with SOD2 to counteract ROS overload and maintain redox balance by protecting against the ubiquitination-mediated degradation of SOD2.

In our study, redox imbalance induced by LONP1 deficiency in endothelial cells led to mitochondrial dysfunction and cellular inflammatory injury, and aggravated the 5/6Nx-induced kidney fibrosis mainly in glomeruli. Given that delicate balance of interactions between various cells is essential for maintaining the integrity of the glomeruli and kidney function [60,61], the cell-cell interaction was performed in this study. We found that LONP1 deficiency in endothelial cells indirectly promoted the mesangial cell proliferation and podocyte injury within the glomerulus, possibly through the abnormal release of ROS or inflammatory cytokines. Furthermore, as a critical secondary messenger, ROS not only stimulated pro-inflammatory factor secretion but also upregulated profibrotic mediators such as TGF-β and ET-1 [[62], [63], [64]]. These pathological changes thus reflect a ROS-orchestrated synergistic ‘cocktail’ effect. Future studies will quantify these factors and dissect their contributions using specific inhibitors. Notably, SOD2 supplementation (application of SOD2 mimetics or exogenous transfection of SOD2 plasmid) effectively alleviated endothelial injury and renal fibrosis exacerbated by LONP1 deficiency. Building on our previous finding that podocyte-specific LONP1 knockout induces podocyte injury and glomerulopathy [23], we propose that MnTBAP protects against podocyte damage through a dual mechanism: direct antioxidant defense in LONP1-reduced podocytes, and indirect protection of endothelial function to maintain a healthy glomerular environment. Collectively, the rescue experiment further confirmed that LONP1 deficiency exerts adverse effects by disrupting SOD2 stability. Though our findings demonstrate that SOD2 mimics effectively attenuate oxidative stress resulting from LONP1 deficiency, a contributory role of other antioxidant enzymes, such as catalase and GPX, cannot be entirely excluded, and their involvement likely represents a secondary adaptive response within the antioxidant defense system.

This study has several limitations and unanswered questions. First, we used endothelial cell-specific LONP1 knockout mice to determine the adverse role of LONP1 deficiency in the development and progression of glomerulosclerosis. However, further studies are needed to fully understand the protective role of LONP1 in glomerulosclerosis using endothelial cell-specific LONP1 knock-in mice. Second, although our study establishes that LONP1 stabilizes SOD2 by protecting against its ubiquitination, the precise molecular details remain to be elucidated. Specifically, the exact ubiquitination sites on SOD2 that are shielded by LONP1. Meanwhile, future work is needed to identify the specific mitochondrial E3 ligase for SOD2 in this experimental setting. Third, the generalizability of our findings is limited by the pediatric-specific origin of our human samples and models, which may not fully represent the pathogenesis of adult-onset CKD predominantly driven by diabetes, hypertension, or vascular disease. We will initiate collaborations with adult nephrology centers to address this gap in future work. Finally, the use of young adult mice limits direct translation to age-related human CKD, as the model does not capture aging-specific processes such as cumulative oxidative stress and inflammaging. The role of LONP1 in aging-related kidney pathology requires further validation in elderly models.

Together, our work reveals the role of LONP1 in mitochondrial redox balance and cellular homeostasis. We suggest that endothelial LONP1, which stabilizes the SOD2 protein by protecting against SOD2 ubiquitination, exerts a protective effect on glomerulosclerosis induced by 5/6Nx and endothelial cellular injury models. Targeting the endothelial LONP1-mediated redox balance may provide a novel therapeutic opportunity to prevent glomerulosclerosis and regulate intercellular communication within the glomeruli during the CKD process.

Furthermore, this study observed downregulation of LONP1 in glomerular endothelial cells, podocytes, and tubular epithelial cells, indicating its broad involvement in the 5/6Nx-CKD model. Notably, we revealed for the first time that endothelial LONP1 exerts its protective role through a unique SOD2-redox balance axis, a mechanism distinct from its action in other renal cell types in CKD. This discovery not only demonstrates the cell-specific nature of LONP1 function but also provides new perspectives for understanding multicellular collaborative injury mechanisms under different pathological contexts. Elucidating the commonalities and differences in LONP1 functions across various renal cells will be an important direction for future research.

## Disclosure statement

All the authors declared no competing interests.

## CRediT authorship contribution statement

**Xiaolu Zhang:** Investigation, Methodology, Software, Writing – original draft. **Shuzhen Li:** Funding acquisition, Methodology, Visualization, Writing – review & editing. **Shanshan Li:** Data curation, Validation. **Bing Liu:** Methodology, Visualization. **Guixia Ding:** Funding acquisition, Validation. **Mengqiu Wu:** Validation, Visualization. **Yue Zhang:** Funding acquisition, Investigation, Validation. **Songming Huang:** Investigation, Supervision, Validation. **Wei Gong:** Conceptualization, Writing – review & editing. **Zhanjun Jia:** Conceptualization, Writing – review & editing. **Aihua Zhang:** Conceptualization, Funding acquisition, Writing – review & editing.

## Declaration of competing interest

The authors declare that they have no known competing financial interests or personal relationships that could have appeared to influence the work reported in this paper.

## Data Availability

Data will be made available on request.
