# Supplementary material for: Endothelial Lon protease 1 facilitates the redox balance to prevent glomerulosclerosis by acting on superoxide dismutase 2 ubiquitination
Source: Redox Biol. 2025 Nov 19;88:103929. doi: 10.1016/j.redox.2025.103929 (PMC12681905; doi:10.1016/j.redox.2025.103929)
Supplement: Multimedia component 1 [file mmc1.docx]

**Data Supplement to**:

**Title:** **Endothelial Lon protease 1 facilitates the redox balance to prevent glomerulosclerosis by acting on superoxide dismutase 2** **ubiquitination**

Xiaolu Zhang^a,b,c,e†^, Shuzhen Li^a,b,c†#^, Shanshan Li^a,b,c^, Bing Liu^a,b,c^, Guixia Ding^a,b,c^, Mengqiu Wu^a,b,c^, Yue Zhang^a,b,c^, Songming Huang^a,b,c^, Wei Gong^a,b,c#^, Zhanjun Jia^a,b,c#^, Aihua Zhang^a,b,c,d#^

^a^ Department of Nephrology, Children’s Hospital of Nanjing Medical University, Nanjing 210008, China.

^b^ Nanjing Key Lab of Pediatrics, Children’s Hospital of Nanjing Medical University, Nanjing 210008, China.

^c^ Jiangsu Key Laboratory of Early Development and Chronic Diseases Prevention in Children, Nanjing Medical University, Nanjing, 210029, China.

^d^ State Key Laboratory of Reproductive Medicine and Offspring Health, Nanjing Medical University, Nanjing 211166, China.

^e^ Department of Child Health Care, Children’s Hospital of Nanjing Medical University, Nanjing 210008, China.


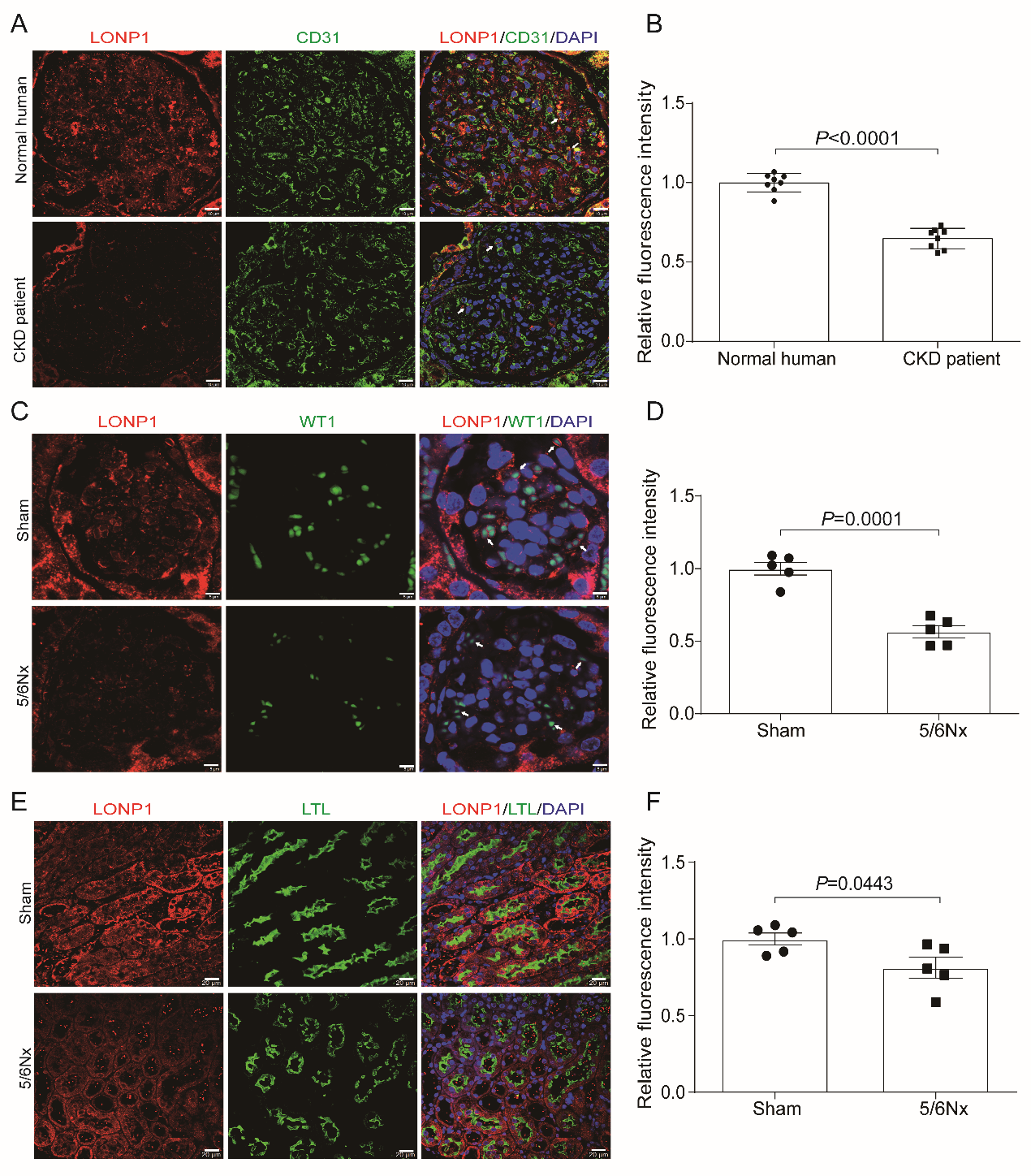


**Supplementary Fig. 1. LONP1 expression changes in CKD patient kidney samples, podocytes and tubular epithelial cells of the 5/6Nx models.** A) Immunofluorescence (IF) staining of LONP1 and CD31 in the normal human and CKD patient kidneys. LONP1 (red), CD31 (green), DAPI (blue), and co-localized position (white arrows) (*n* = 8, Scale bar, 10 μm). B) Relative fluorescence intensity statistics of IF staining of LONP1 (*n* = 8). C) IF staining of LONP1 and WT1 in the sham and 5/6Nx mice kidneys. LONP1 (red), WT1 (green), DAPI (blue), and co-localized position (white arrows) (*n* = 5, Scale bar, 5 μm). D) Relative fluorescence intensity statistics of IF staining of LONP1 (*n* = 5). E) IF staining of LONP1 and LTL in the sham and 5/6Nx mice kidneys. LONP1 (red), LTL (green), and DAPI (blue) (*n* = 5, Scale bar, 20 μm). F) Relative fluorescence intensity statistics of IF staining of LONP1 (*n* = 5).


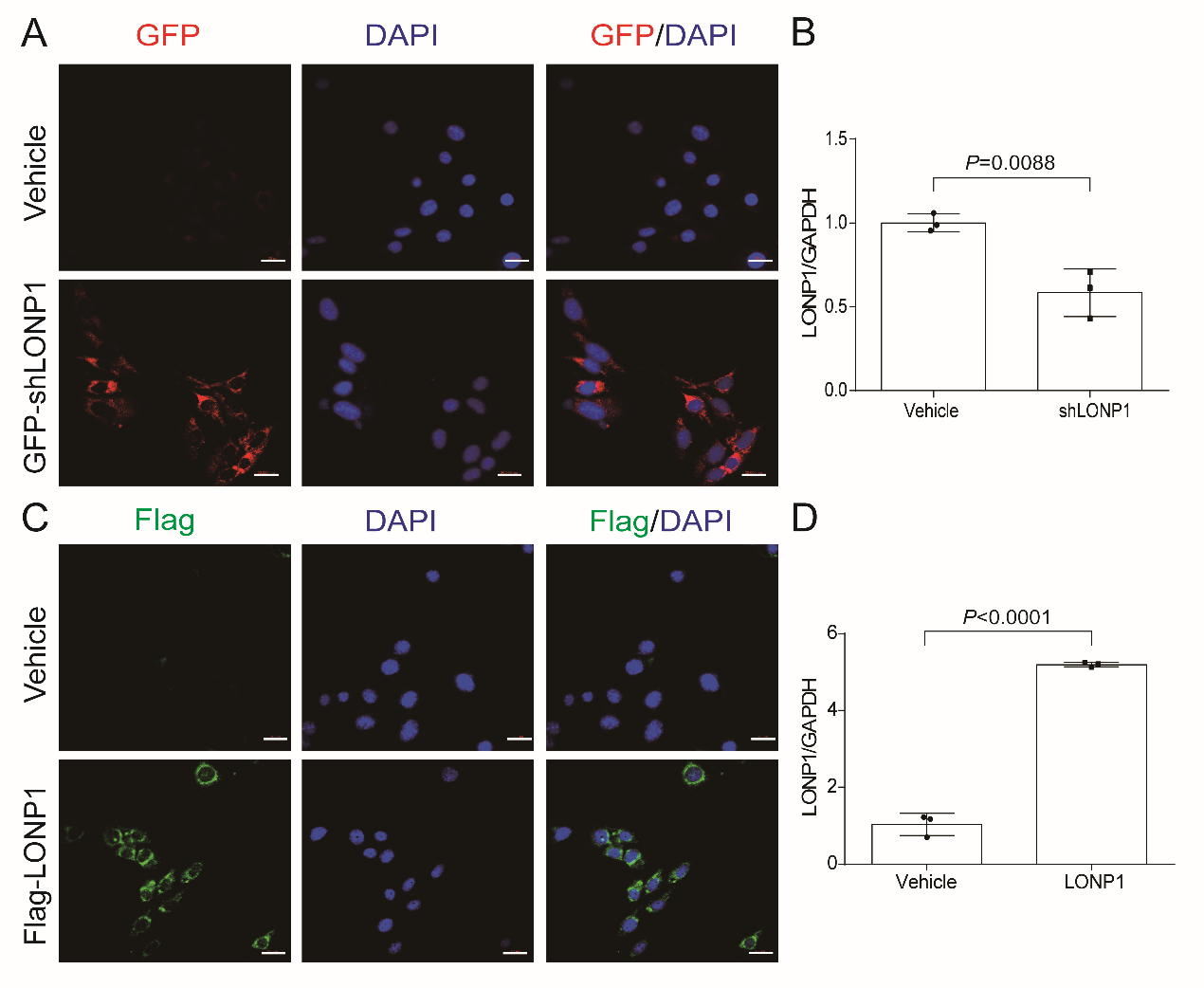


**Supplementary Fig. 2. The transfection efficiency of GFP-tagged shRNA targeting LONP1 and Flag-tagged LONP1 overexpression plasmid in MAECs.** A) IF staining of GFP in MAECs transfected with shRNA targeting LONP1 (*n* = 3, Scale bar, 20 μm). B) qPCR analysis of LONP1 in the MAECs transfected with shRNA targeting LONP1 (*n* = 3). C) IF staining of Flag in MAECs transfected with LONP1 overexpression plasmid (*n* = 3, Scale bar, 20 μm). D) qPCR analysis of LONP1 in the MAECs transfected with LONP1 overexpression plasmid (*n* = 3).

**
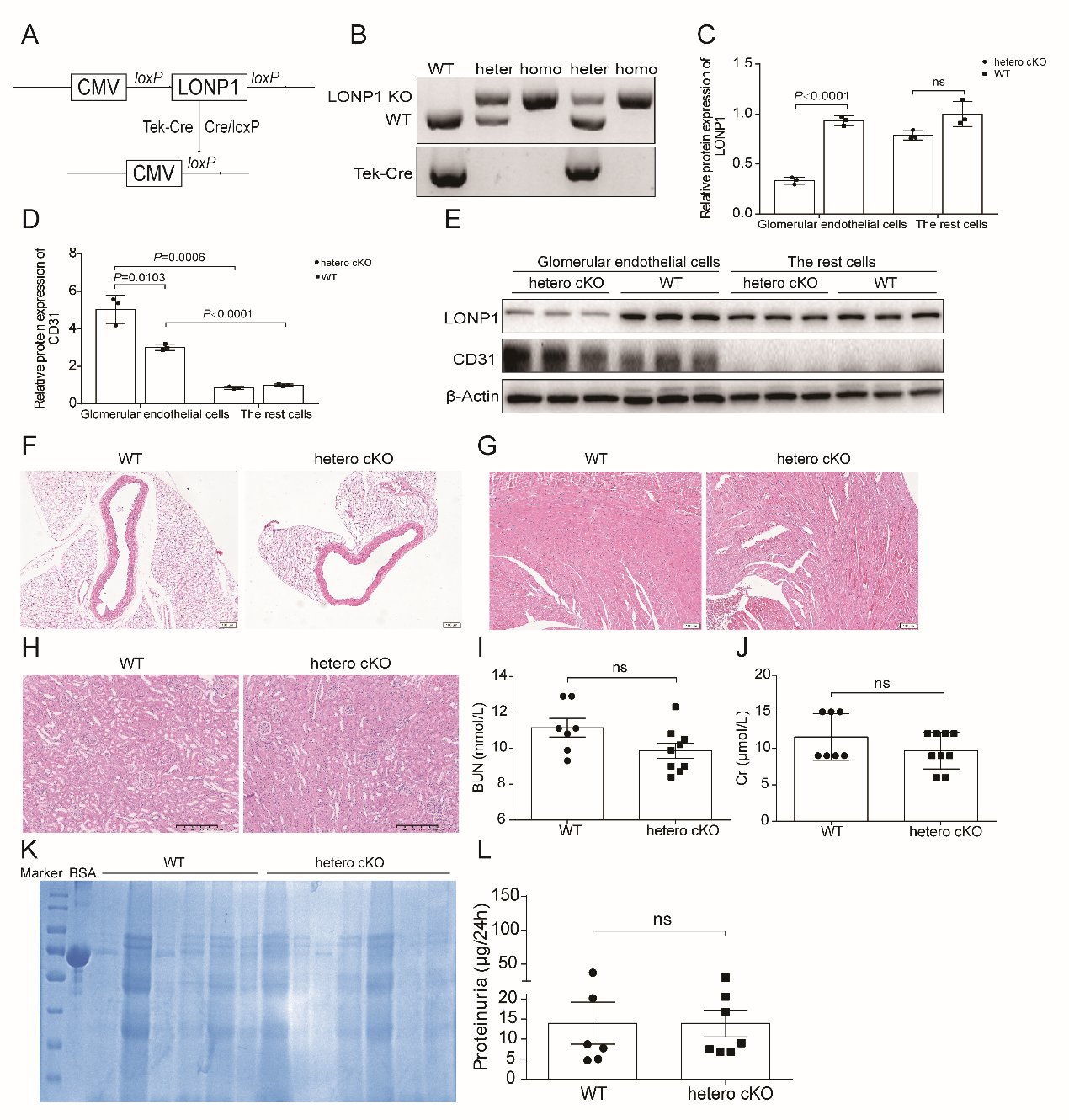
**

**Supplementary Fig. 3. Endothelial cell LONP1 heterozygous knockout exerts no effect on mouse development and renal function.** A) Schematic diagram of the LONP1^fl/+^ mouse construction strategy. B) PCR detection of gene type of wild-type (WT), heterozygous (heter), and homozygous (homo) knockout mice. C-E) Western blot of LONP1 and CD31 expression in the glomerular endothelial cells extracted from WT and hetero cKO mice (E). β-Actin was used as a control (*n* = 3). Dot plots represent quantitative densitometric data of LONP1(C) and CD31(D) from western blot. F) HE staining of aorta in WT and hetero cKO mice. Scale bar, 100 μm. G) HE staining of heart in WT and hetero cKO mice. Scale bar, 100 μm. H) HE staining of kidney in WT and hetero cKO mice. Scale bar, 200 μm. I, J) Analysis of blood urea nitrogen (BUN) (I) and serum creatinine (Cr) (J) in WT and hetero cKO mice (*n* = 7-9). K) Coomassie blue staining of representative urine samples by sodium dodecyl sulfate polyacrylamide gel electrophoresis in WT and hetero cKO mice (*n* = 6-7). L) ELISA of urinary microalbumin in WT and hetero cKO mice (*n* = 6-7).


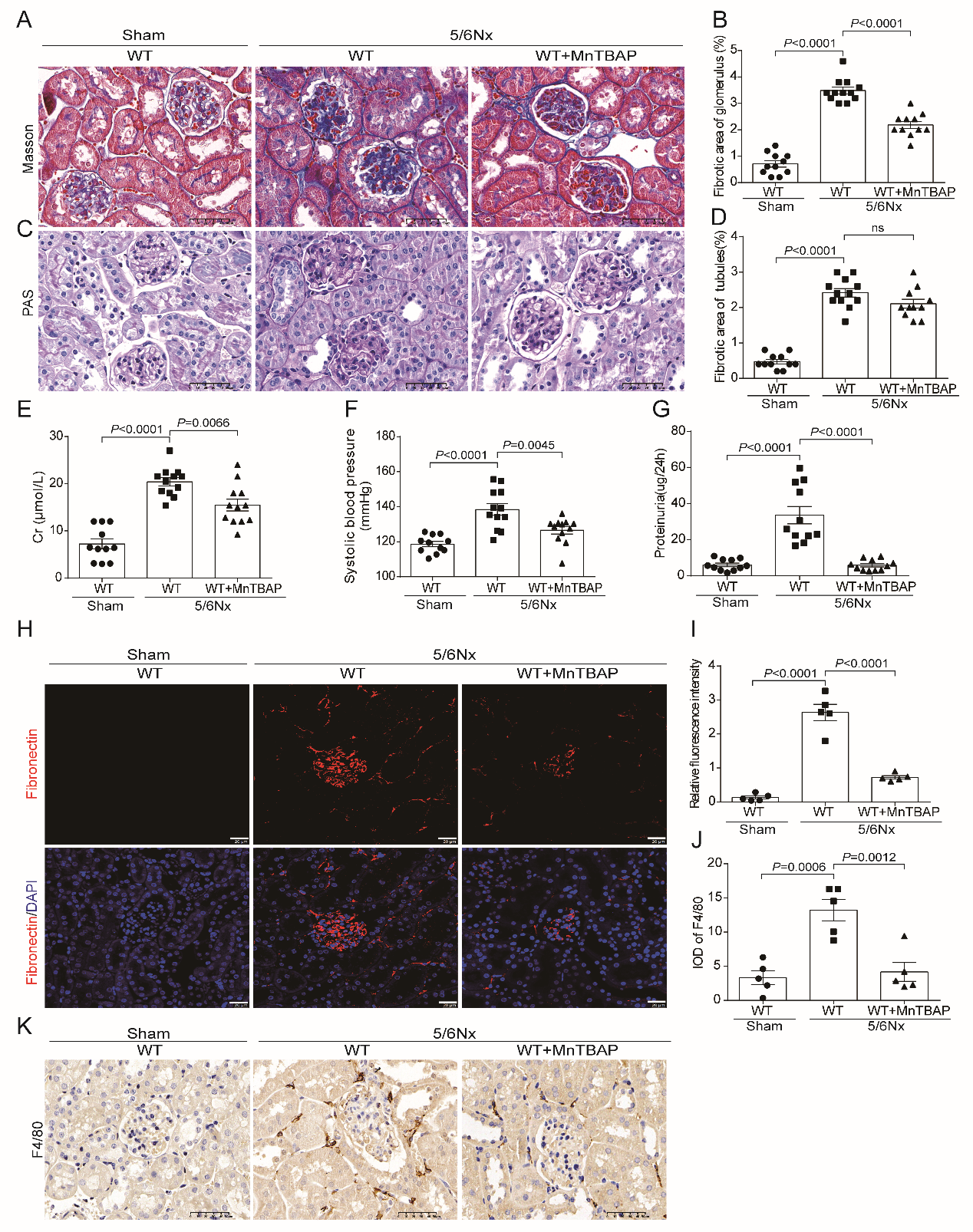


**Supplementary Fig. 4. SOD2 supplementation alleviates 5/6Nx-induced glomerulosclerosis in the wild type mouse.** A) Masson’s trichrome staining in different groups (*n* = 11-12, Scale bar, 50 μm). B, D) Fibrotic area of glomerulus (B) and tubules (D) statistics of Masson’s trichrome staining in different groups (*n* = 11-12). C) PAS staining in different groups (*n* = 11-12, Scale bar, 50 μm). E) Analysis of Cr in different groups (*n* = 11-12). F) Systolic blood pressure in different groups detected by tail-cuff (*n* = 11-12). G) ELISA of urinary microalbumin in different groups (*n* = 11). H) IF staining of Fibronectin in different groups (*n* = 5, Scale bar, 20 μm). I) Relative fluorescence intensity statistics of IF staining of Fibronectin (*n* = 5). J) Immunohistochemical (IHC) semi-quantitative IOD analysis of F4/80 (*n* = 5). K) IHC staining of F4/80 in different groups (*n* = 5, Scale bar, 50 μm). IOD, Integral Optical Density.


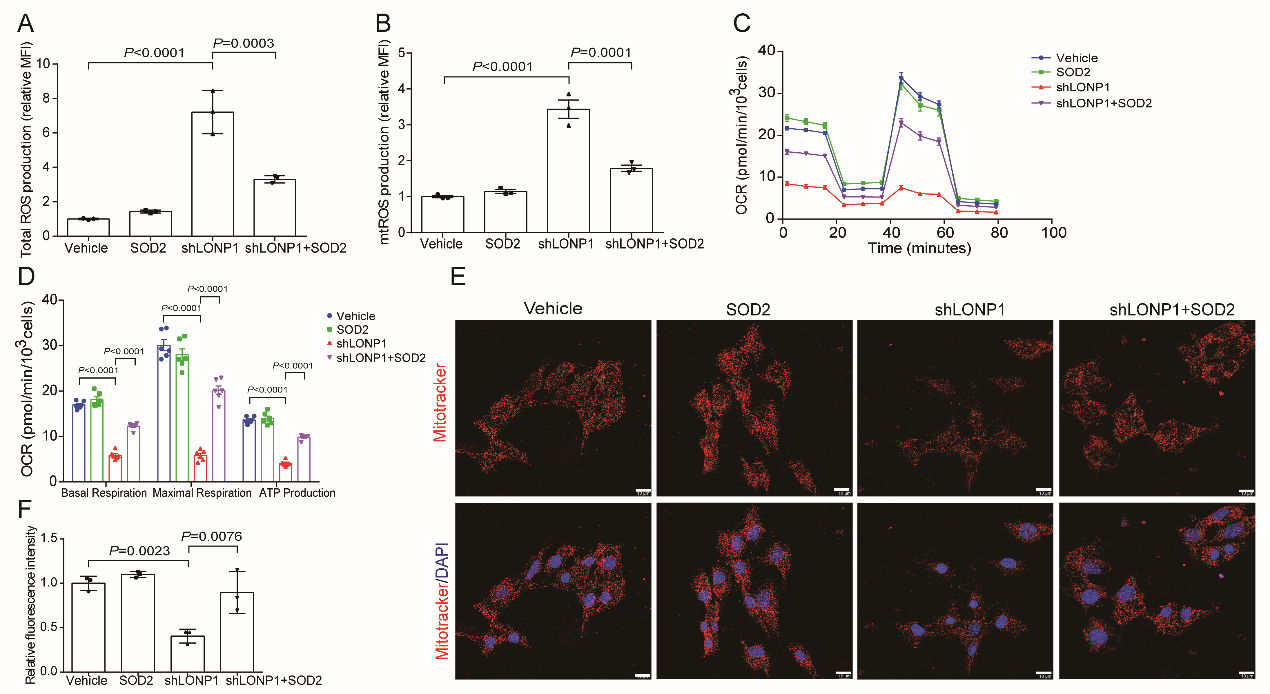


**Supplementary Fig. 5. The effects of SOD2 overexpression on ROS and mitochondrial defects caused by LONP1 knockdown.** A, B) Quantification of the mean fluorescence intensity (MFI) of DCF (indicating total reactive oxygen species [ROS]; *n* = 3) and mitoSOX (indicating mitochondrial ROS [mtROS]; *n* = 3) using flow cytometry in MAECs co-transfected with shLONP1 and SOD2. C) Oxygen consumption rate (OCR) of MAECs co-transfected with shLONP1 and SOD2 (*n* = 6). D) Quantification of basal respiratory, maximal respiratory, and ATP production in MAECs co-transfected with shLONP1 and SOD2 (*n* = 6). E) IF staining of MitoTracker in MAECs co-transfected with shLONP1 and SOD2 (*n* = 3, Scale bar, 20 μm). F) Relative fluorescence intensity of MitoTracker in MAECs co-transfected with shLONP1 and SOD2 (*n* = 3).

**Table S1.** The clinical characteristics and pathologic diagnosis of renal biopsy

| Number | Gender(M/F) | Age(years) | Pathologic diagnosis |
| --- | --- | --- | --- |
| 1 | M | 10.00 | Focal segmental glomerulosclerosis |
| 2 | M | 5.42 | Focal segmental glomerulosclerosis |
| 3 | F | 10.58 | Focal segmental glomerulosclerosis |
| 4 | M | 4.08 | Focal segmental glomerulosclerosis |
| 5 | M | 7.17 | IgA nephropathy |
| 6 | M | 8.92 | Mesangial proliferative glomerulonephritis |
| 7 | M | 4.42 | Focal segmental glomerulosclerosis |
| 8 | F | 3.42 | Focal segmental glomerulosclerosis |
| 9 | F | 15.83 | IgA nephropathy |
| 10 | F | 16.25 | Focal segmental glomerulosclerosis |
| 11 | F | 6.75 | Henoch-Schönlein Purpura Nephritis |

M, Male; F, Female

**Table S2.** Details of antibodies used for western blot, immunohistochemical staining, and immunofluorescence images

| Antibody | Vendors | Catalog Number |
| --- | --- | --- |
| LONP1 | Proteintech (Rosemont, IL, USA) | 15440-1-AP |
| LONP1 | Proteintech (Rosemont, IL, USA) | 66043-1-Ig |
| SOD2 | Proteintech (Rosemont, IL, USA) | 24127-1-AP |
| SOD2 | Proteintech (Rosemont, IL, USA) | 66474-1-Ig |
| Fibronectin | Abcam (Cambridge, MA, USA) | ab2413 |
| CD31 | Cell Signaling Technology (Beverly, MA, USA) | 77699 |
| CD31 | Proteintech (Rosemont, IL, USA) | 66065-2-Ig |
| SOD1 | Proteintech (Rosemont, IL, USA) | 10269-1-AP |
| SOD3 | Proteintech (Rosemont, IL, USA) | 14316-1-AP |
| SOD3 | Cohesion Biosciences (Suzhou, China) | CQA6009 |
| CyclinD1 | Proteintech (Rosemont, IL, USA) | 60186-1-Ig |
| CyclinA2 | Proteintech (Rosemont, IL, USA) | 18202-1-AP |
| GAPDH | Proteintech (Rosemont, IL, USA) | 10494-1-AP |
| β-Actin | Proteintech (Rosemont, IL, USA) | 20536-1-AP |
| Flag-Tag | Sigma‒Aldrich (St. Louis, MO, USA) | F1804 |
| HA-Tag | Signaling Technology (Beverly, MA, USA) | 3724 |
| Myc-Tag | Signaling Technology (Beverly, MA, USA) | 2276 |
| F4/80 | Servicebio (Wuhan, Hubei, China) | GB113373-100 |
| VCAM-1 | ABclonal (Wuhan, Hubei, China) | A0279 |
| ICAM-1 | ABclonal (Wuhan, Hubei, China) | A5597 |
| ICAM-1 | ABclonal (Wuhan, Hubei, China) | A26412PM |
| WT1 | Santa Cruz Biotechnology (Dallas, TX, USA) | Sc-7385 |
| BCL2 | Proteintech (Rosemont, IL, USA) | 26593-1-AP |
| Podocin | ABclonal (Wuhan, Hubei, China) | A17337 |

**Table S3.** qRT-PCR primer sequence

| Gene | Primer sequence（5’-3’） |
| --- | --- |
| Mouse mtDNA (ND1) | F: ATCCTCCCAGGATTTGGAAT  R: ACCGGTAGGAATTGCGATAA |
| Mouse mtND1 | F: ACACTTATTACAACCCAAGAACACAT  R: TCATATTATGGCTATGGGTCAGG |
| Mouse mtND2 | F: CCATCAACTCAATCTCACTTCTATG  R: GAATCCTGTTAGTGGTGGAAGG |
| Mouse mtND3 | F: CCCCAAATAAATCTGTA  R: CTCATGGTAGTGGAAGT |
| Mouse mtND4 | F: GCTTACGCCAAACAGAT  R: TAGGCAGAATAGGAGTGAT |
| Mouse mtND4L | F: GCCATCTACCTTCTTCA  R: TAGGGCTAGTCCTACAGC |
| Mouse mtND5 | F: GCCAACAACATATTTCAACTTTTC  R: ACCATCATCCAATTAGTAGAAAGGA |
| Mouse mtND6 | F: GGGAGATTGGTTGATGTA  R: ATACCCGCAAACAAAGAT |
| Mouse mtCO3 | F: CGTGAAGGAACCTACCAAGG  R: ATTCCTGTTGGAGGTCAGCA |
| Mouse LONP1 | F: gtcttcactaccatggagaagg  R: tcatggatgaccttggccag |
| Mouse GAPDH | F: GTCTTCACTACCATGGAGAAGG  R: TCATGGATGACCTTGGCCAG |
| Mouse 18S | F: ACGGACAGGATTGACAGA  R: CGCTCCACCAACTAAGAA |
