# Supplementary material for: Endothelial Lon protease 1 facilitates the redox balance to prevent glomerulosclerosis by acting on superoxide dismutase 2 ubiquitination
Source: Redox Biol. 2025 Nov 19;88:103929. doi: 10.1016/j.redox.2025.103929 (PMC12681905; doi:10.1016/j.redox.2025.103929)
Supplement: Multimedia component 2 [file mmc2.pdf]

Reporting checklist for study using laboratory animals.

We complete this checklist based on the ARRIVE2.0 guidelines.by entering the page numbers from our manuscript where readers will find each of the items listed below. We write "n/a" and provide a short explanation for item does not apply. We have uploaded the completed checklist as an extra file when we submit to the journal.

	ARRIVE Essential 10	
	Item	Page Number
Study design	1	Page 5
Sample size	2	Page 5，Page 32-34
Inclusion and exclusioncriteria	3	Page 5
Randomisation	4	Page 5
Blinding	5	Page 6
Outcome measures	6	Page 5-9
Statistical methods	7	Page 12
Experimental animals	8	Page 4-5
Experimental procedures	9	Page 5
Results	10	Page 12-17

	Recommended Set	
	Item	Page Number
Abstract	11	Page 1, Page 4-5
Background	12	Page 2, Page 4-5
Objectives	13	Page 1
Ethical statement	14	Page 5
Housing and husbandry	15	Page 4-5
Animal care and monitoring	16	Page 4-5, b. any animals or data were not included in the analysis due to the high surgical trauma, and these individuals died from postoperative infections before sample collection.
Interpretation/scientificimplications	17	Page 18-22
Generalisability/translation	18	Page 18-22
Protocol registration	19	Page 5
Data access	20	Page 22
Declaration of interests	21	Page 22
