# Supplementary material for: Endothelial Lon protease 1 facilitates the redox balance to prevent glomerulosclerosis by acting on superoxide dismutase 2 ubiquitination
Source: Redox Biol. 2025 Nov 19;88:103929. doi: 10.1016/j.redox.2025.103929 (PMC12681905; doi:10.1016/j.redox.2025.103929)

Endothelial Lon protease 1 facilitates the redox balance to prevent glomerulosclerosis by acting on superoxide dismutase 2 ubiquitination

Full images of western blot

Fig1G MAECs treated with Ang II

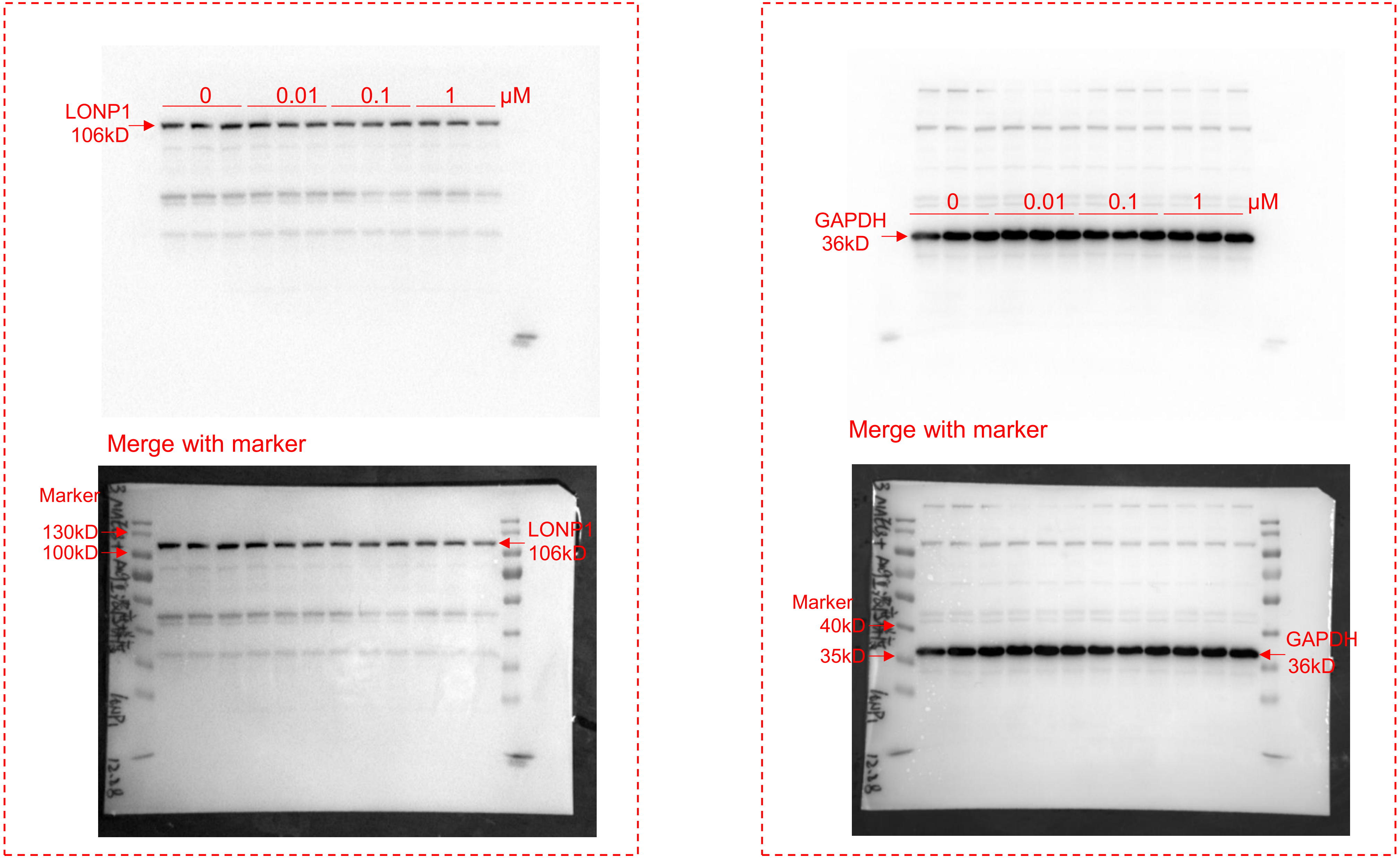

Fig1H MAECs treated with ALD

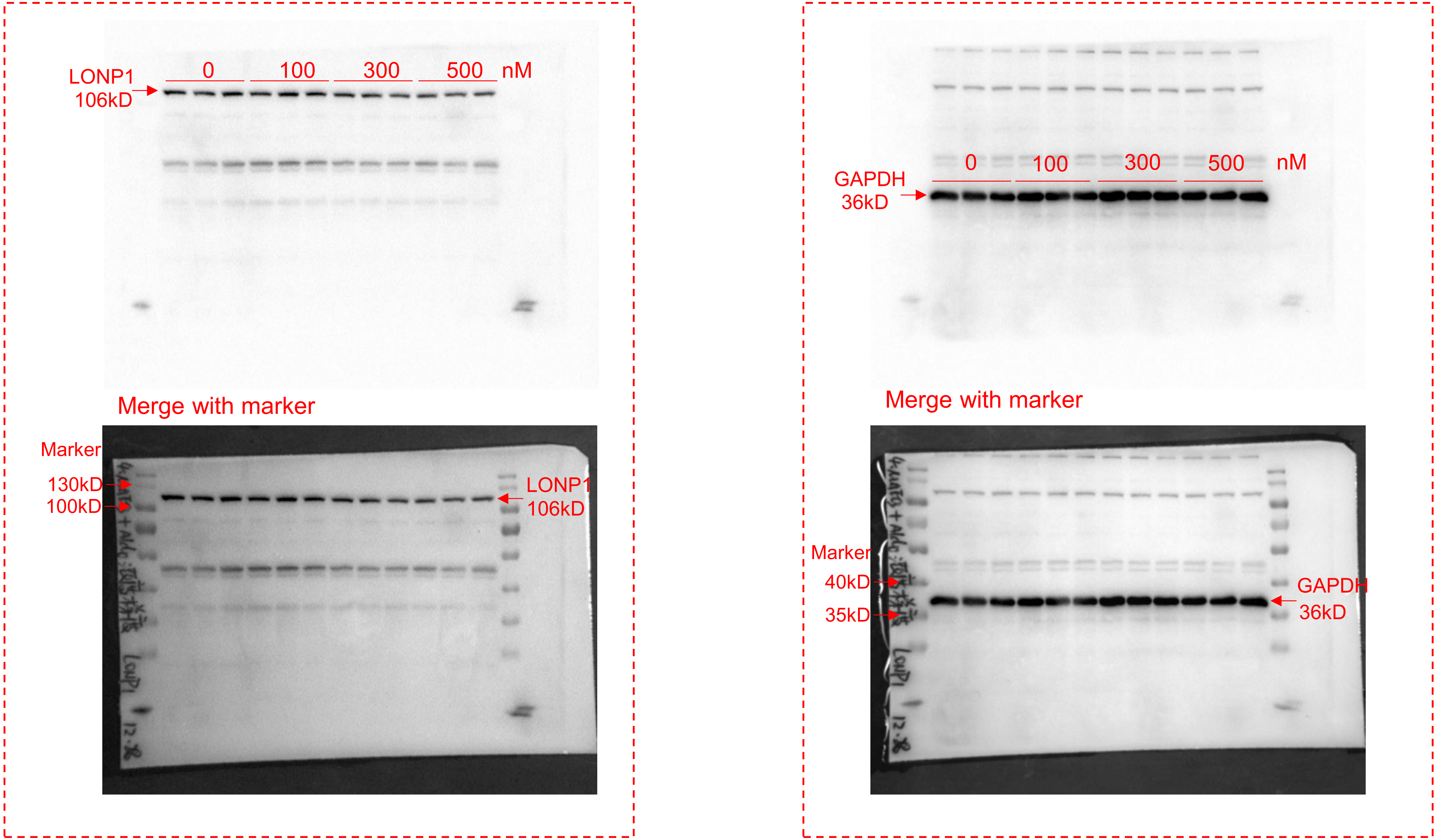

Fig1K MAECs treated with H_2O_2

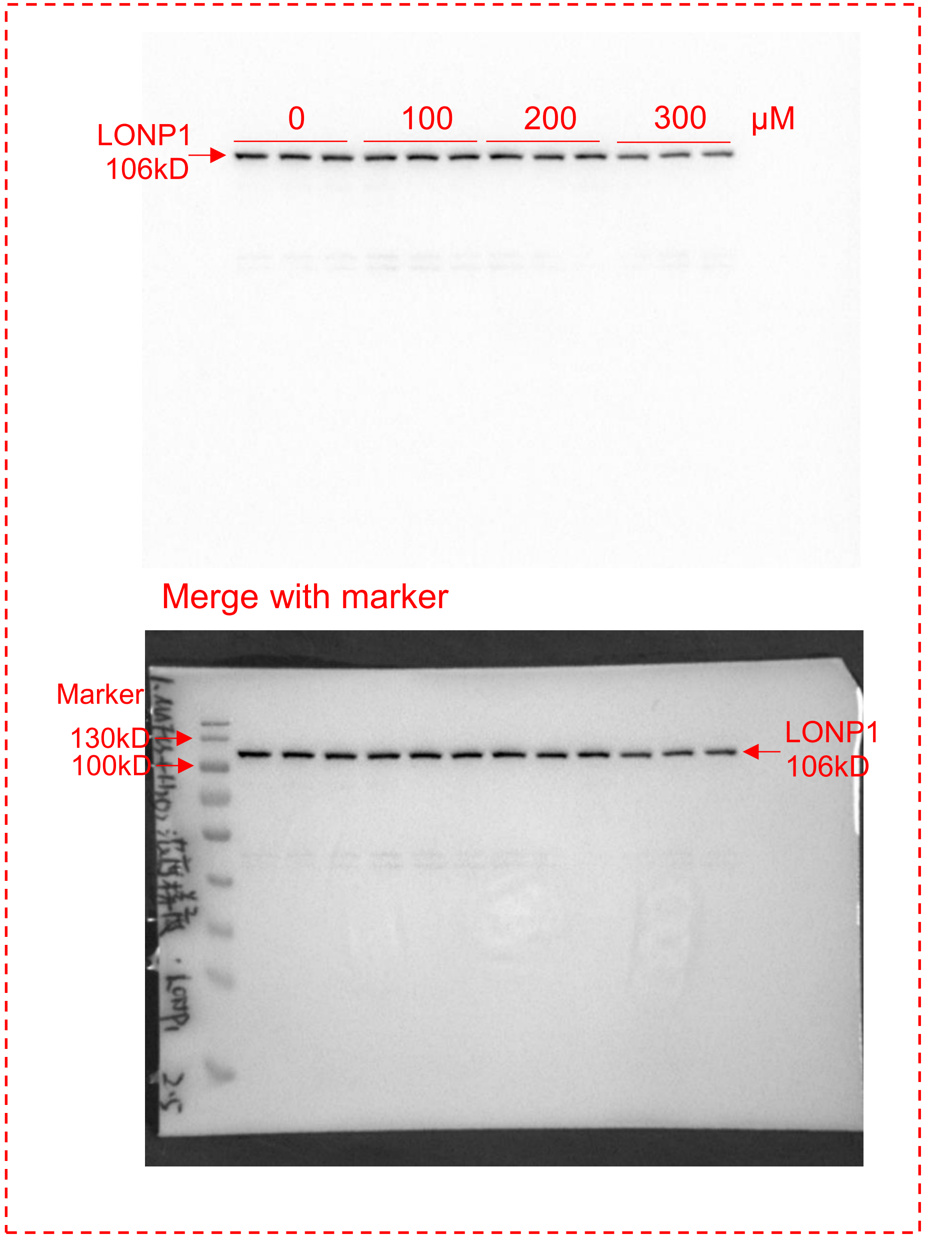

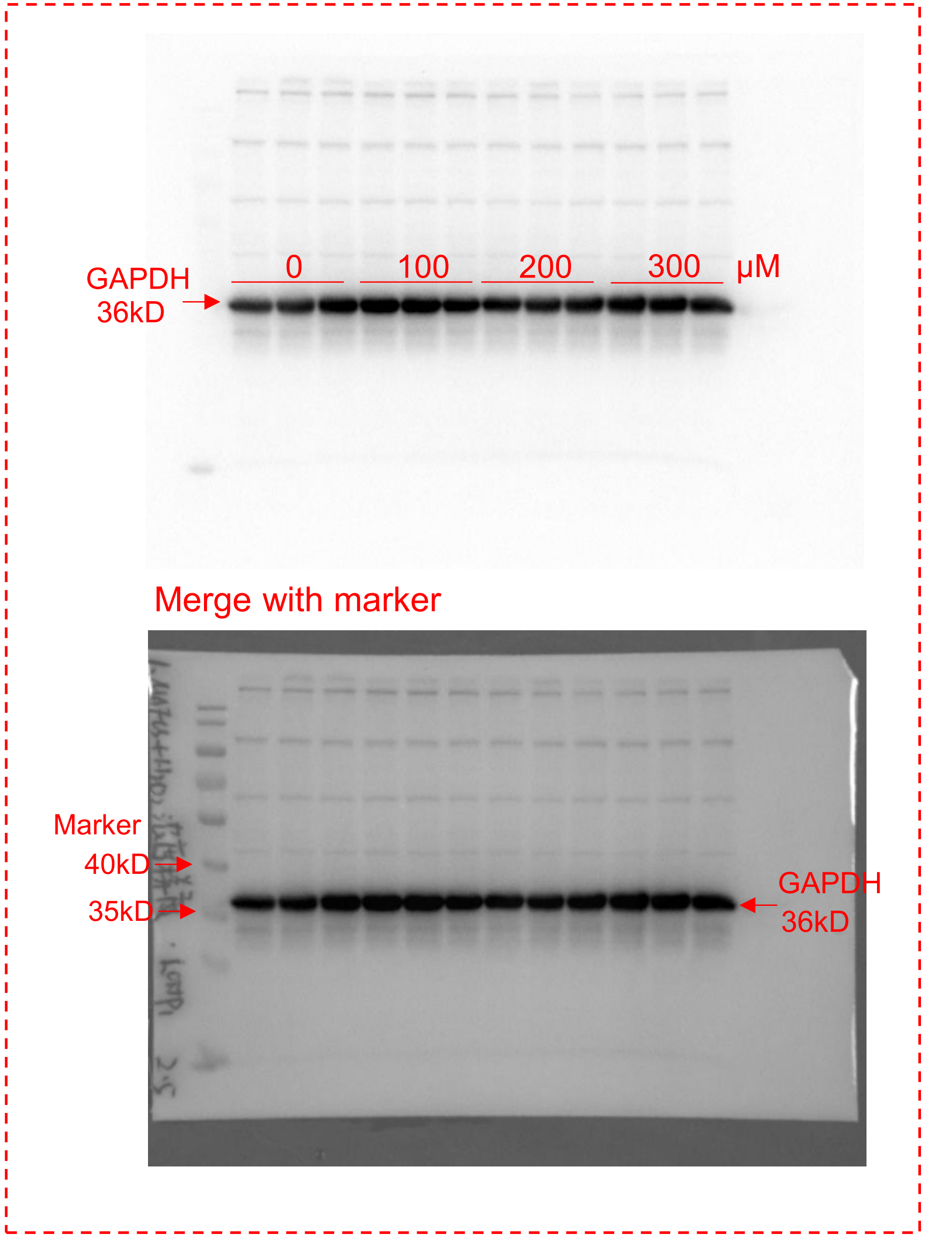

Fig2A Primary HAECs transfected with shLONP1 plasmid

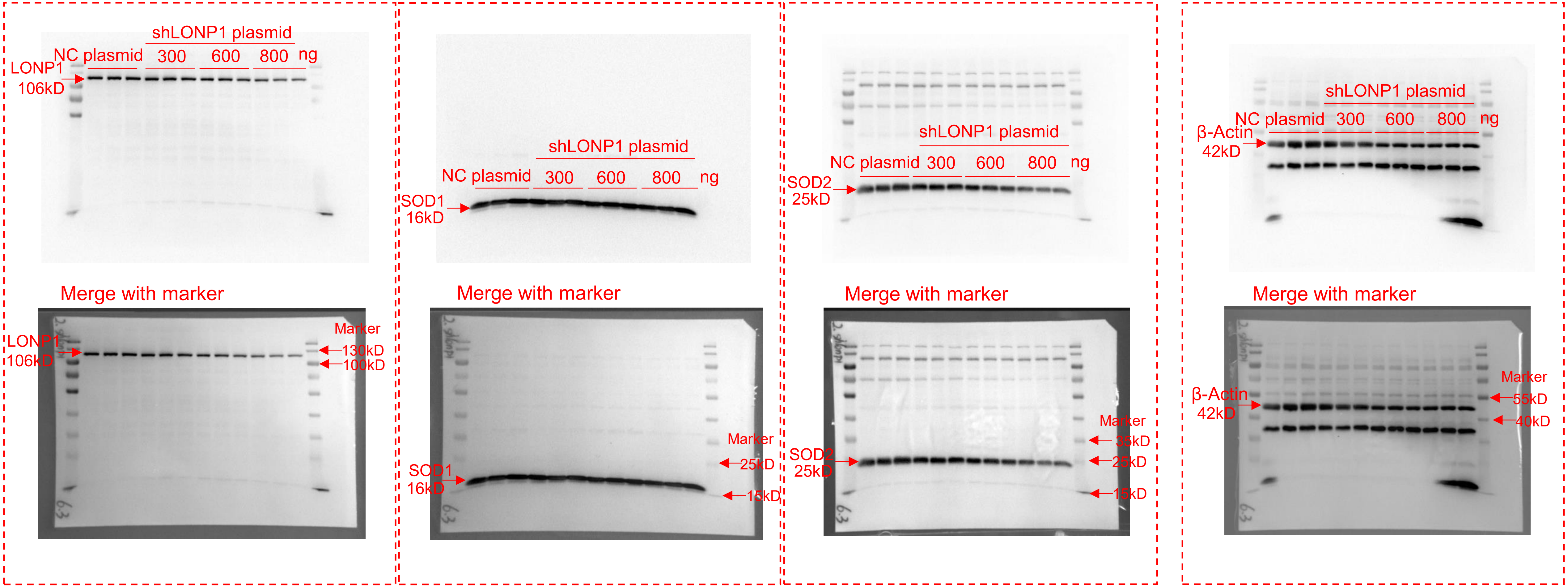

Fig2A Primary HAECs transfected with shLONP1 plasmid

Antibody of Cohesion Biosciences (CQA6009)

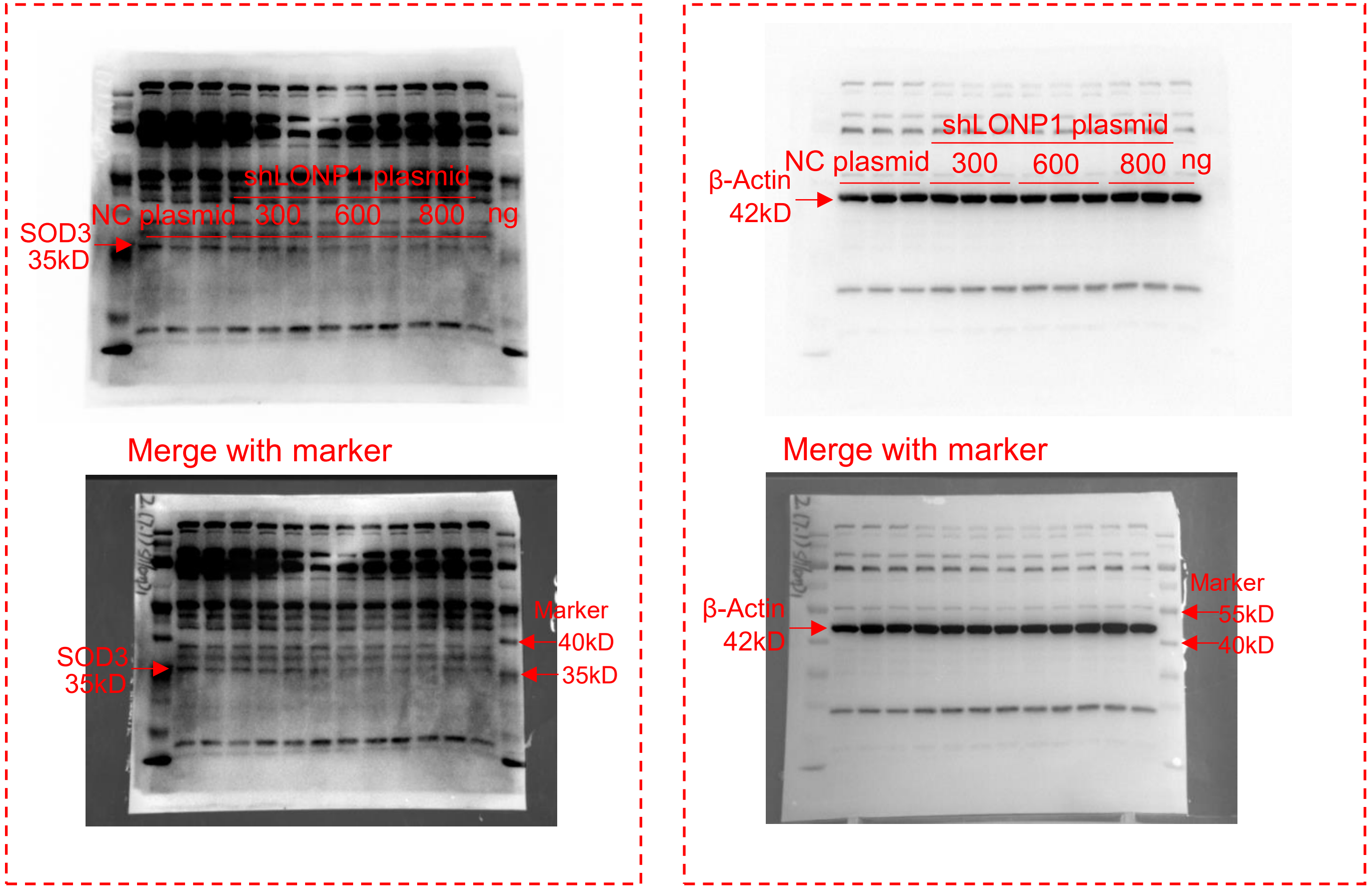

Fig2C MAECs transfected with shLONP1 plasmid

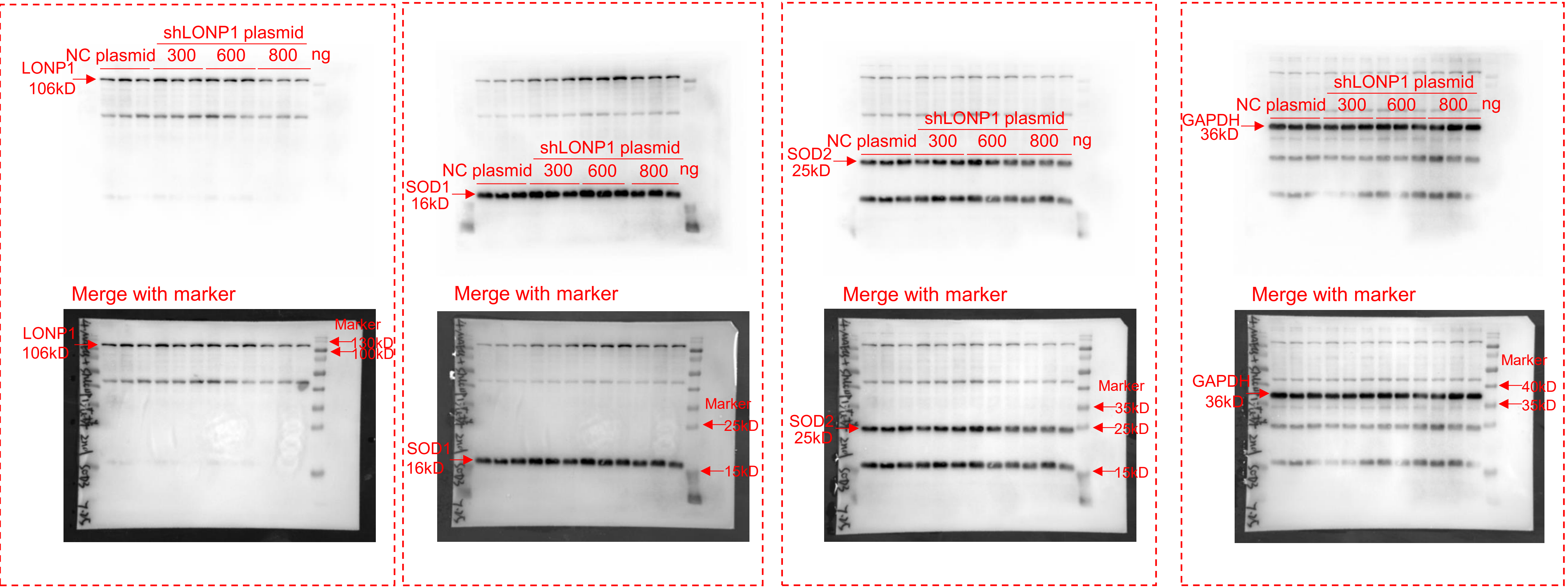

Fig2C MAECs transfected with shLONP1 plasmid

Antibody of Proteintech (14316-1-AP)

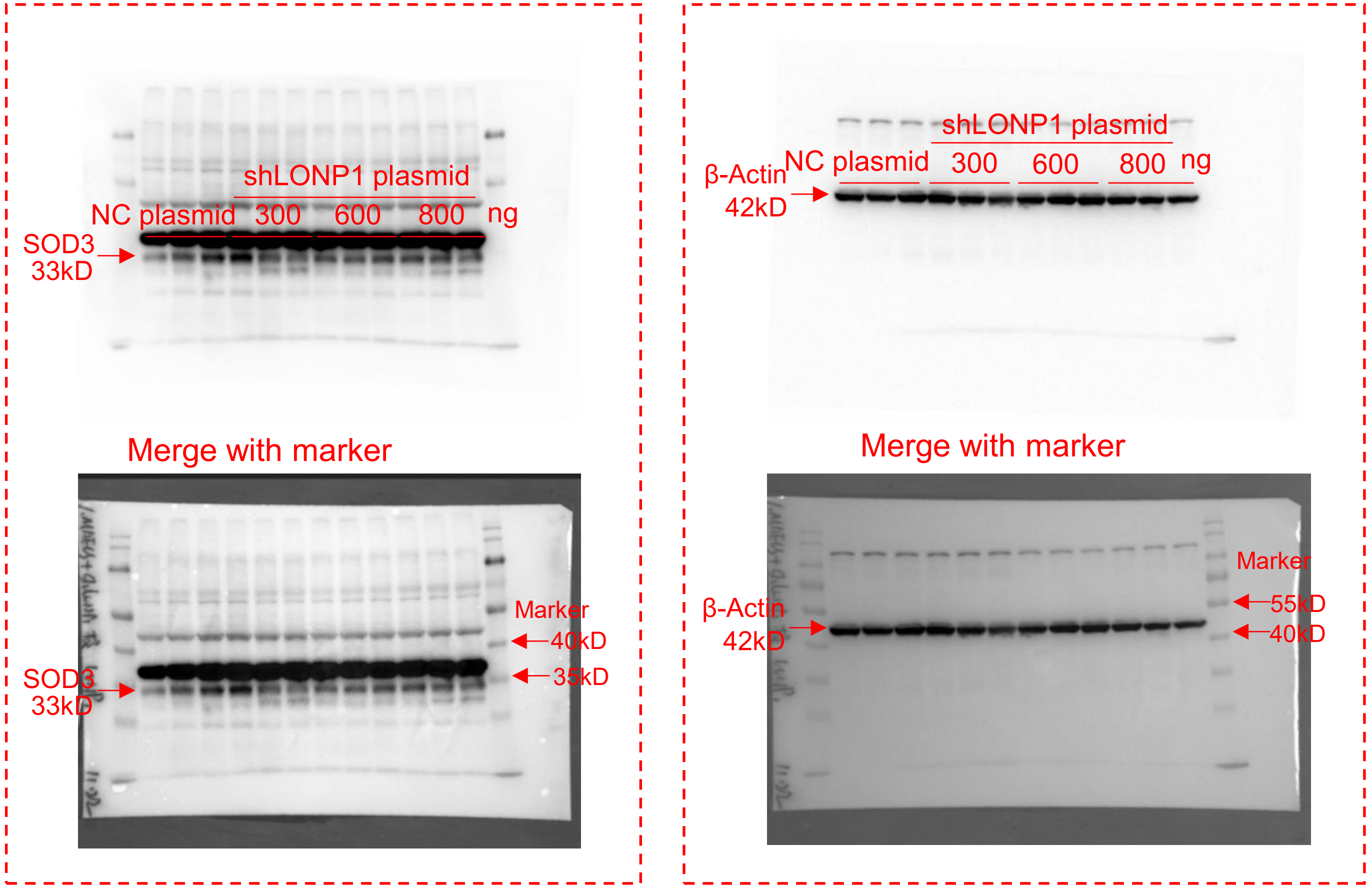

Fig2E Glomerular endothelial cells and the rest cells of LONP1 hetero cKO and WT mouse

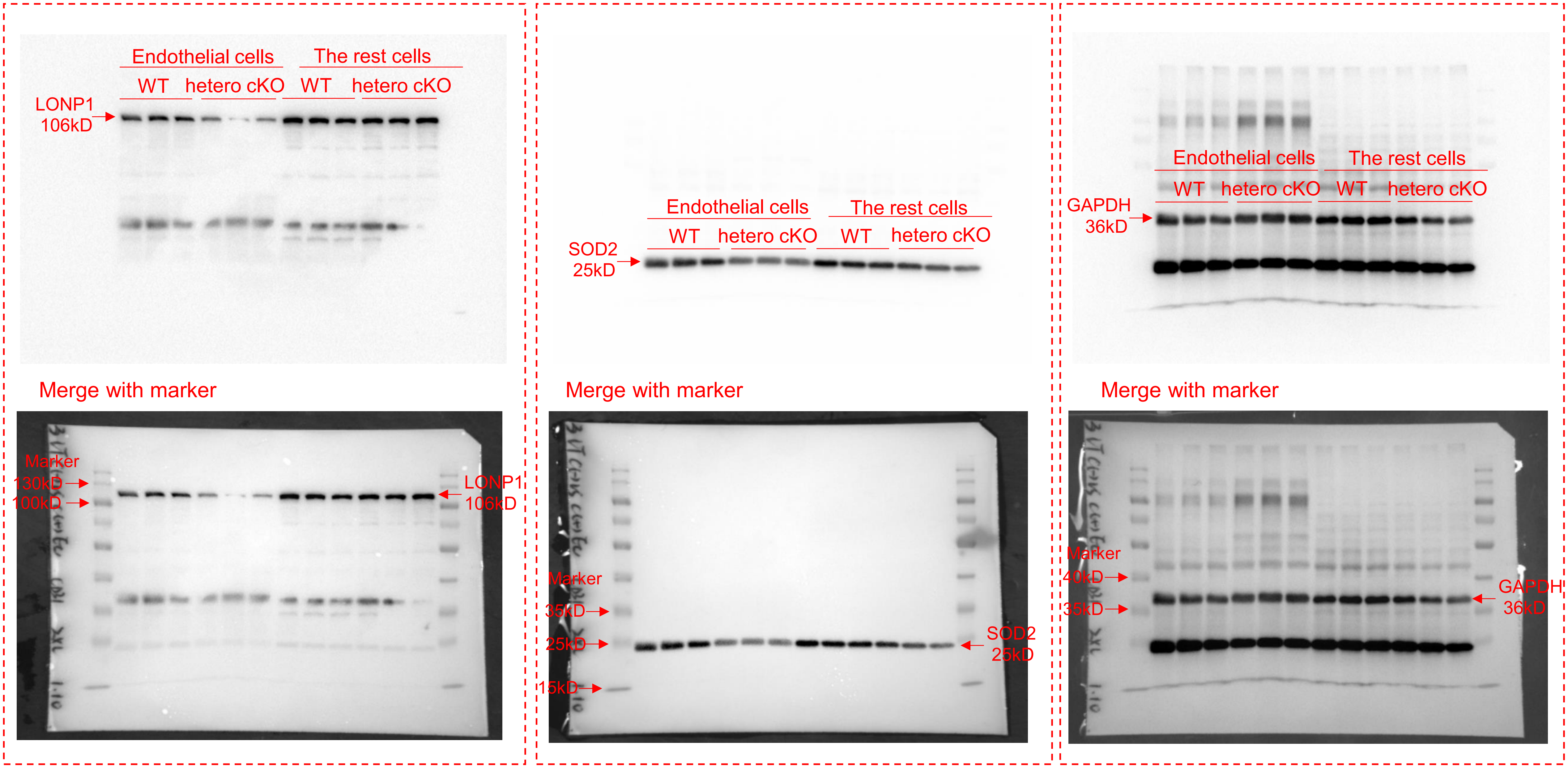

Fig2G Primary HAECs transfected with LONP1 overexpression plasmid

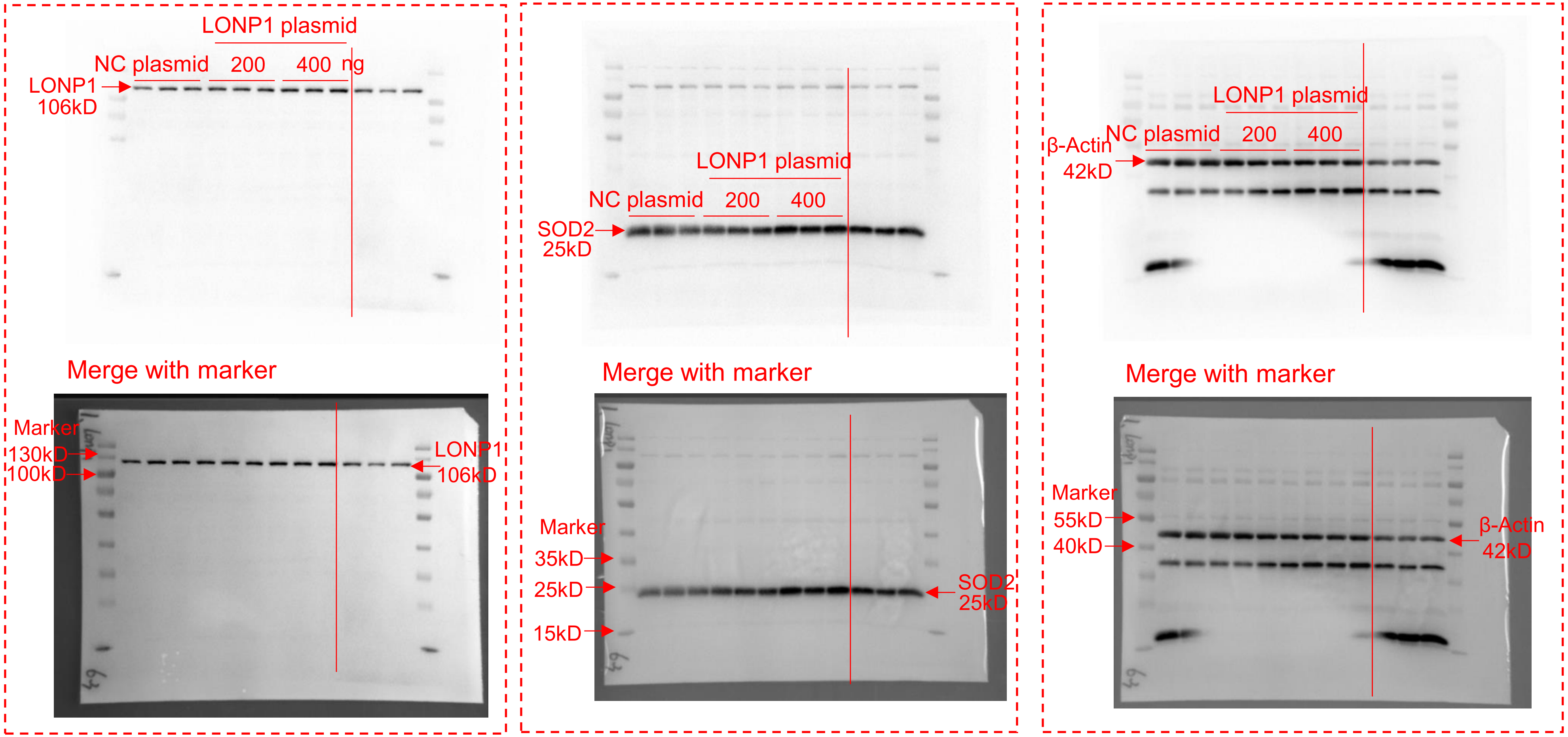

Fig2I MAECs transfected with LONP1 overexpression plasmid-Represented images

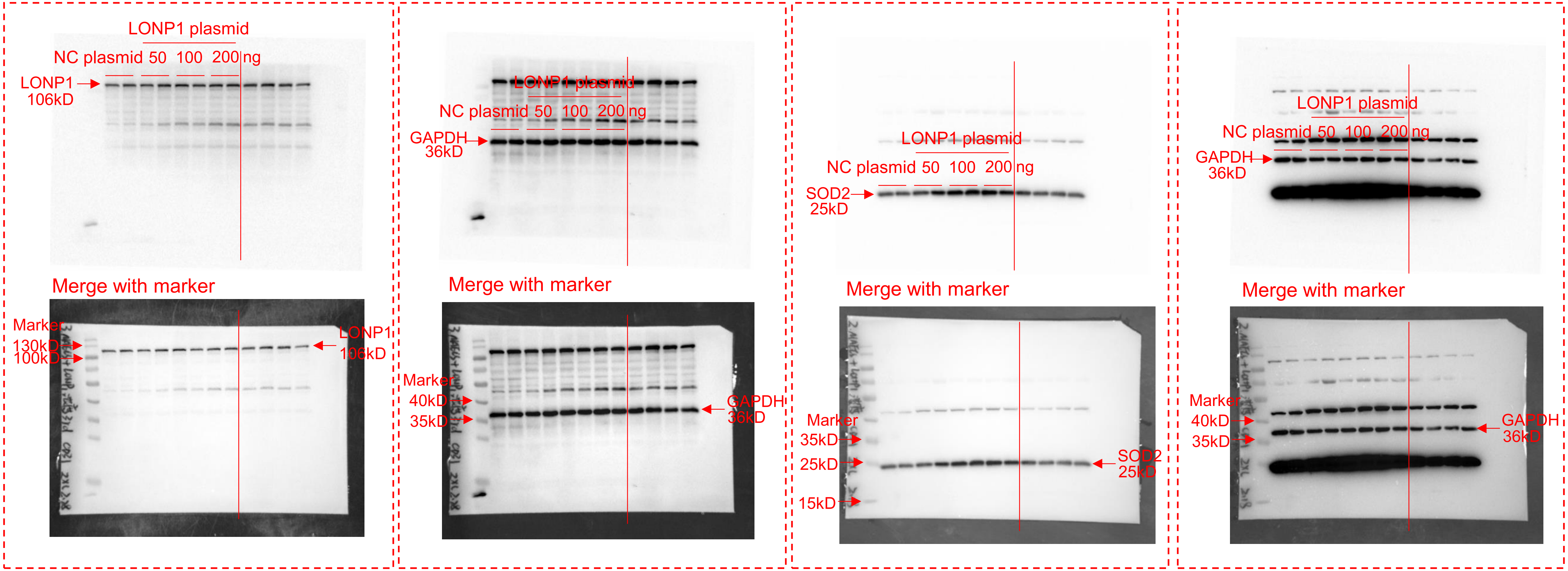

Fig2I MAECs transfected with LONP1 overexpression plasmid-Repeated images

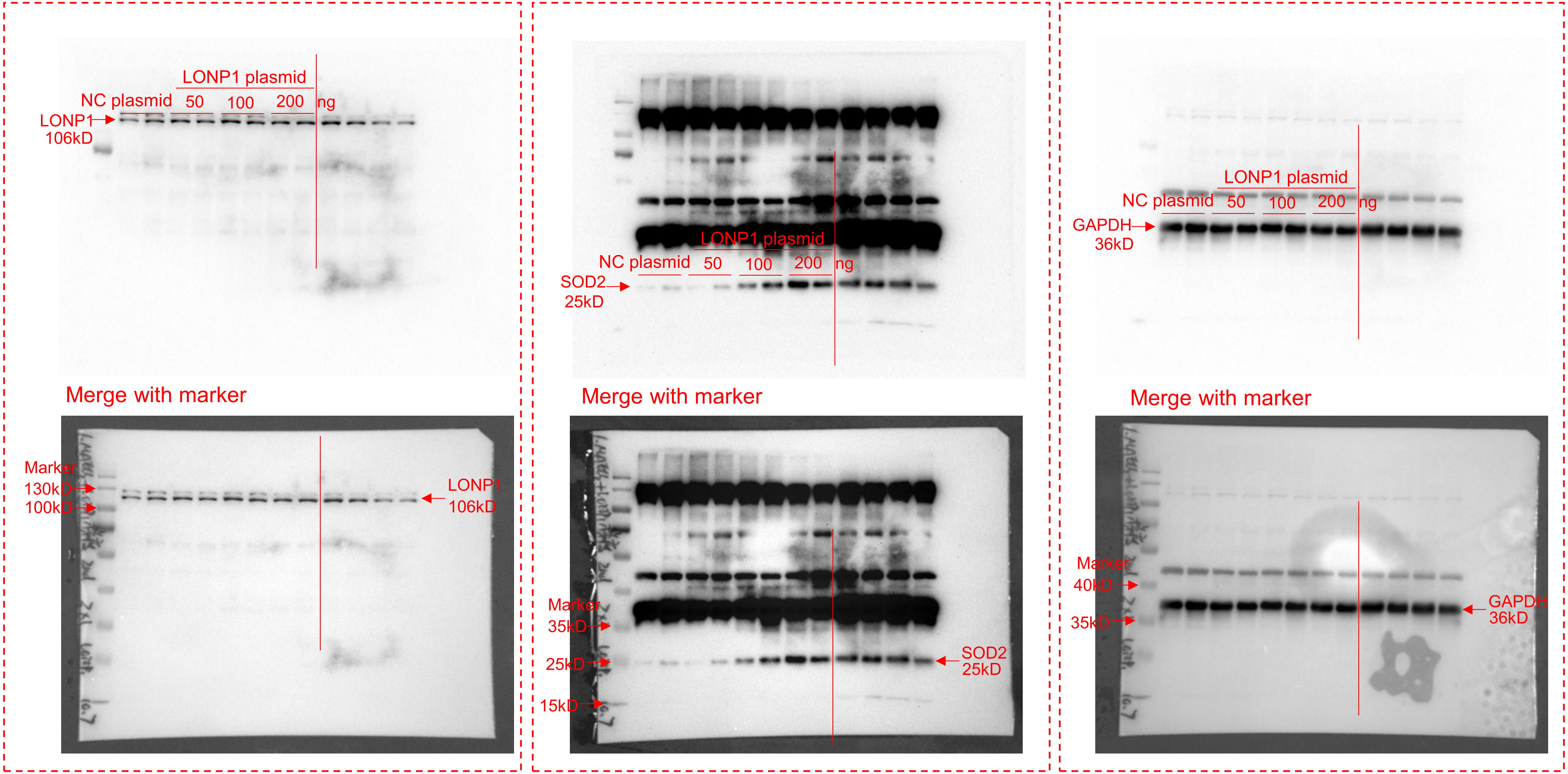

Fig2K Treatment with CHX after overexpression of LONP1 in MAECs-Represented images

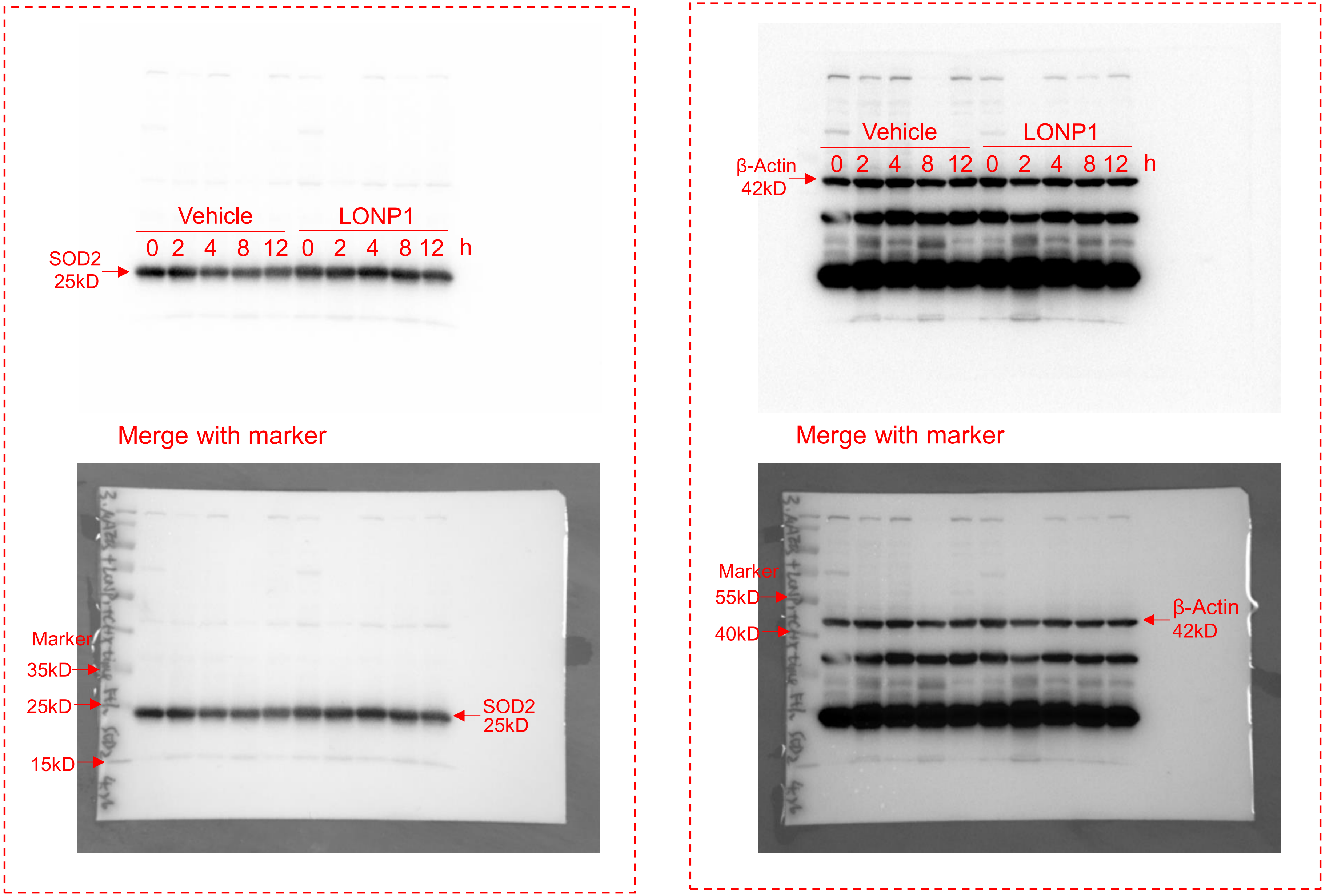

Fig2K

Treatment with CHX after overexpression of LONP1 in MAECs-Repeated images 1

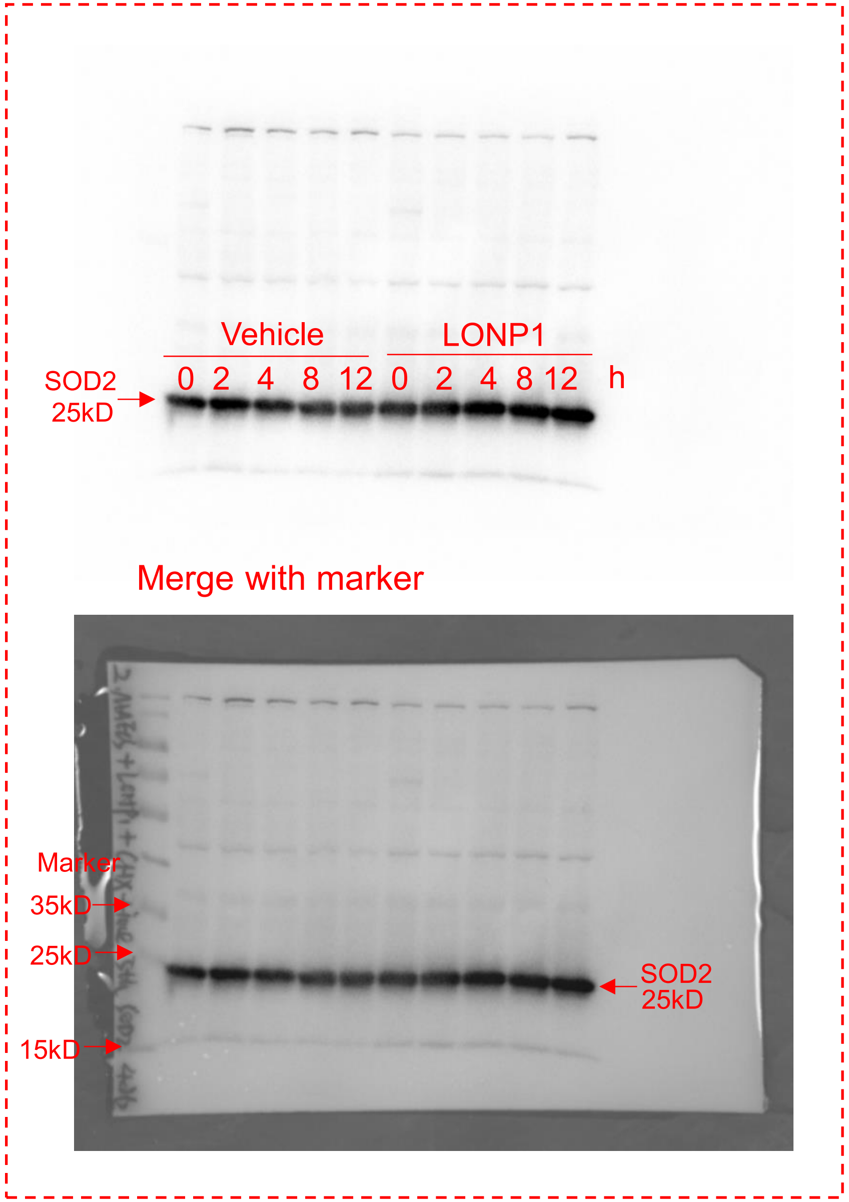

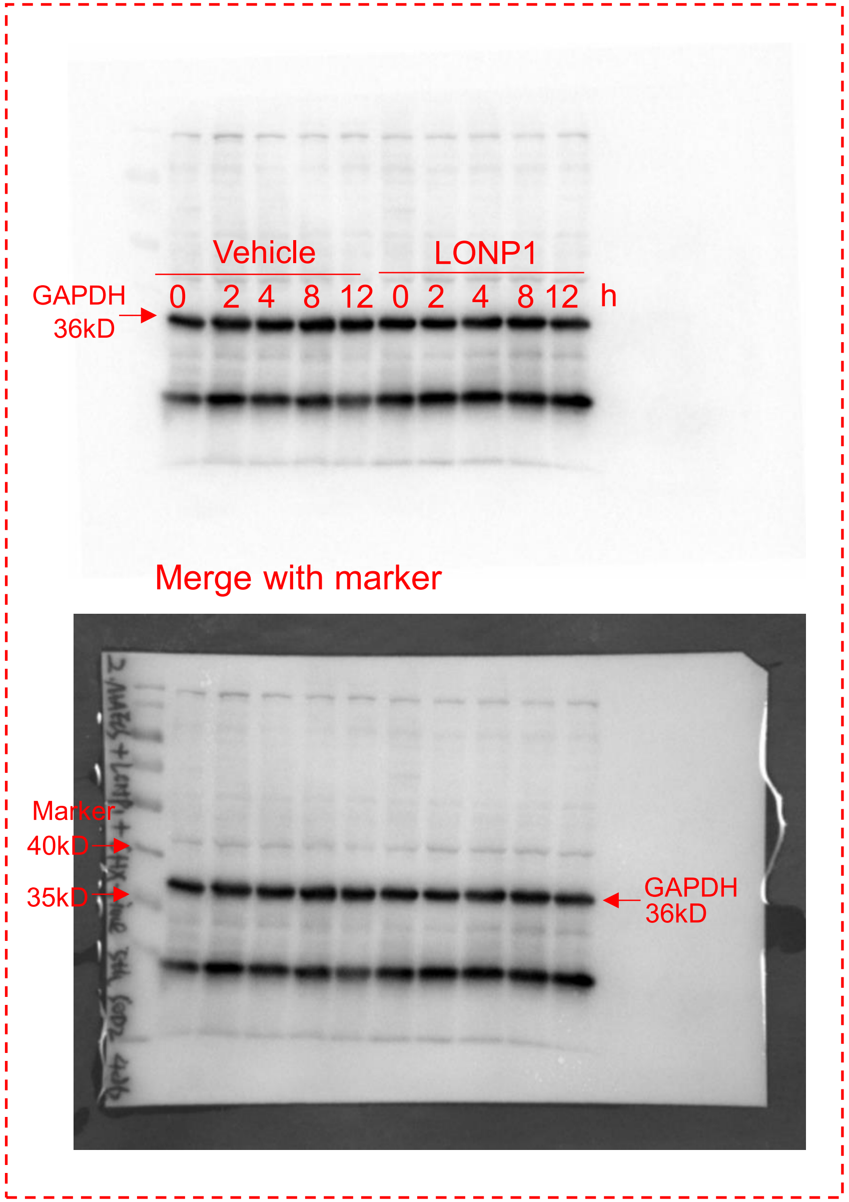

Fig2K

Treatment with CHX after overexpression of LONP1 in MAECs-Repeated images 2

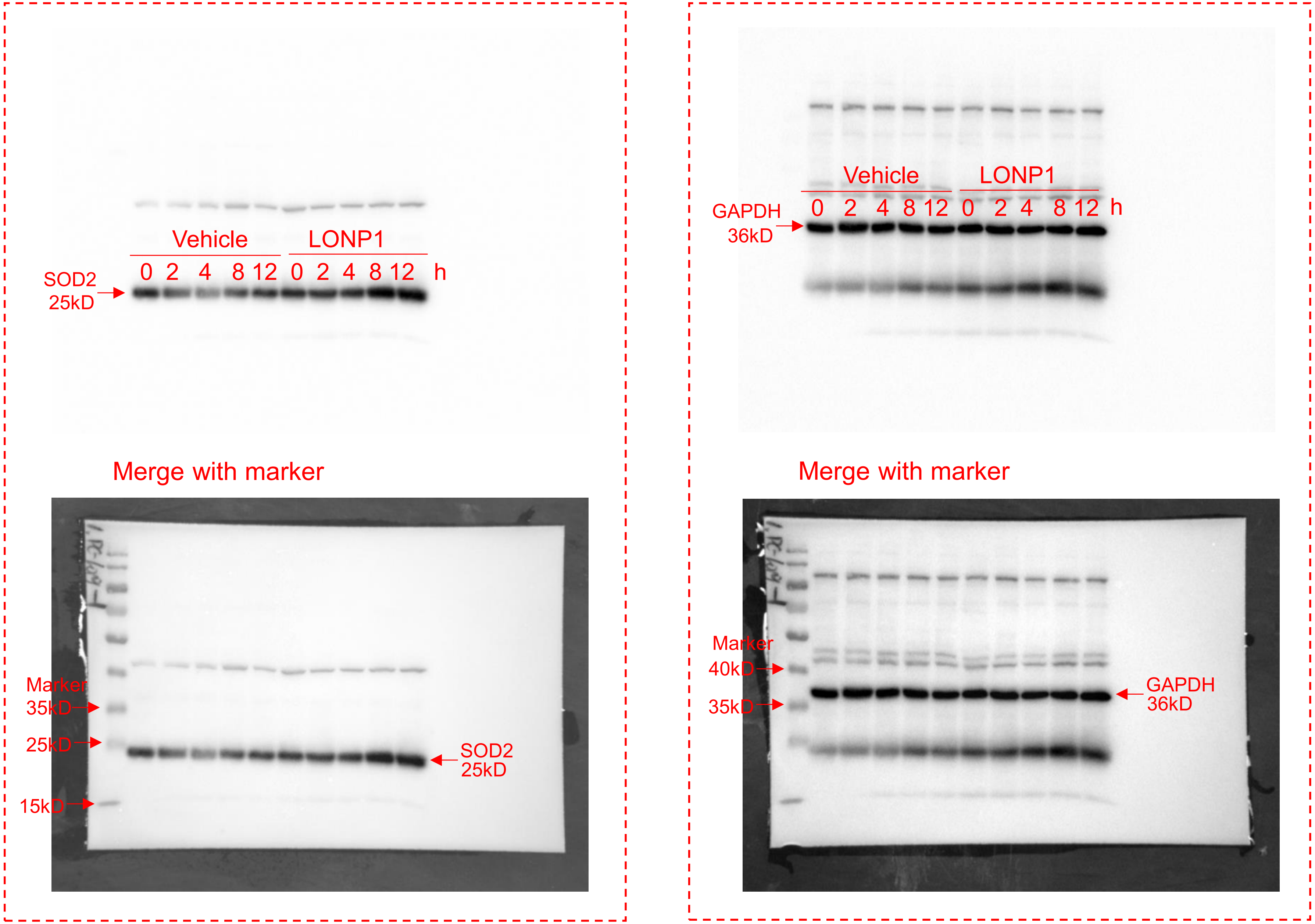

Fig2M 293T cell transfected with FLAG-LONP1, HA-SOD2, and MYC-Ub plasmid-IP

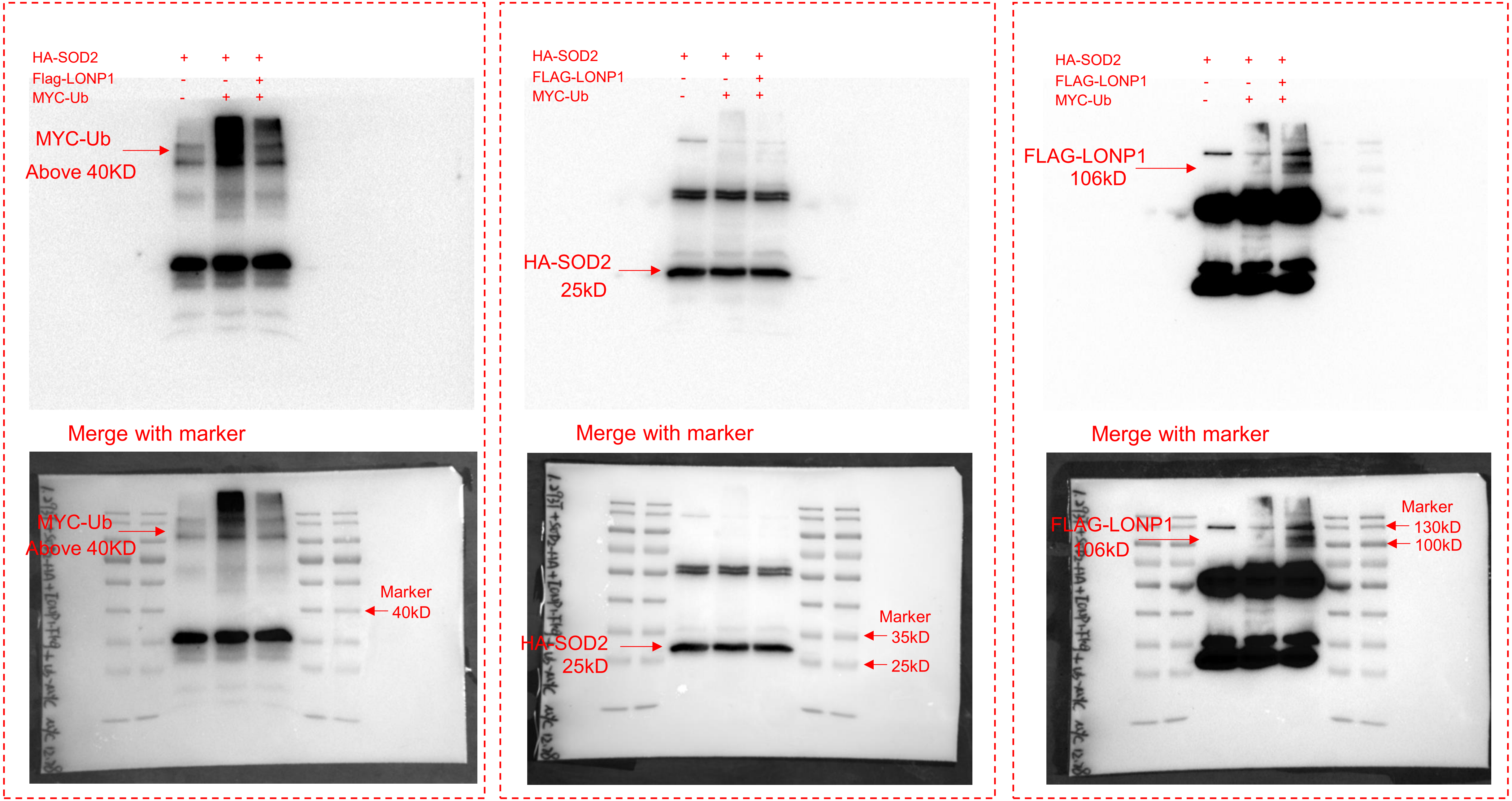

Fig2M 293T cell transfected with Flag-LONP1, HA-SOD2, and MYC-Ub plasmid-INPUT

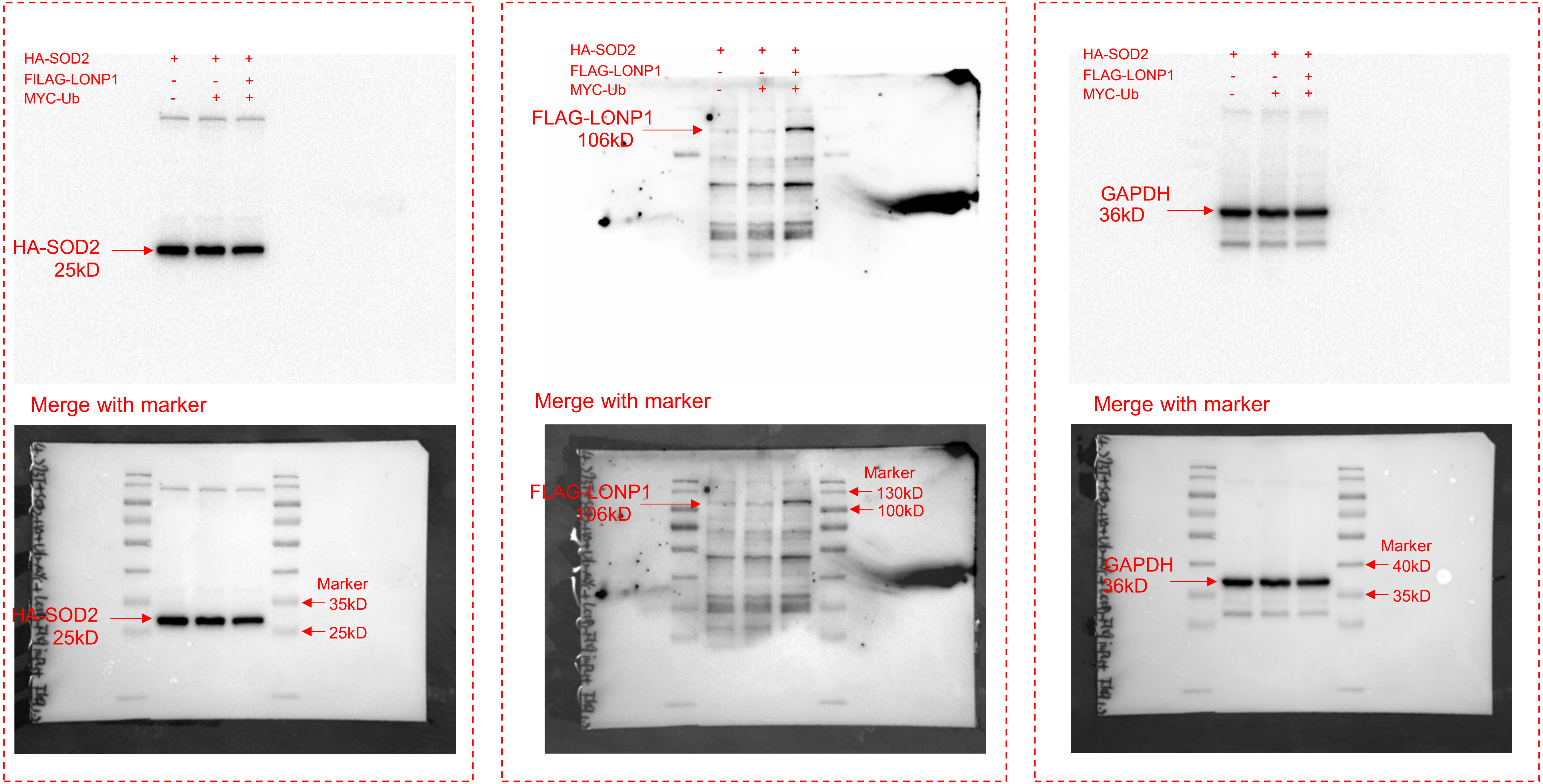

Fig2N 293T cell transfected with shLONP1, HA-SOD2, and MYC-Ub plasmid-IP

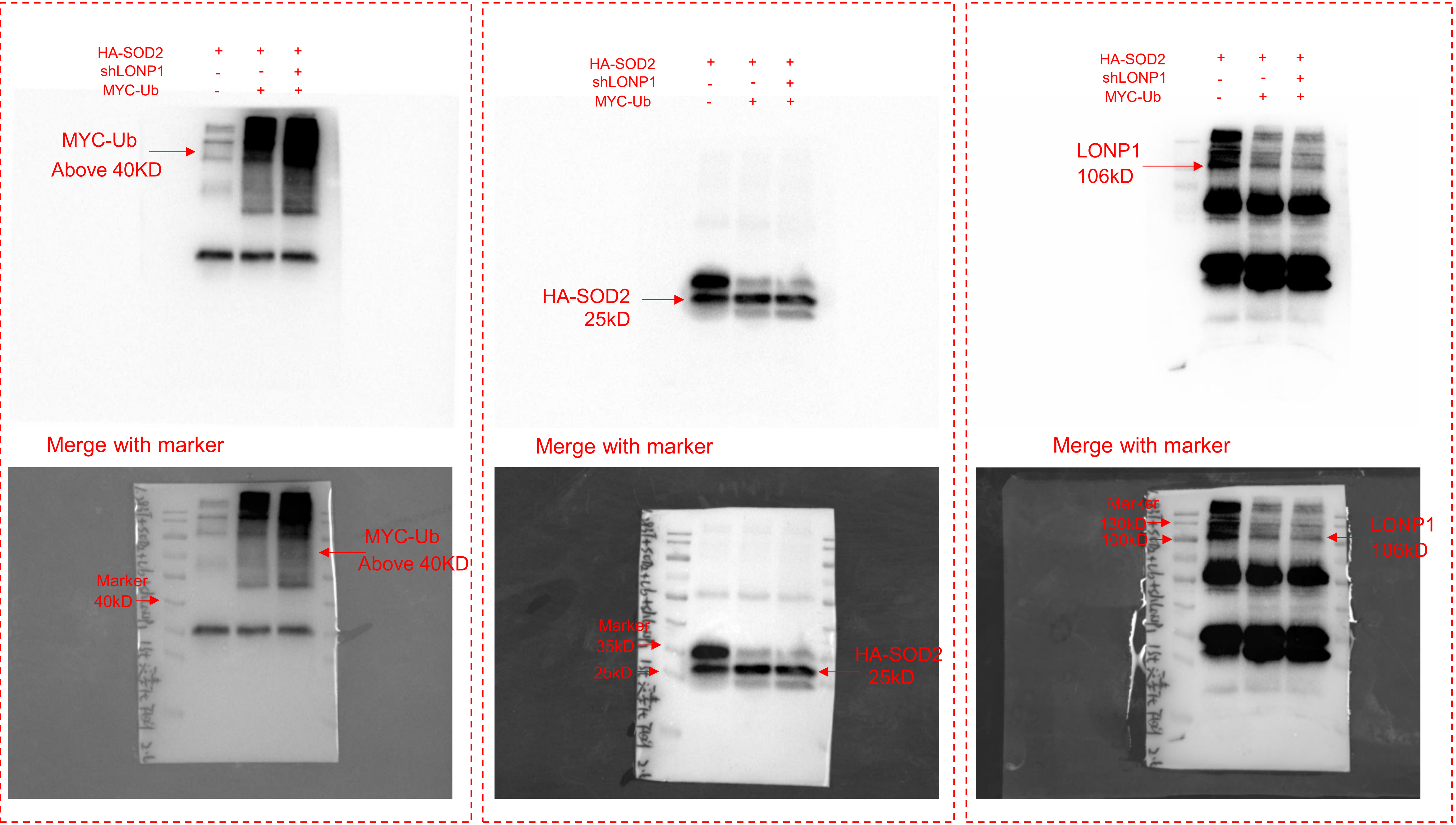

Fig2N 293T cell transfected with shLONP1, HA-SOD2, and MYC-Ub plasmid-INPUT

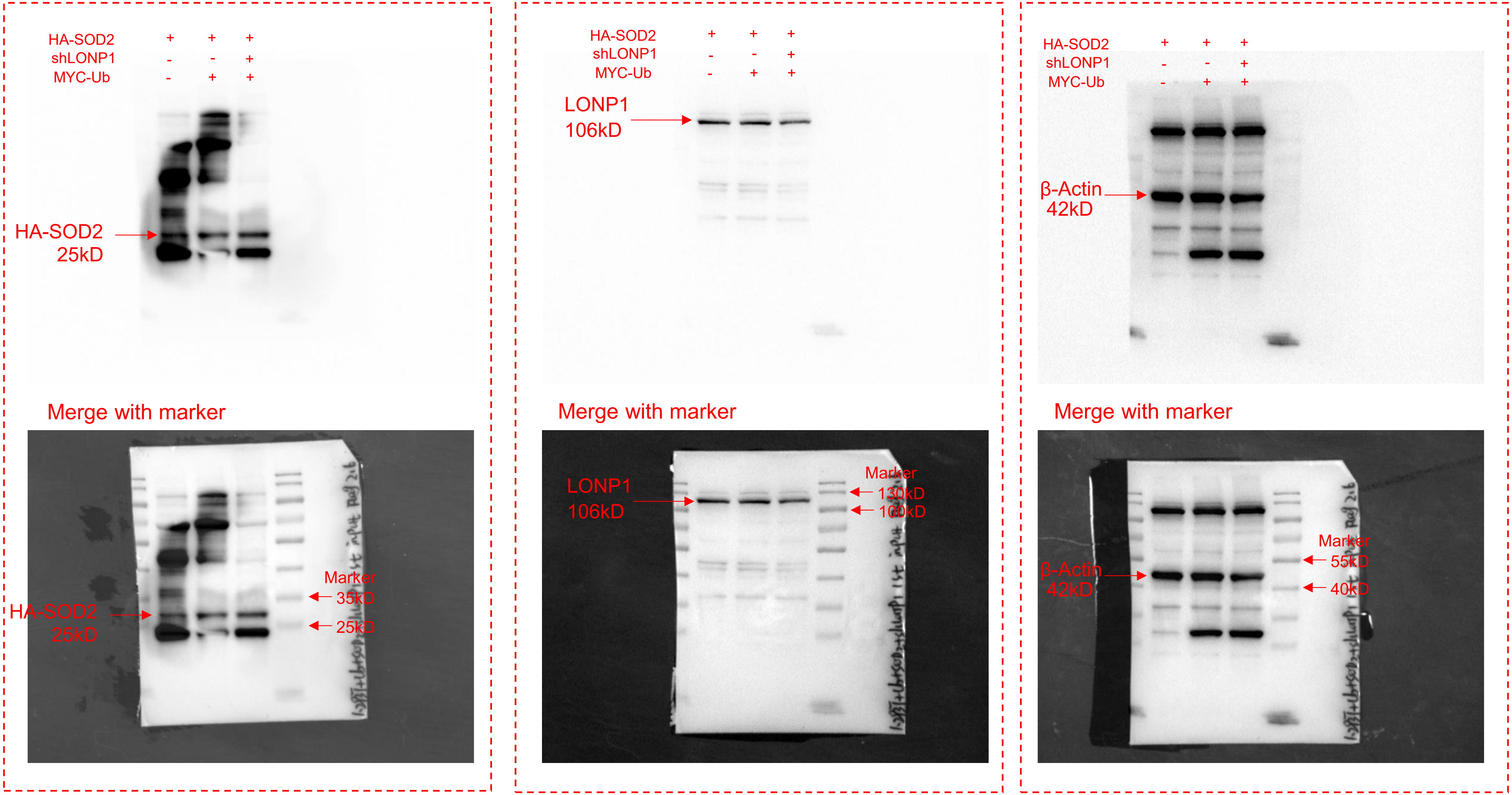

Fig2P MAECs transfected with SOD2 overexpression plasmid

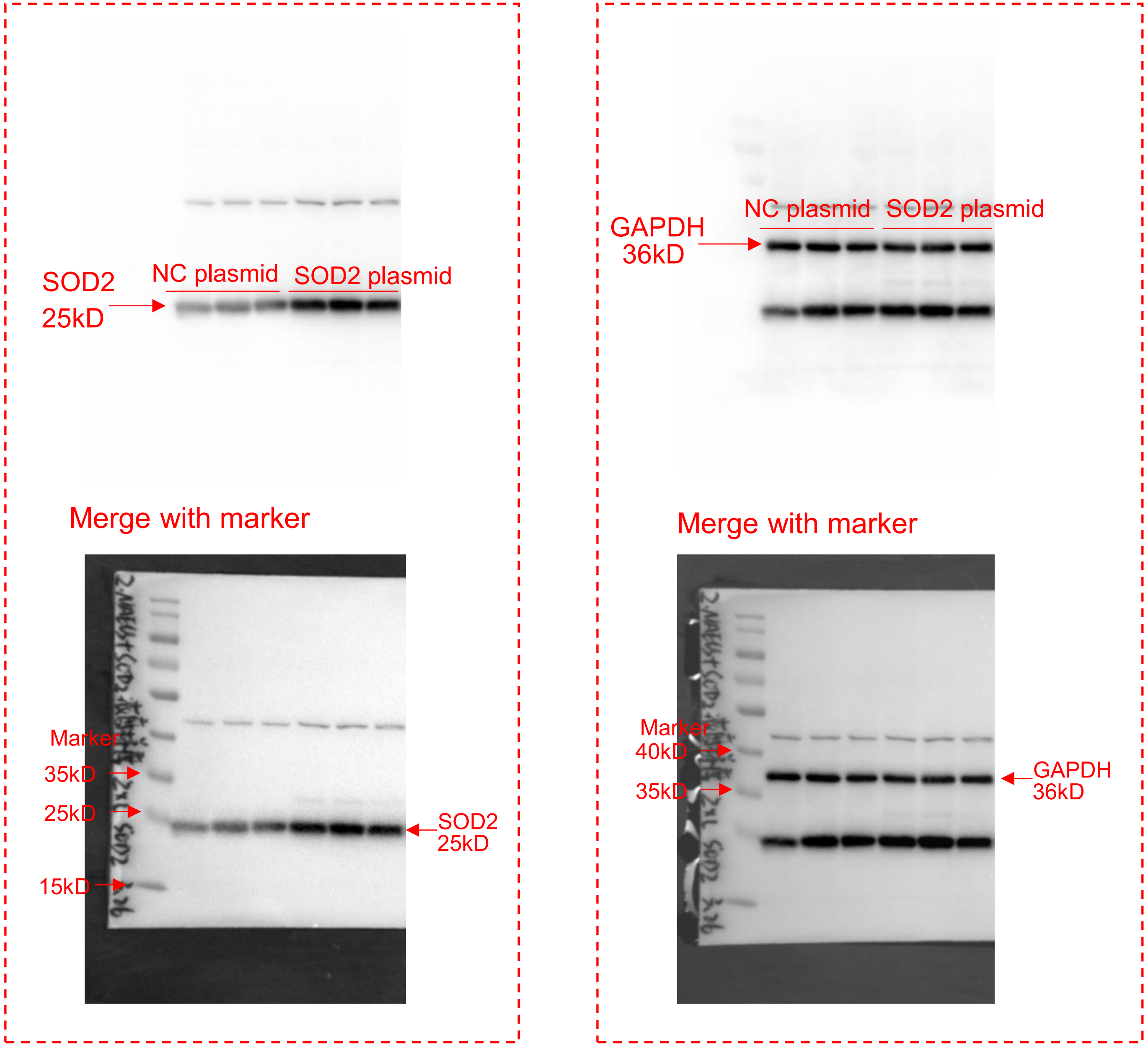

Fig2R MAECs transfected with LONP1 overexpression plasmid-COIP

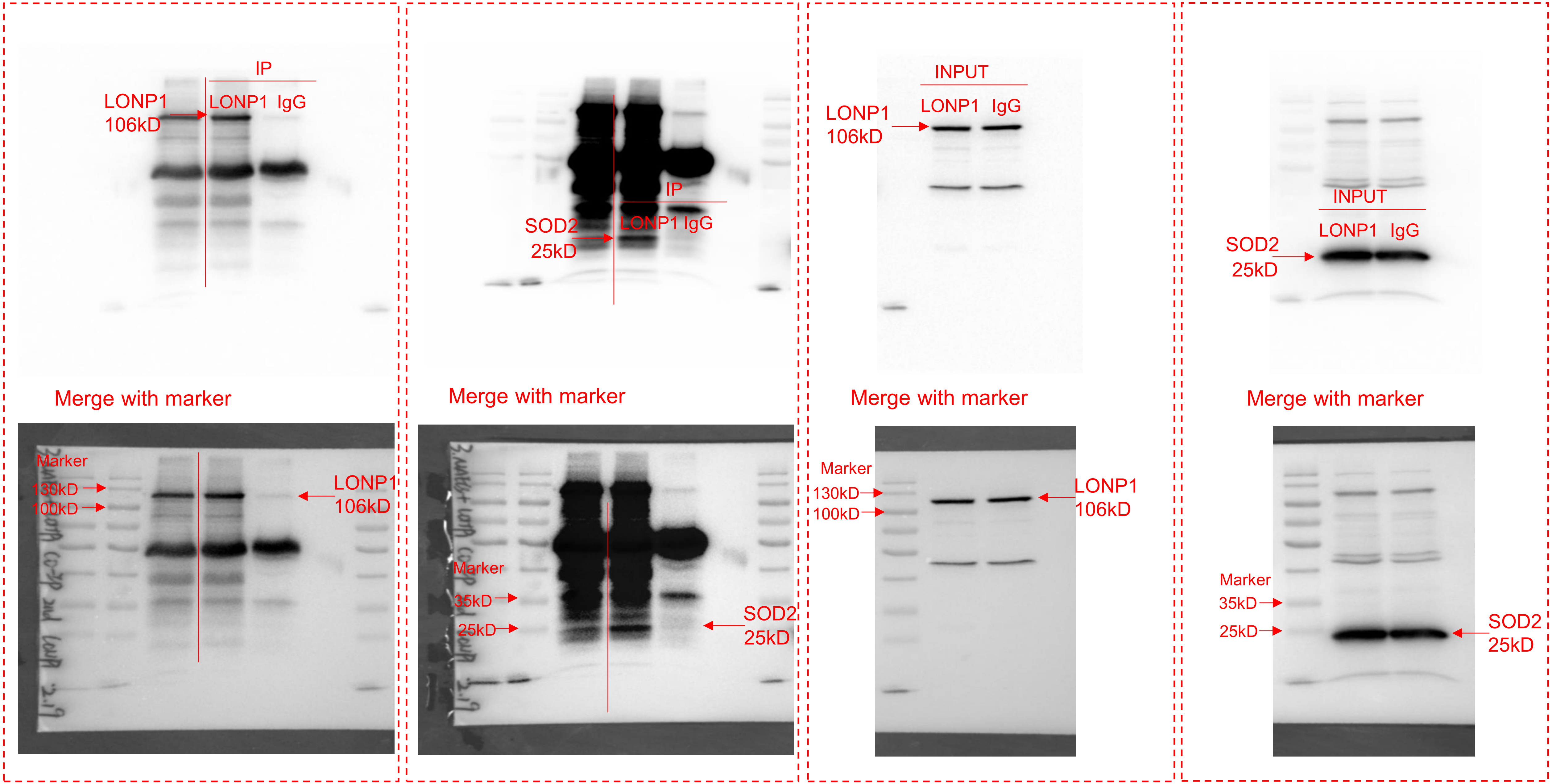

Fig2S MAECs transfected with SOD2 overexpression plasmid-COIP

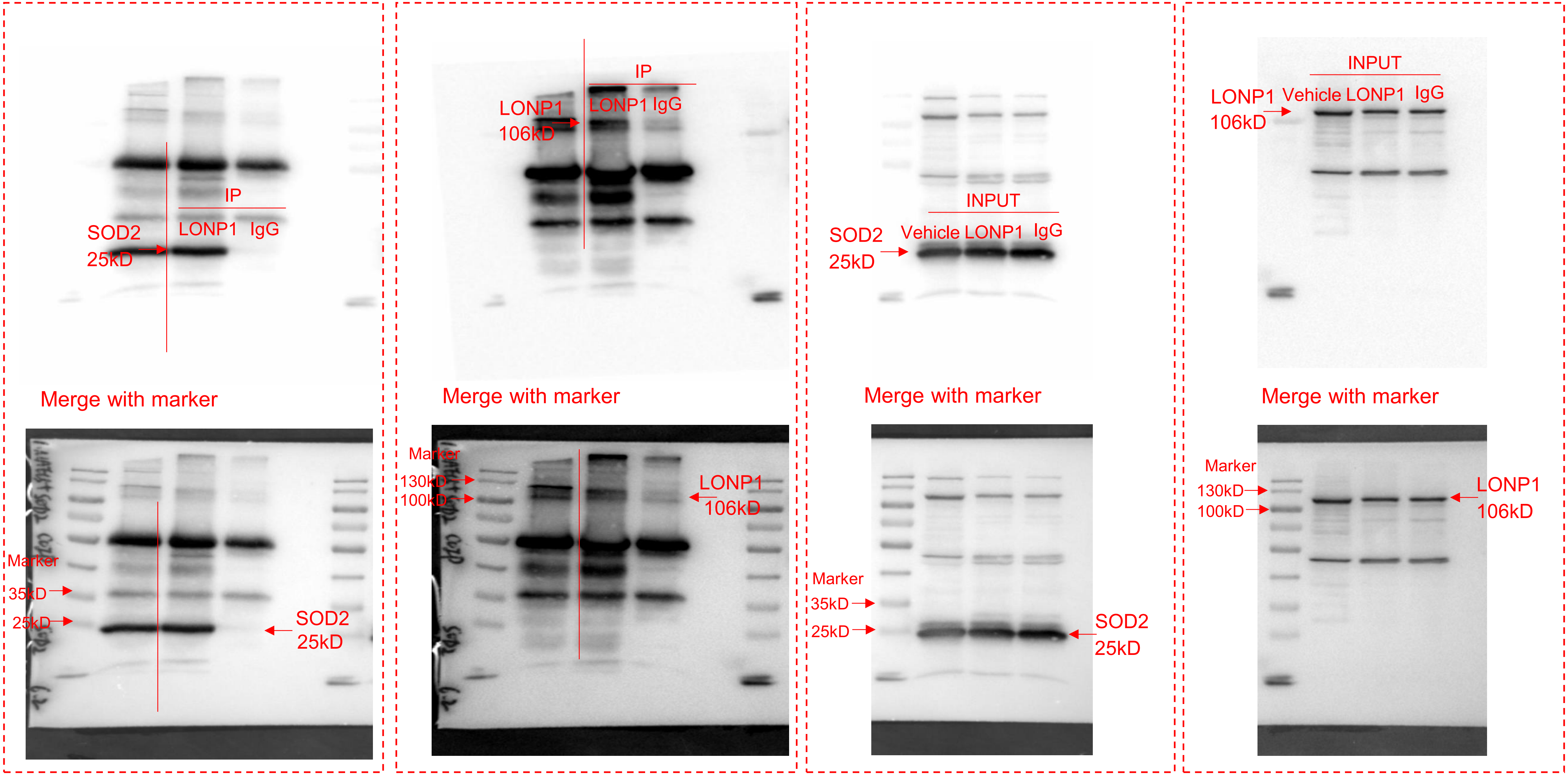

Fig2Y MAECs transfected with FLAG-LONP1-Frag3 plasmid-COIP

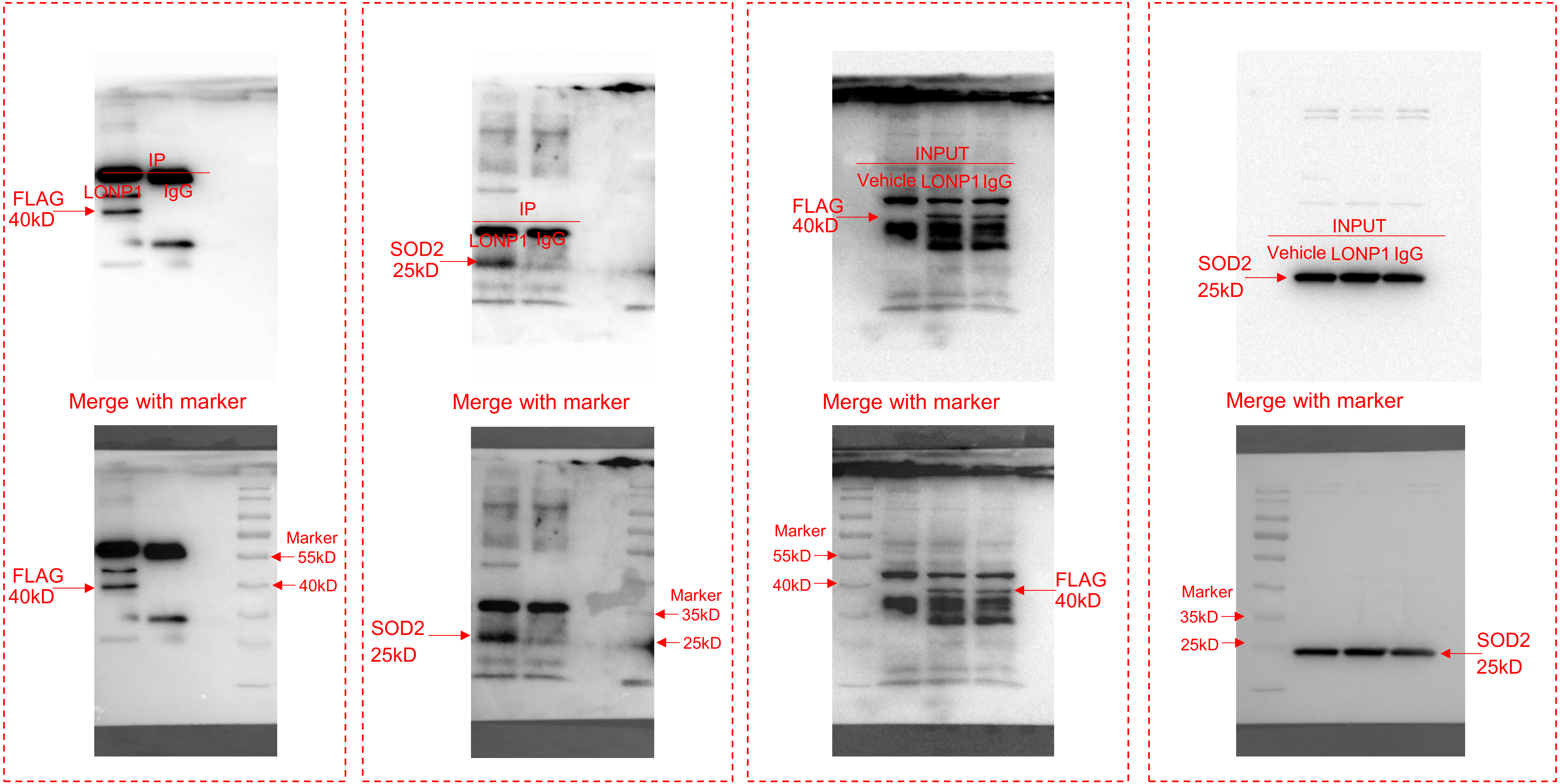

Fig2Z MAECs transfected with FLAG-LONP1-MUT plasmid-COIP

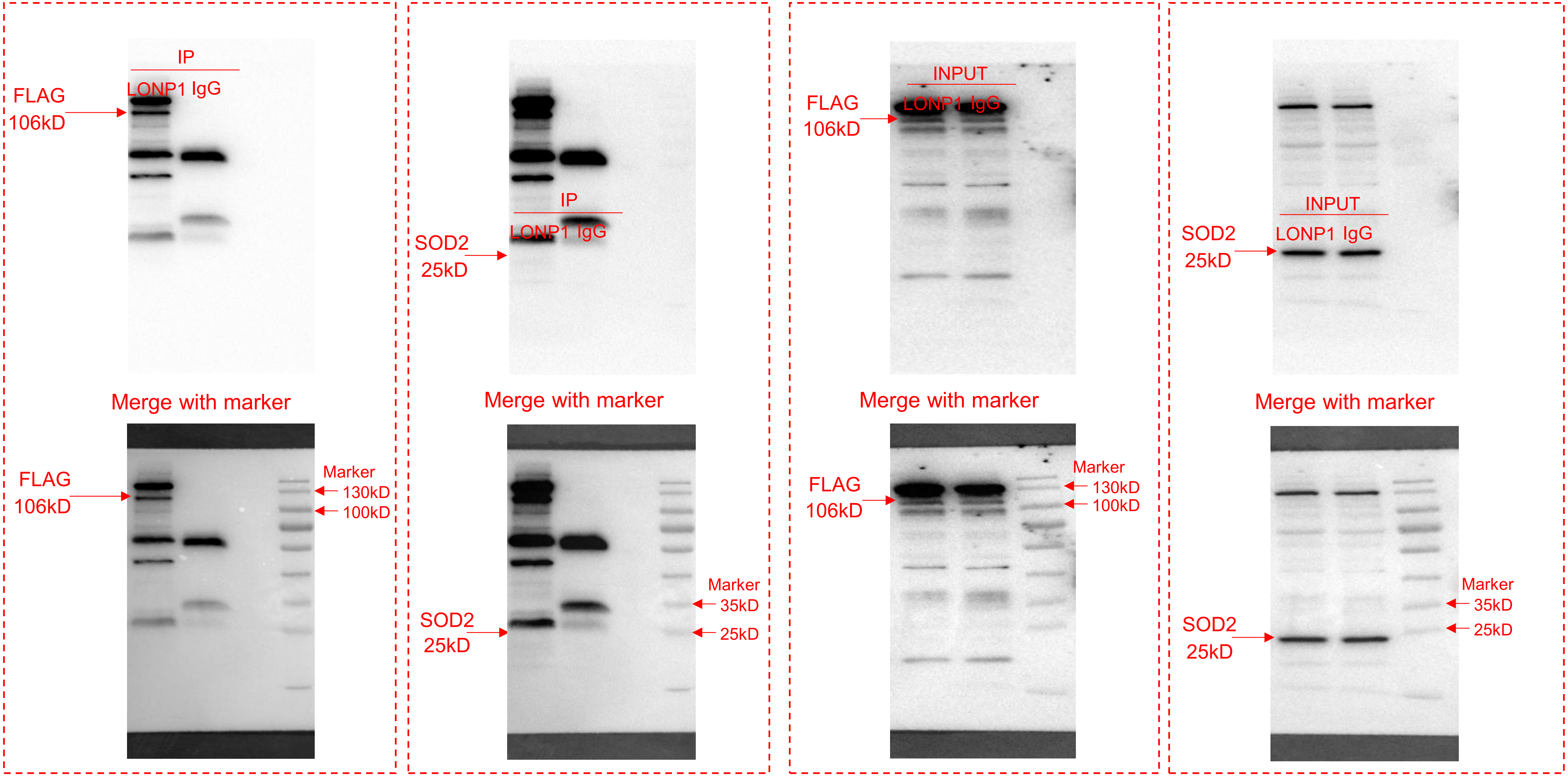

Fig4S Primary HAECs transfected with shLONP1 plasmid

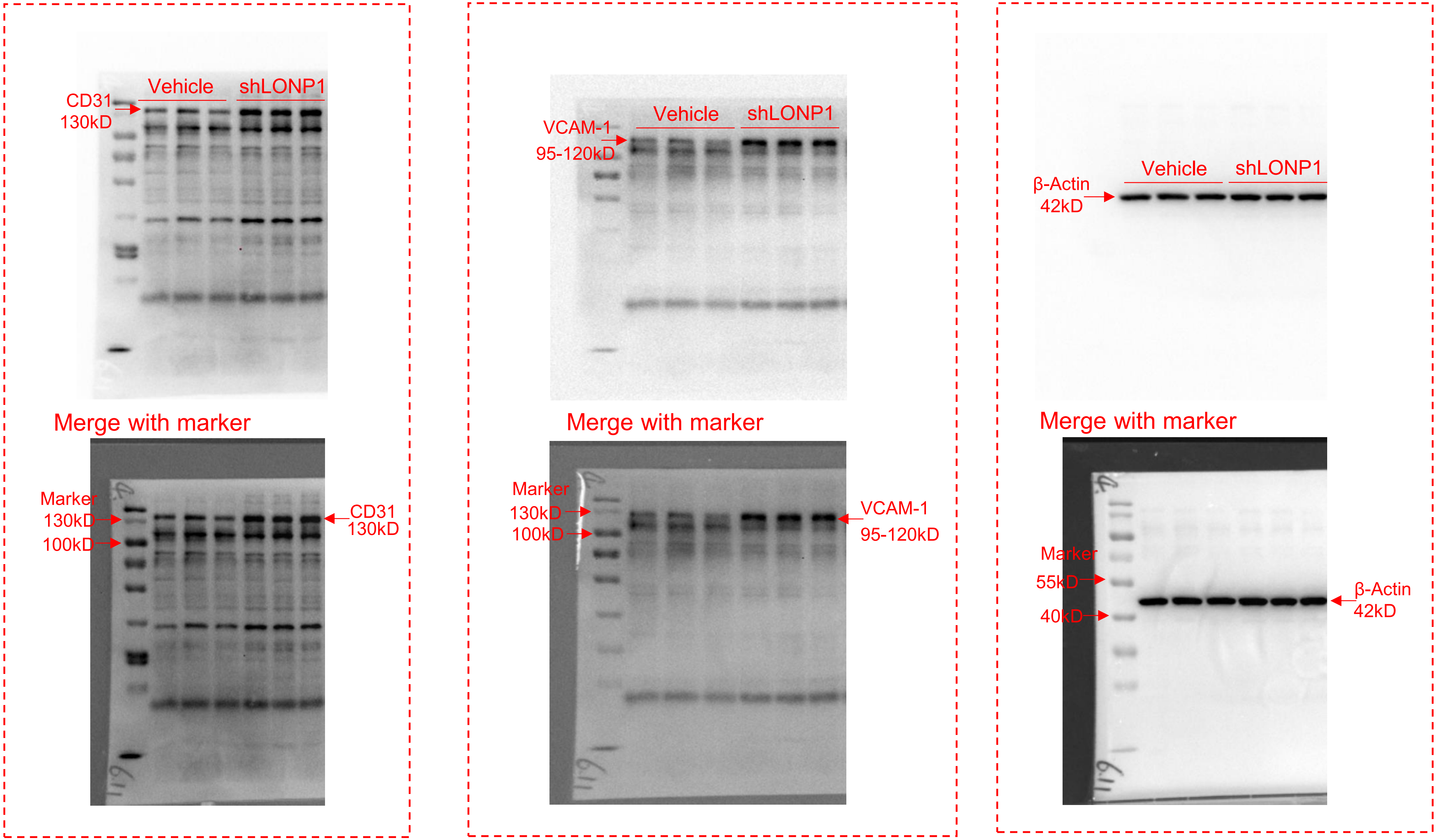

Fig4S Primary HAECs transfected with shLONP1 plasmid

Antibody of ABclonal (A5597)

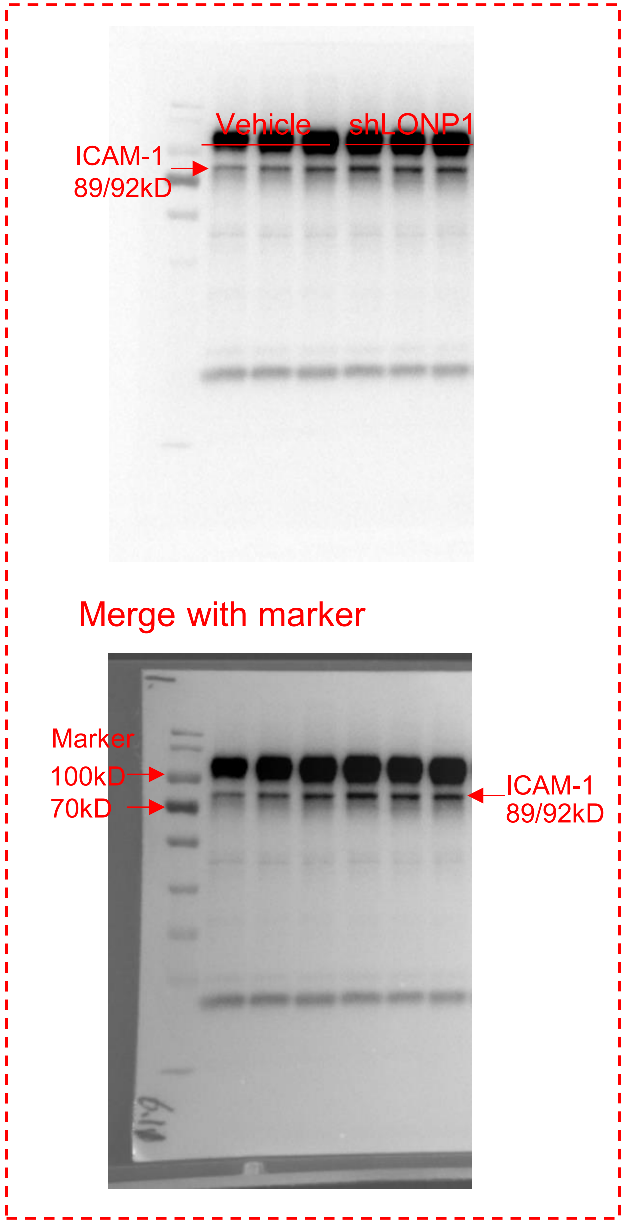

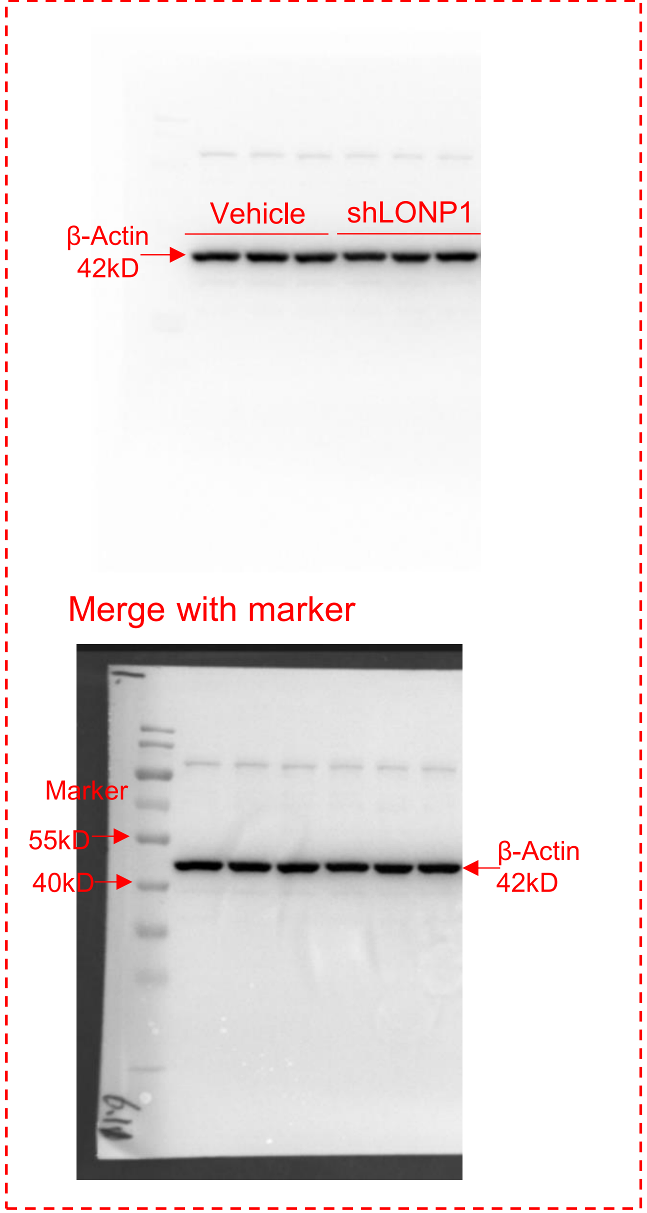

Fig4U MAECs transfected with shLONP1 plasmid

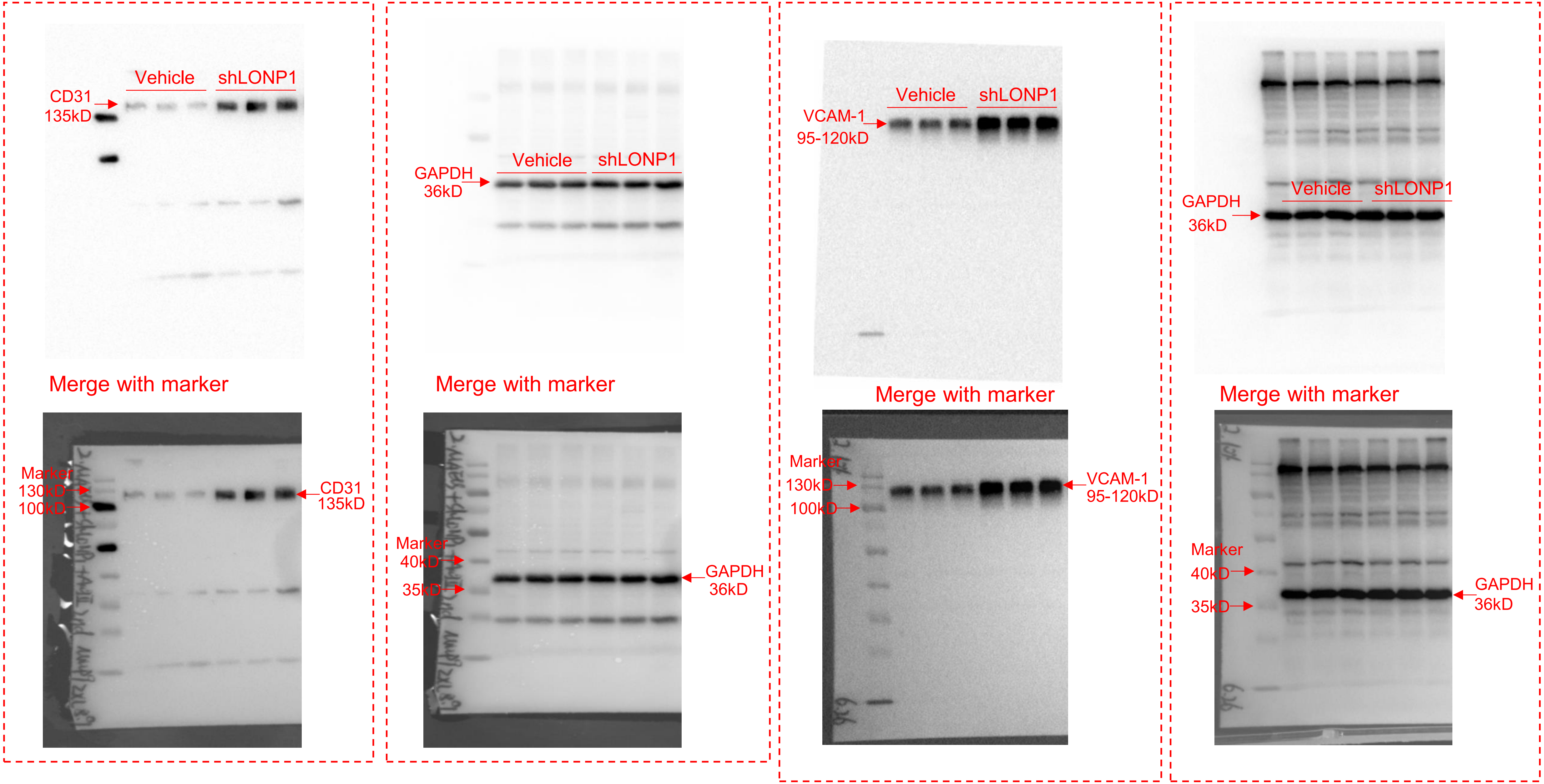

Fig4U MAECs transfected with shLONP1 plasmid

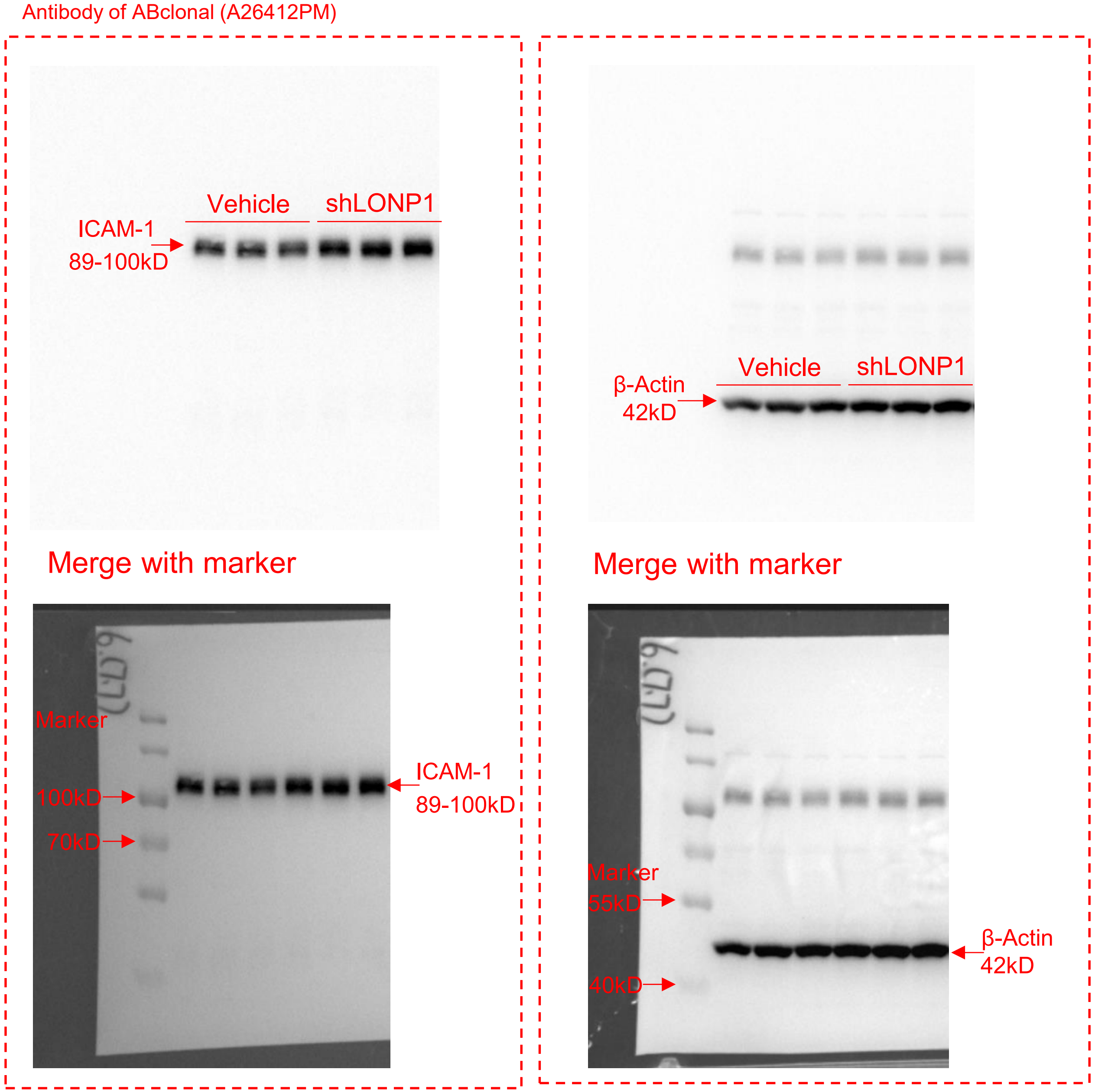

Fig4Y Immortalized HAECs transfected with LONP1 overexpression plasmid and treated with Ang II

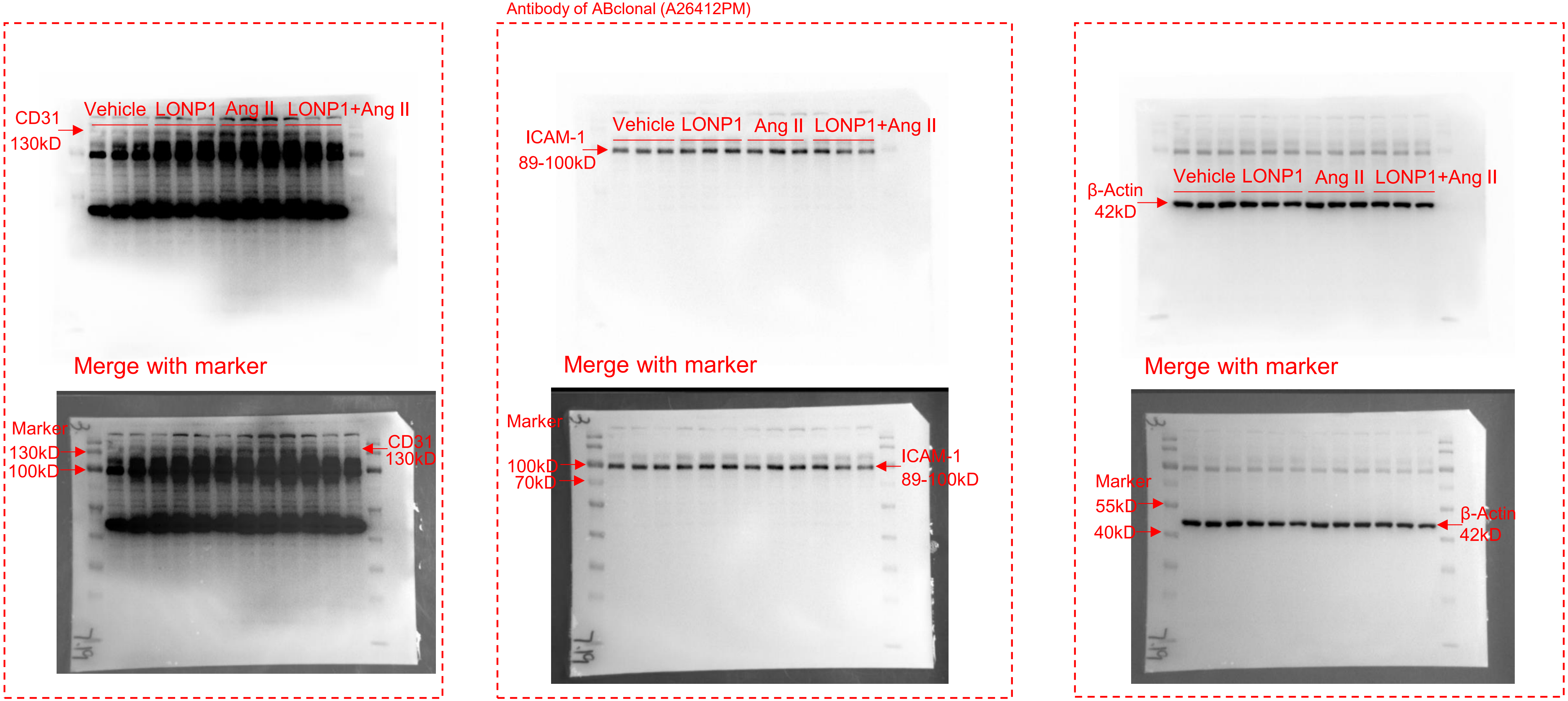

Fig4Y Immortalized HAECs transfected with LONP1 overexpression plasmid and treated with Ang II

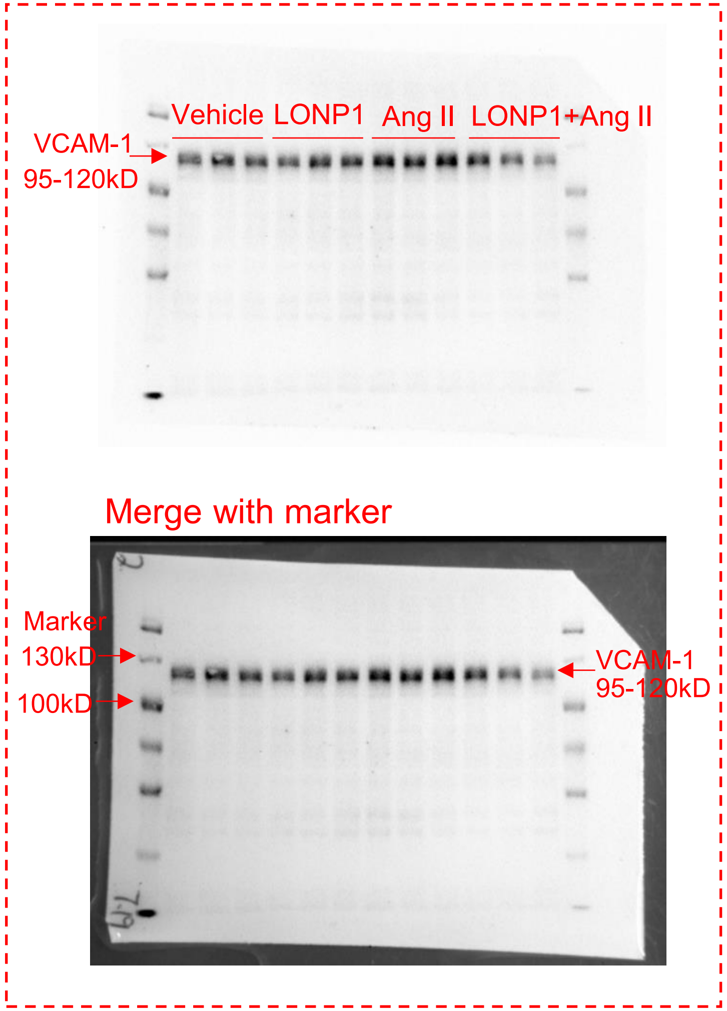

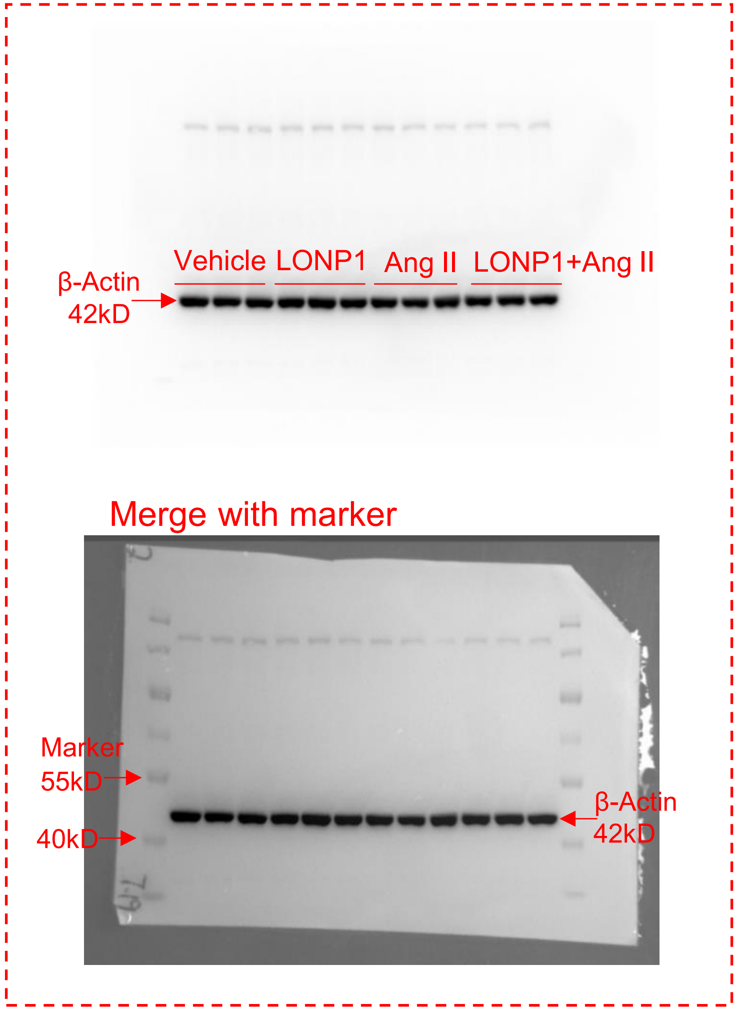

Fig4AA MAECs transfected with LONP1 overexpression plasmid and treated with Ang II

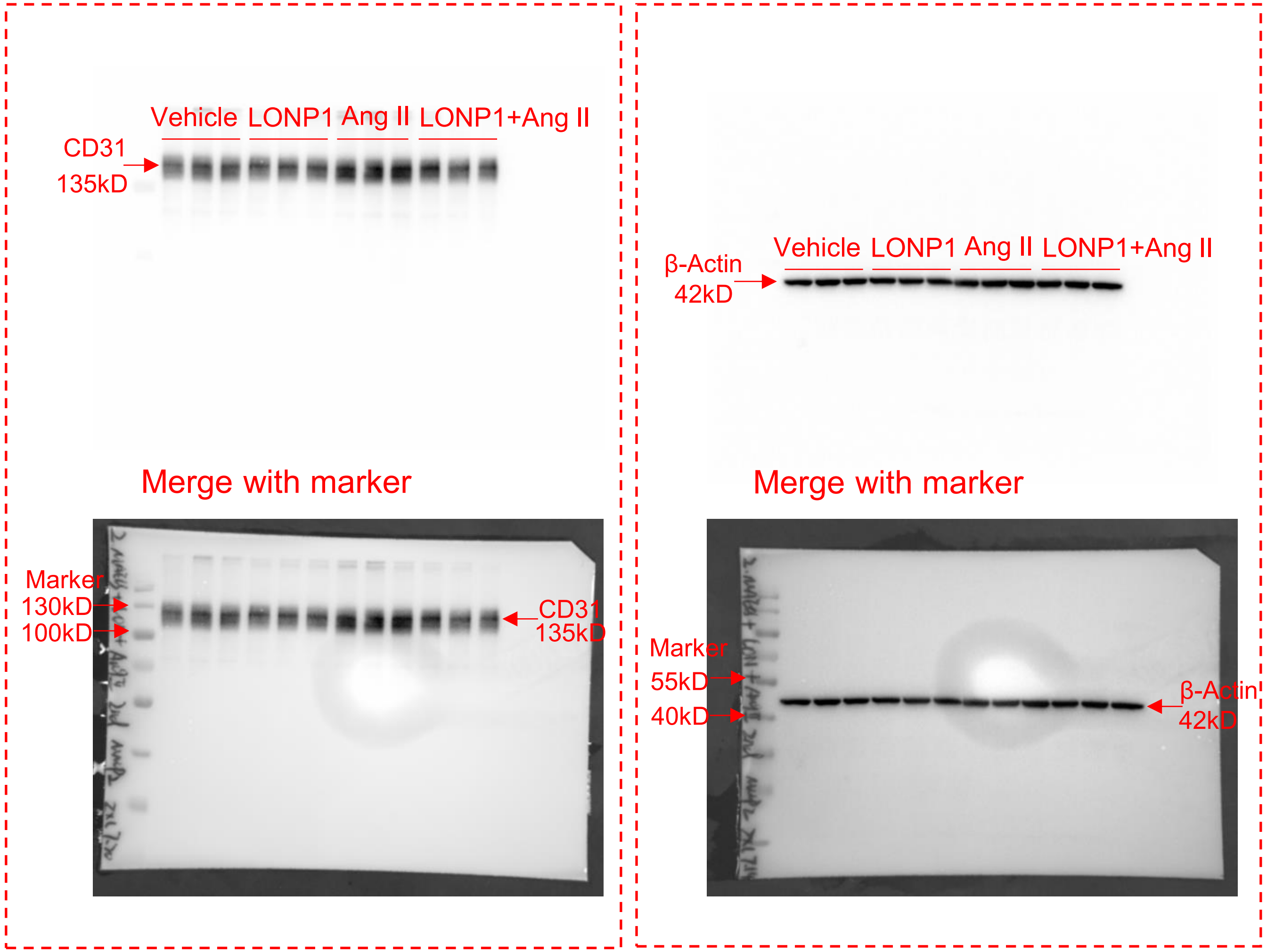

Fig4AA MAECs transfected with LONP1 overexpression plasmid and treated with Ang II

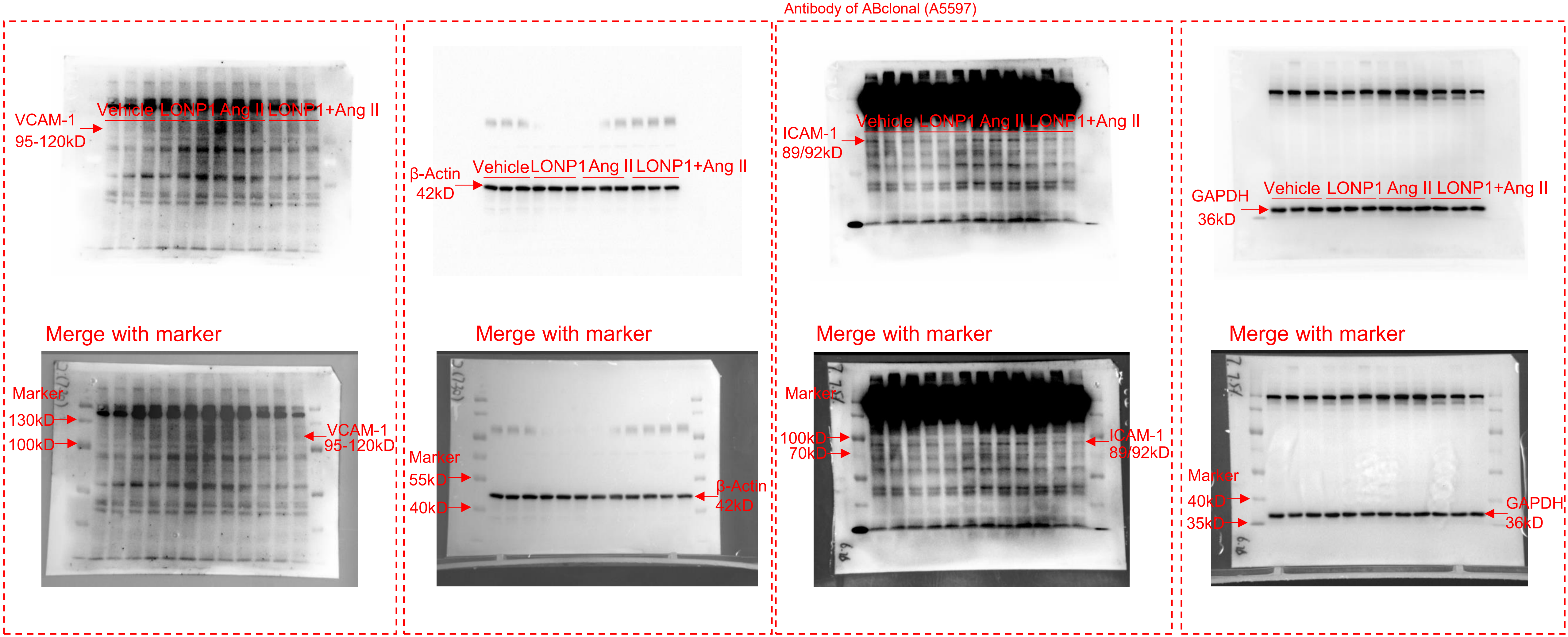

Fig5J LONP cKO mice treated with MnTBAP after constructing by 5/6Nx model

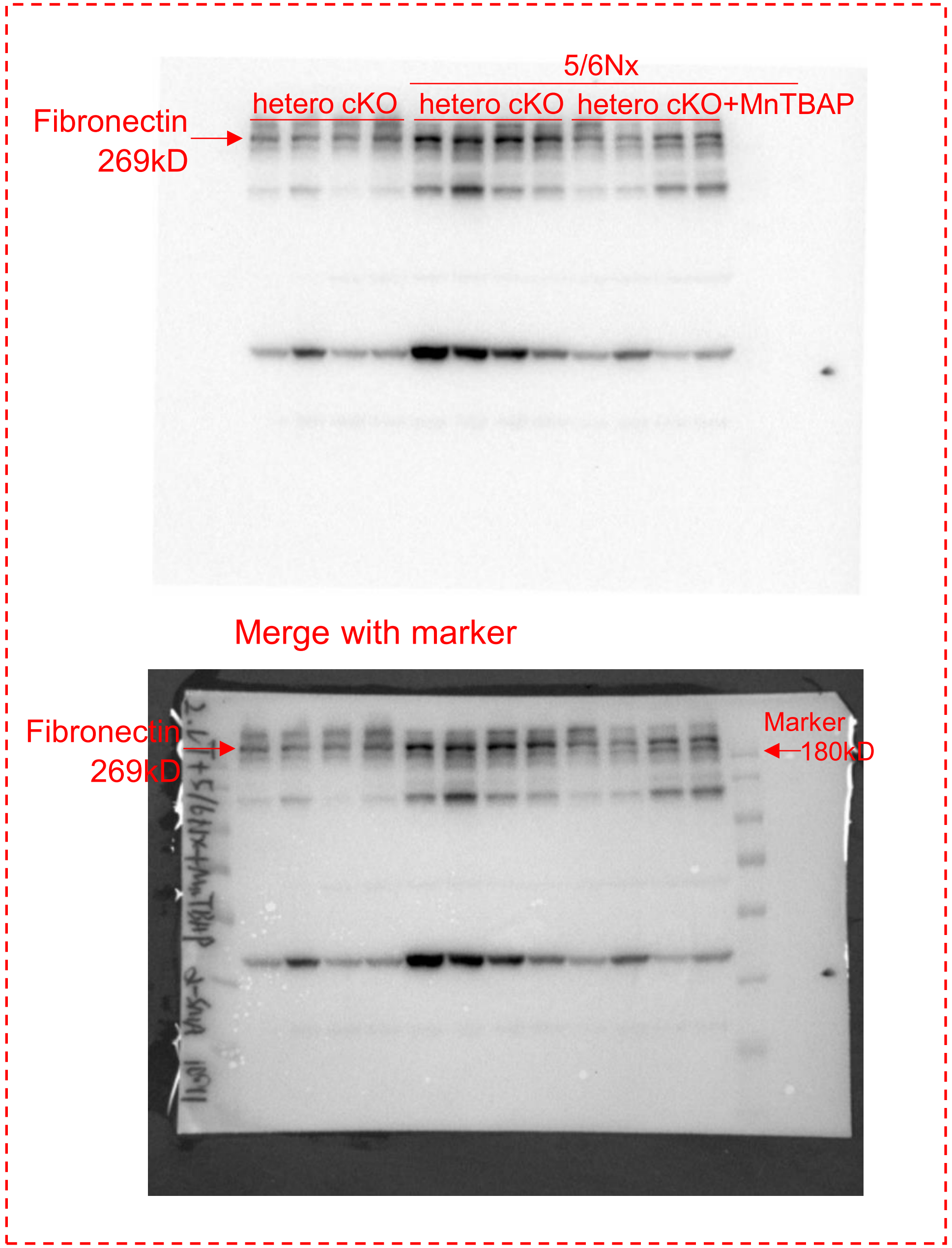

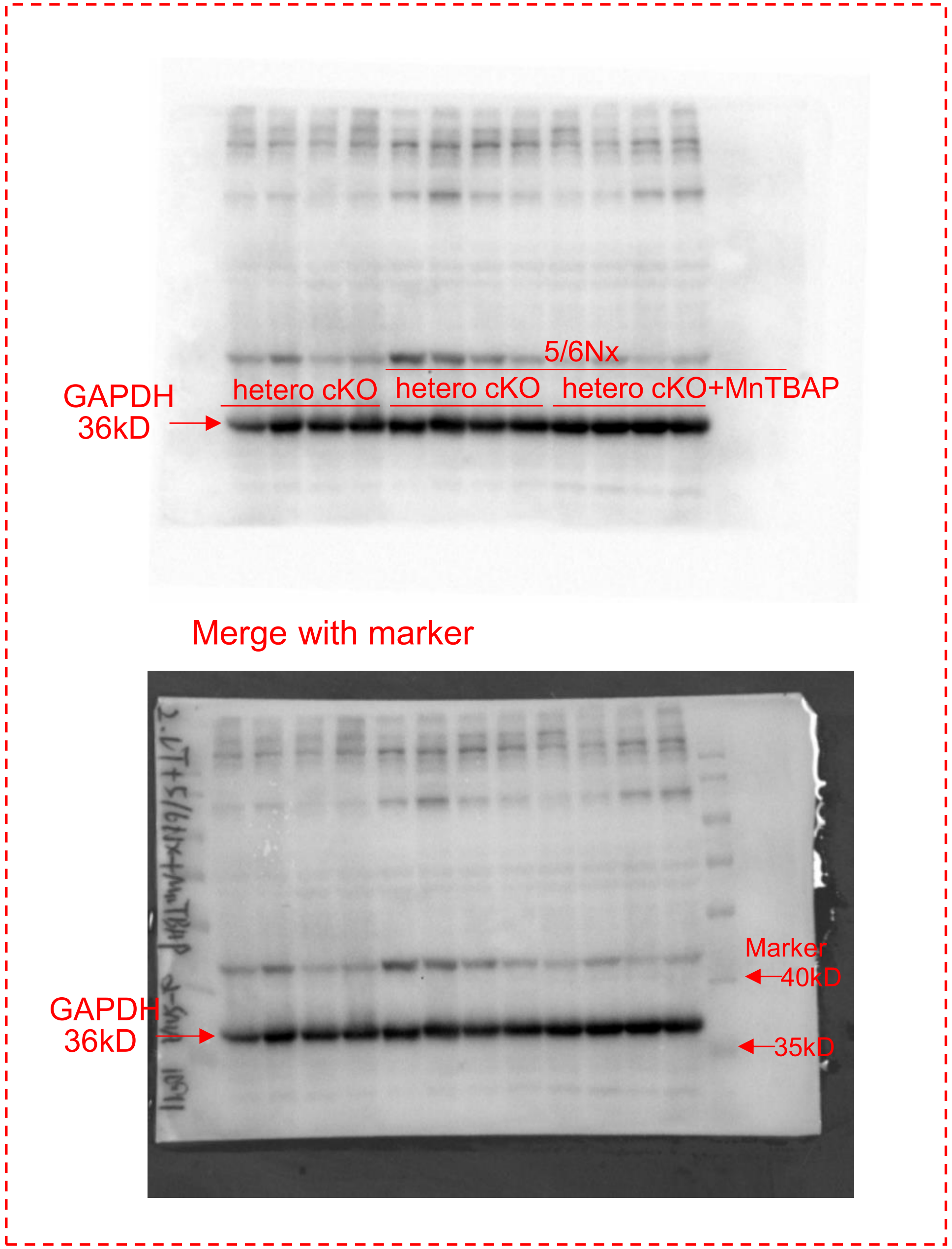

Fig5N Immortalized HAECs transfected with shLONP1 plasmid and treated with MnTBAP

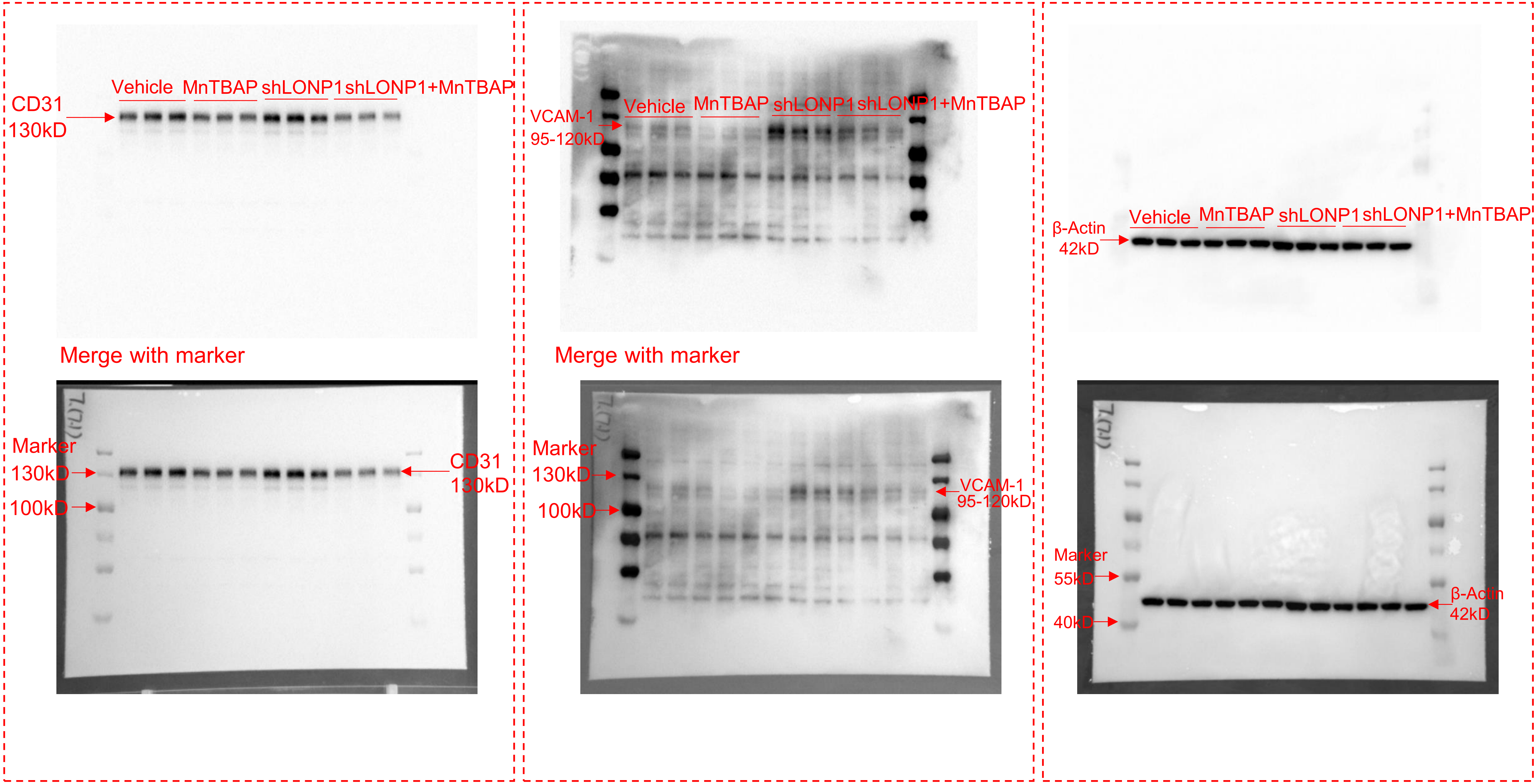

Fig5N Immortalized HAECs transfected with shLONP1 plasmid and treated with MnTBAP

Antibody of ABclonal (A5597)

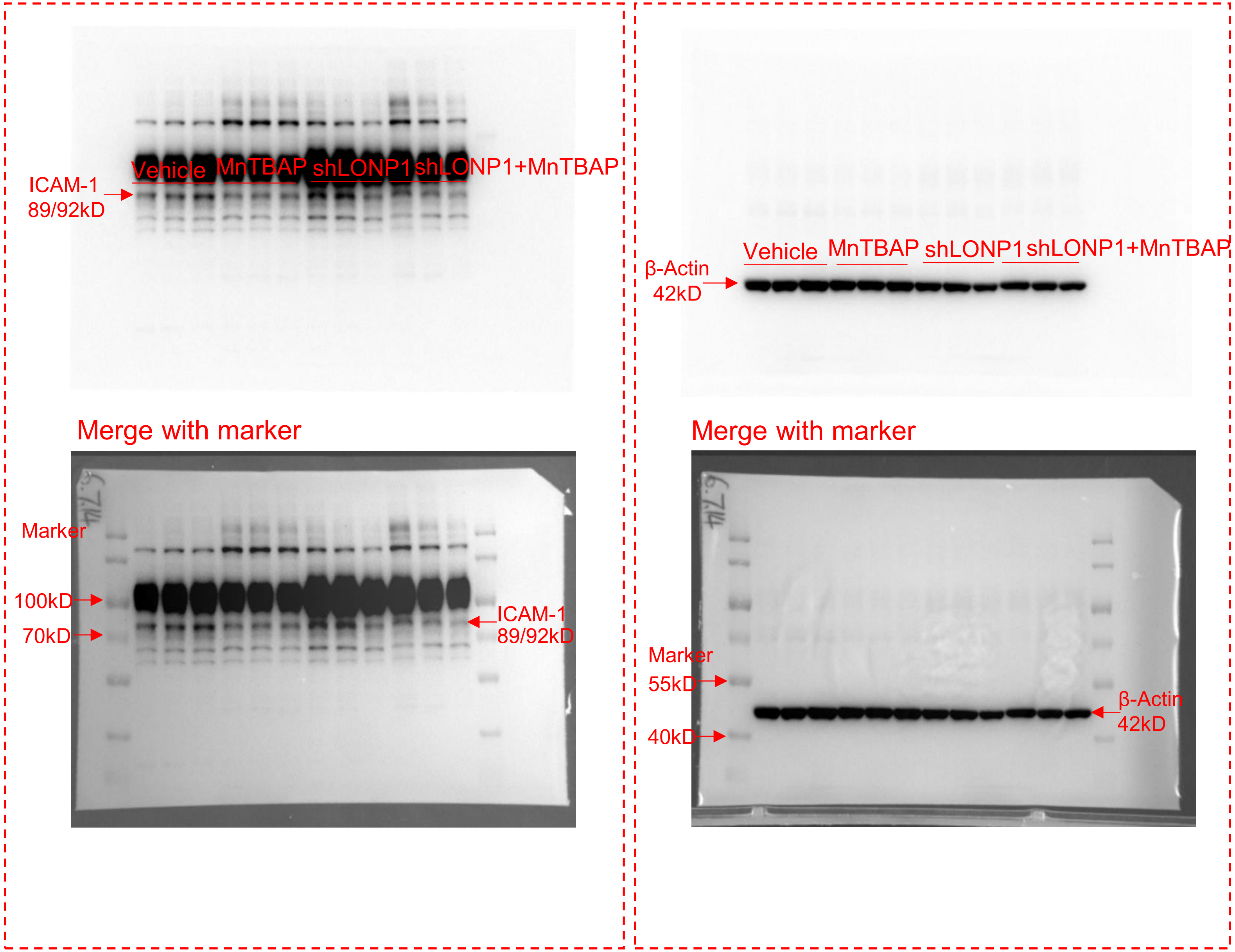

Fig5P MAECs transfected with shLONP1 plasmid and treated with MnTBAP

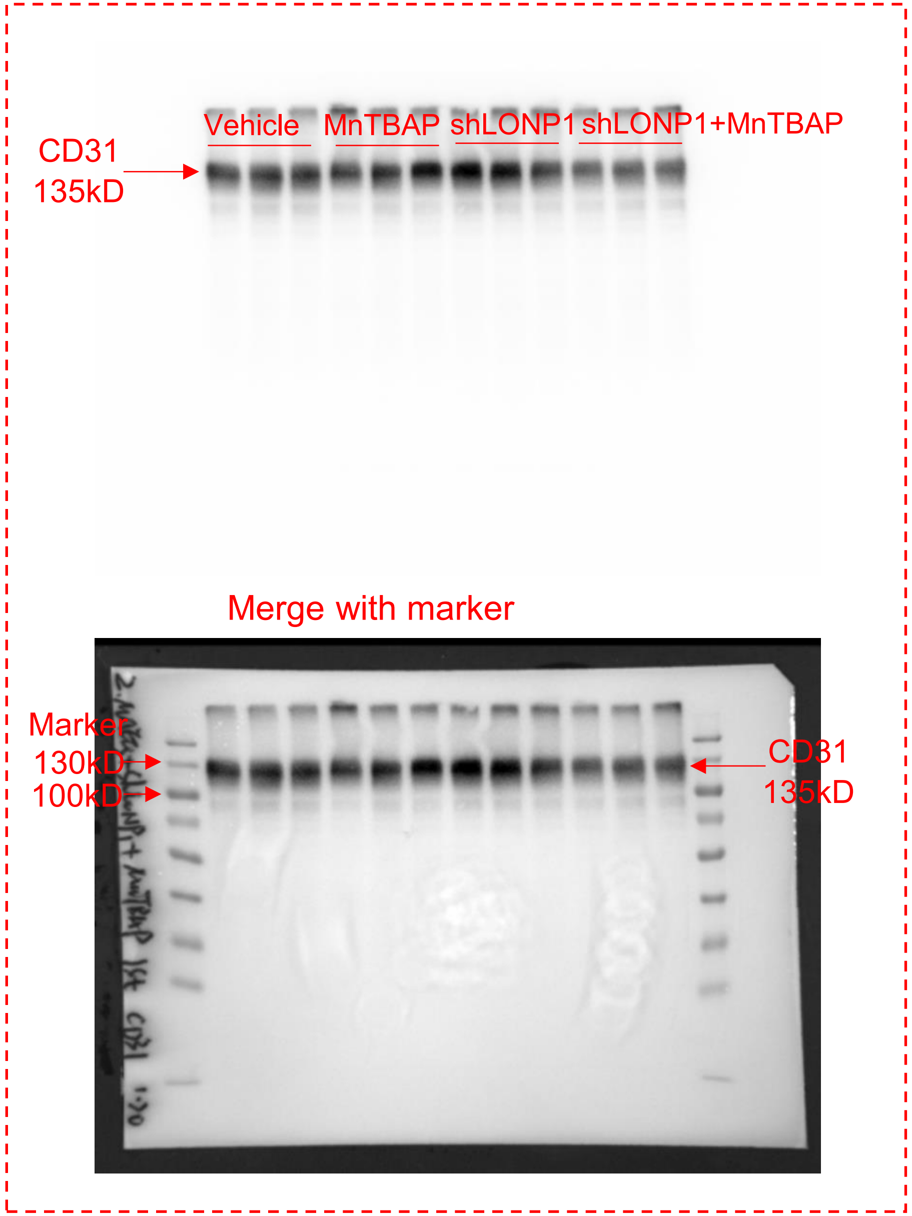

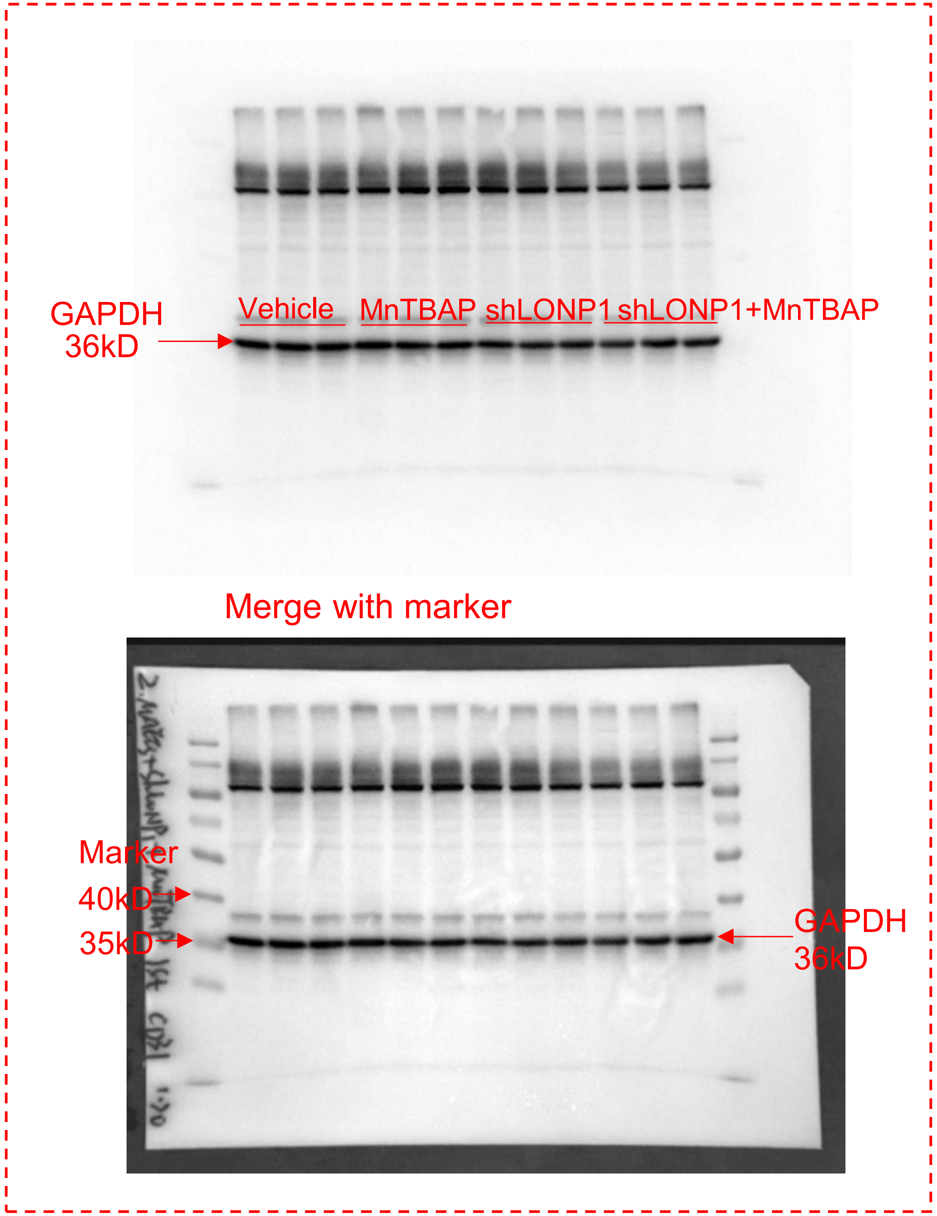

Fig5P MAECs transfected with shLONP1 plasmid and treated with MnTBAP

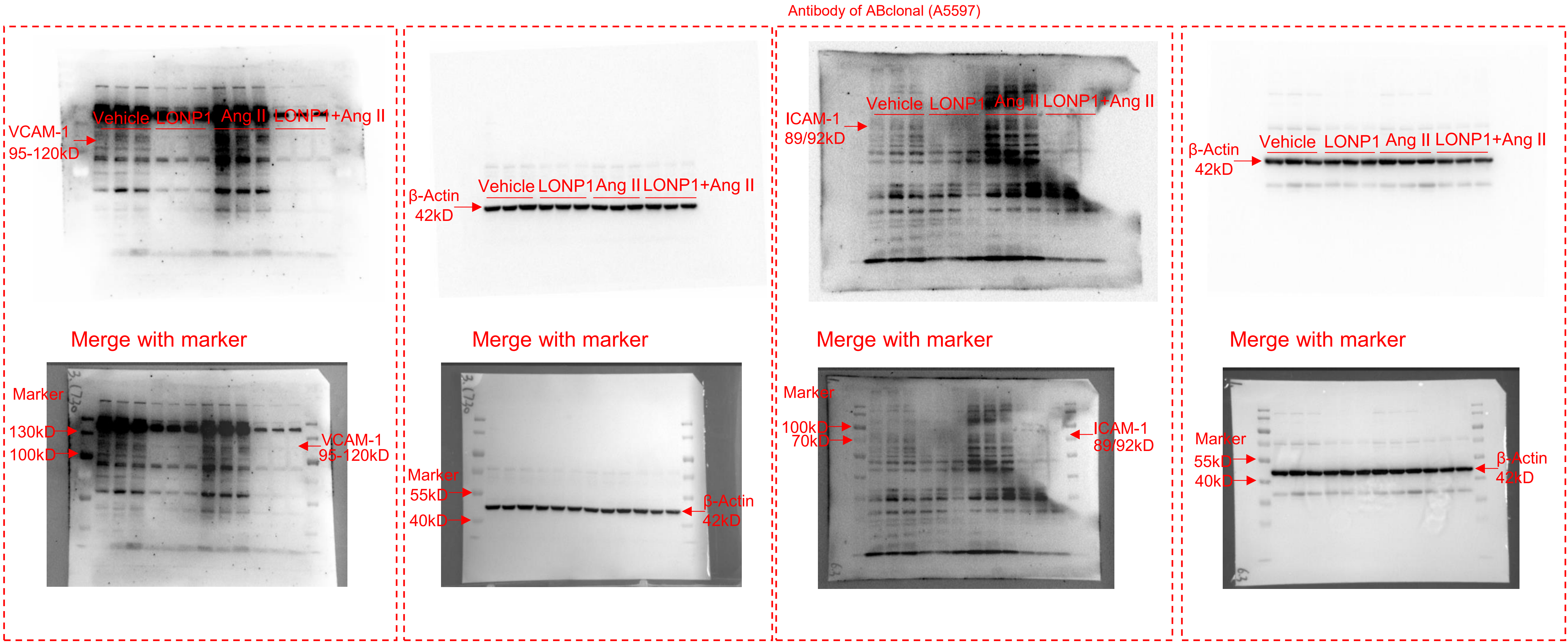

Fig5T Immortalized HAECs co-transfected with SOD2 overexpression plasmid and shLONP1 plasmid

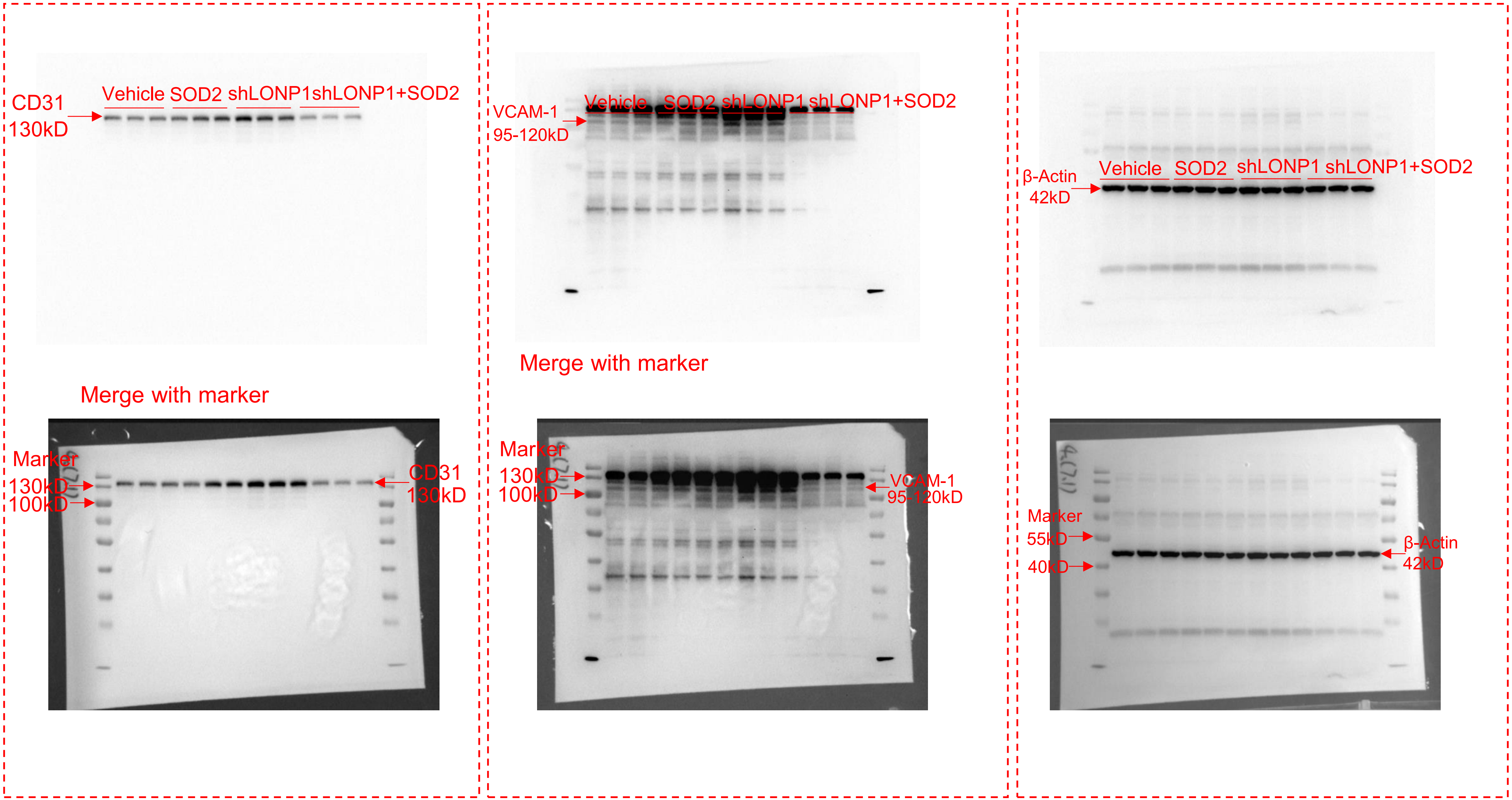

Fig5T Immortalized HAECs co-transfected with SOD2 overexpression plasmid and shLONP1 plasmid

Antibody of ABclonal (A5597)

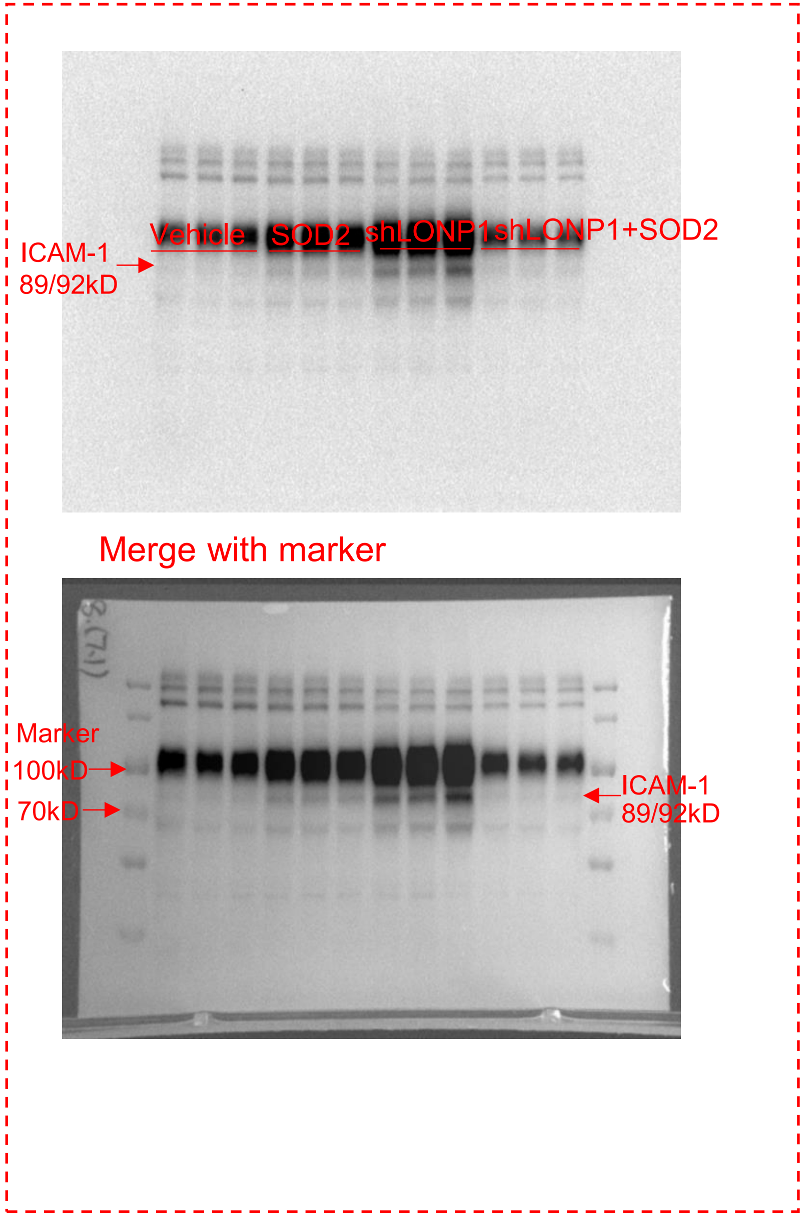

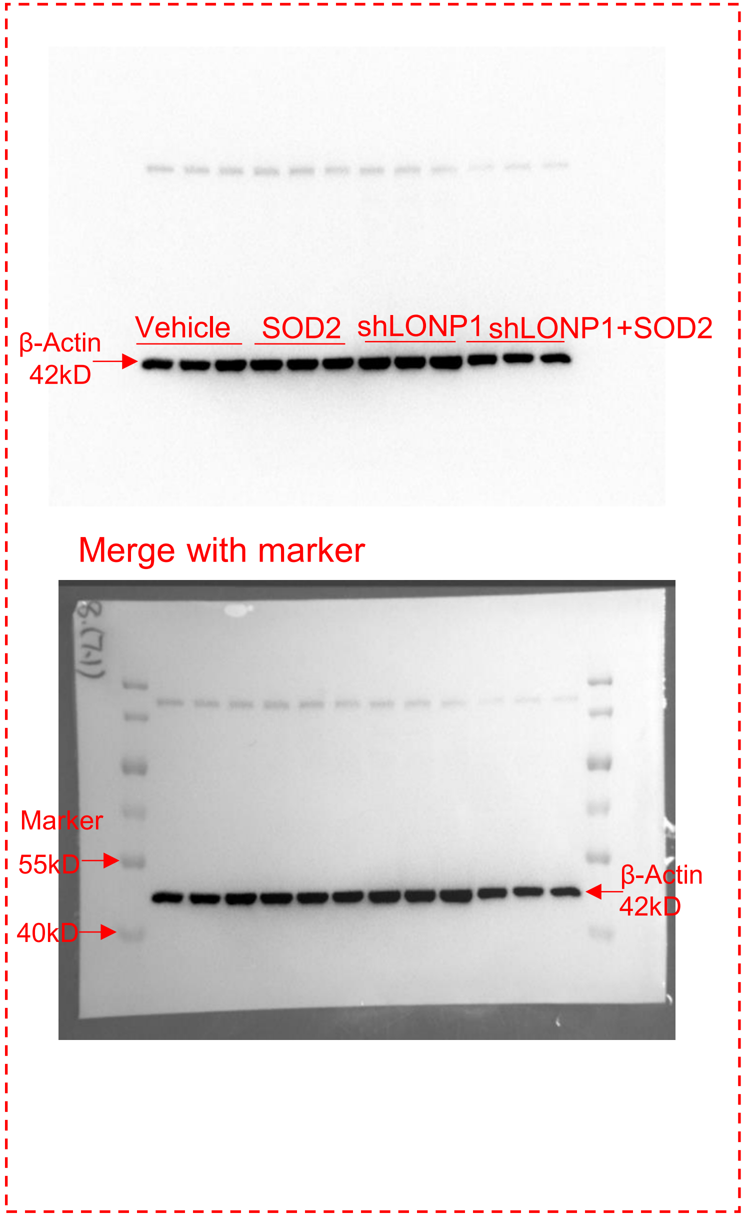

Fig5V MAECs co-transfected with SOD2 overexpression plasmid and shLONP1 plasmid

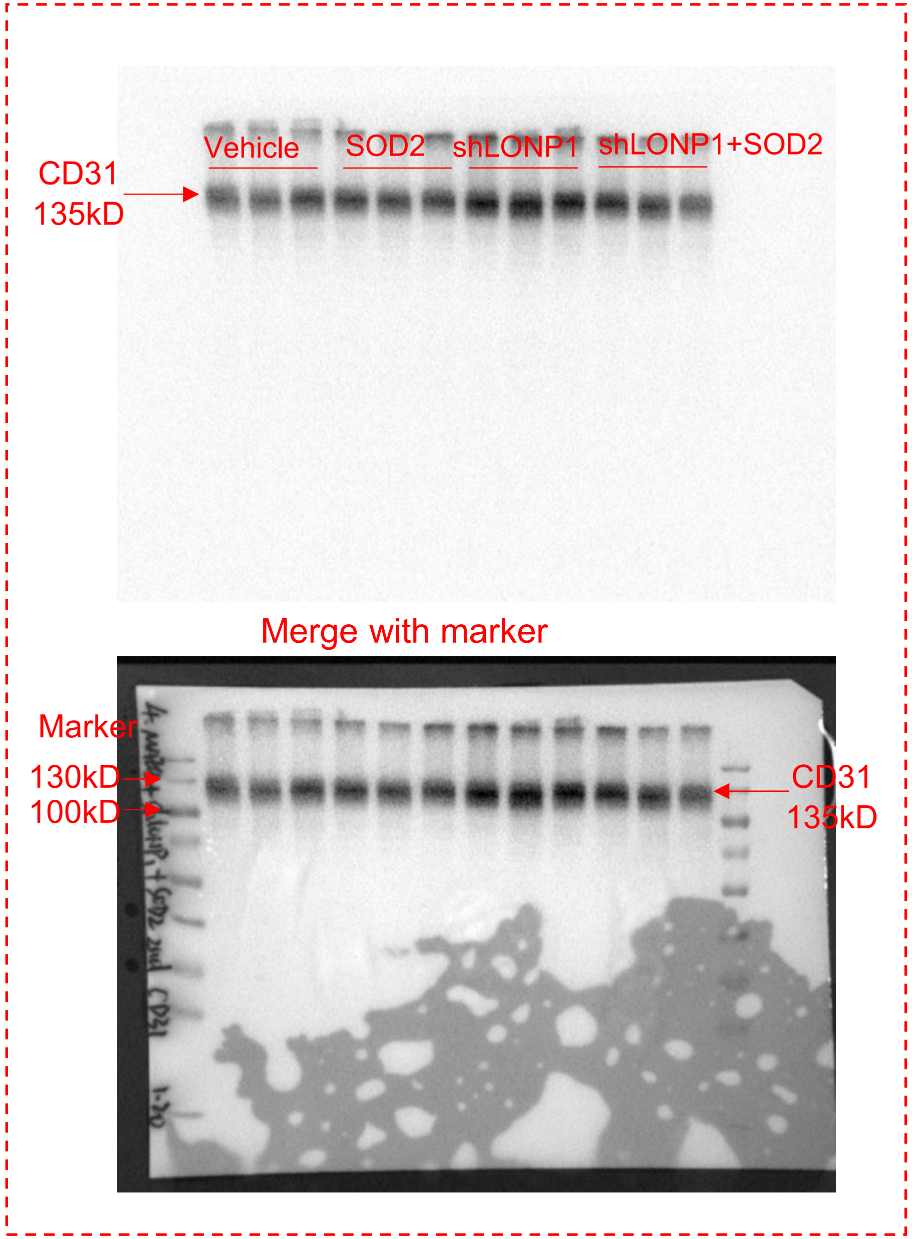

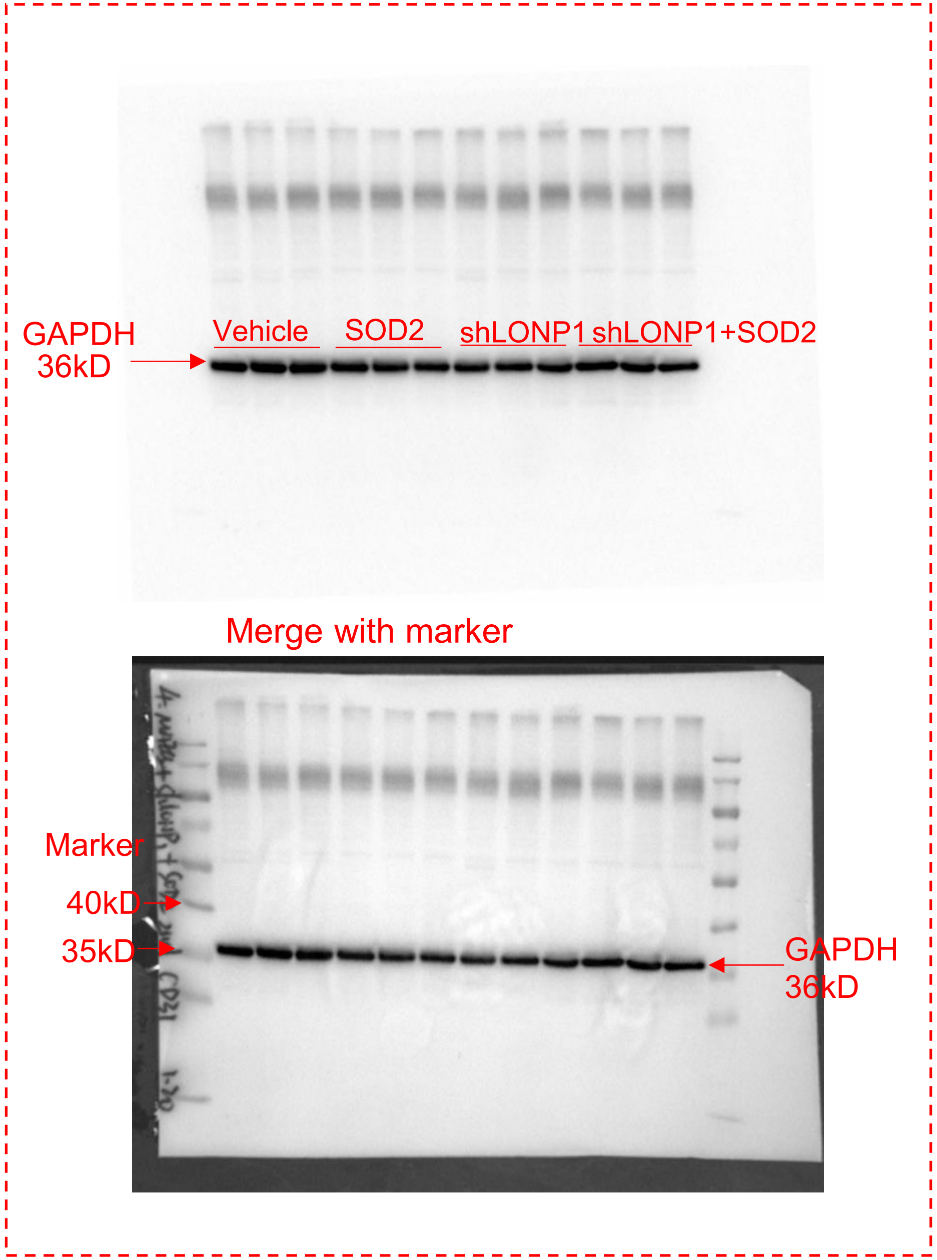

Fig5V MAECs co-transfected with SOD2 overexpression plasmid and shLONP1 plasmid

Antibody of ABclonal (A26412PM)

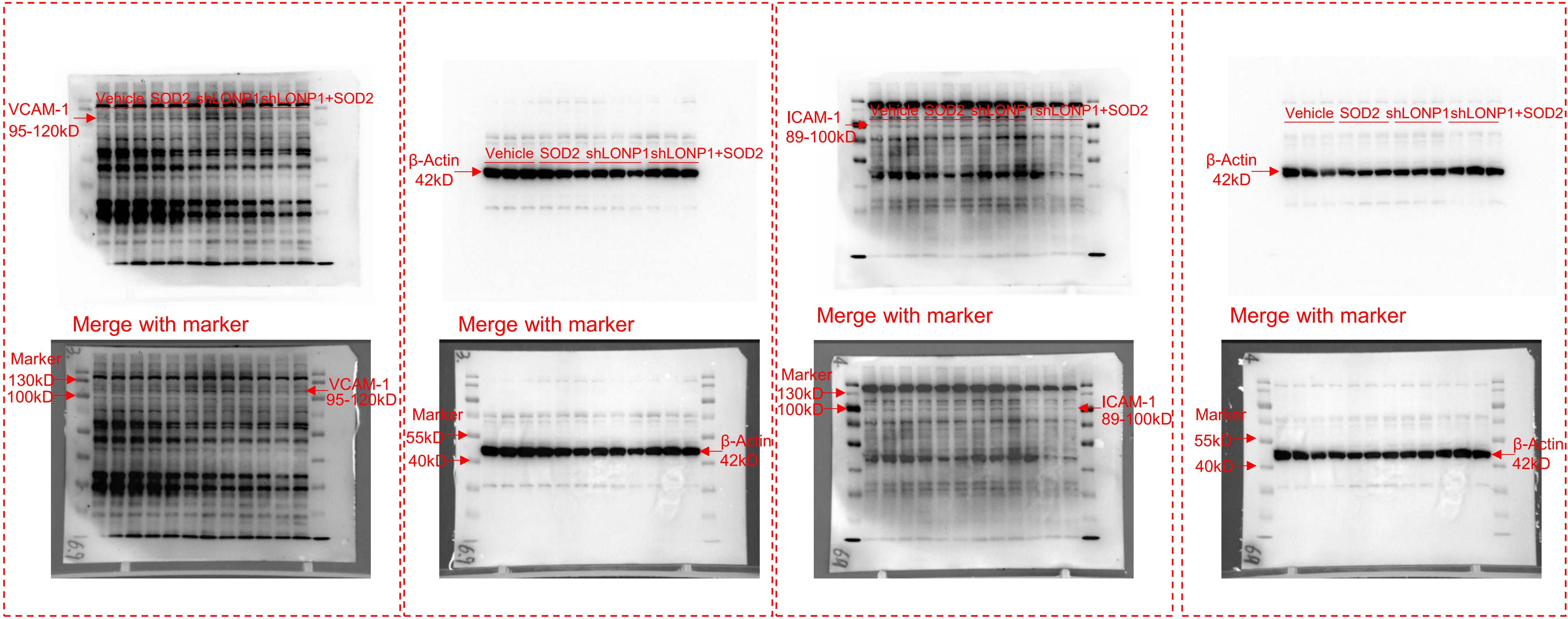

Fig6A MCs treated with the culture supernatant of MAECs from each group-Represented images

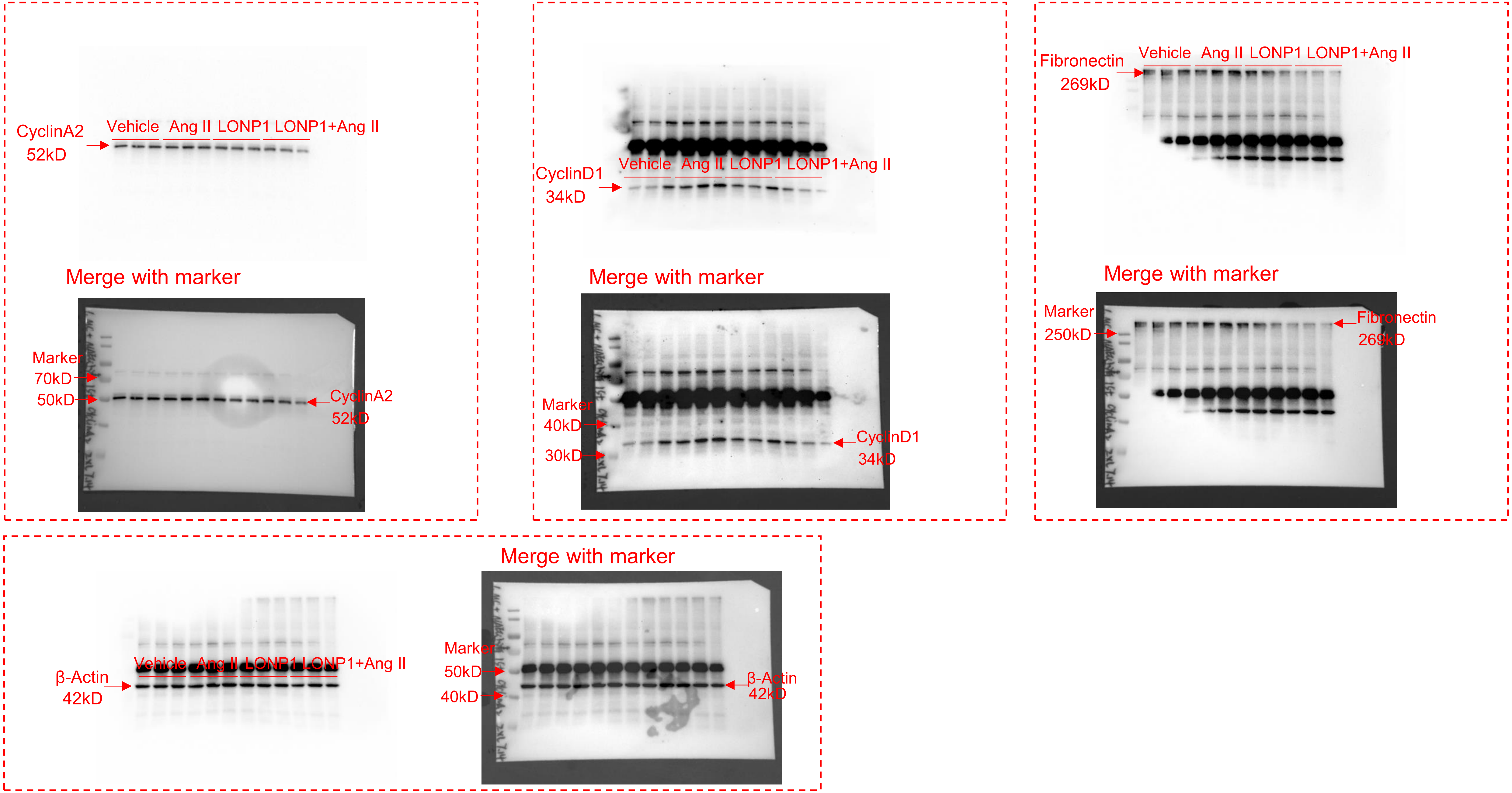

Fig6A MCs treated with the culture supernatant of MAECs from each group-Repeated images

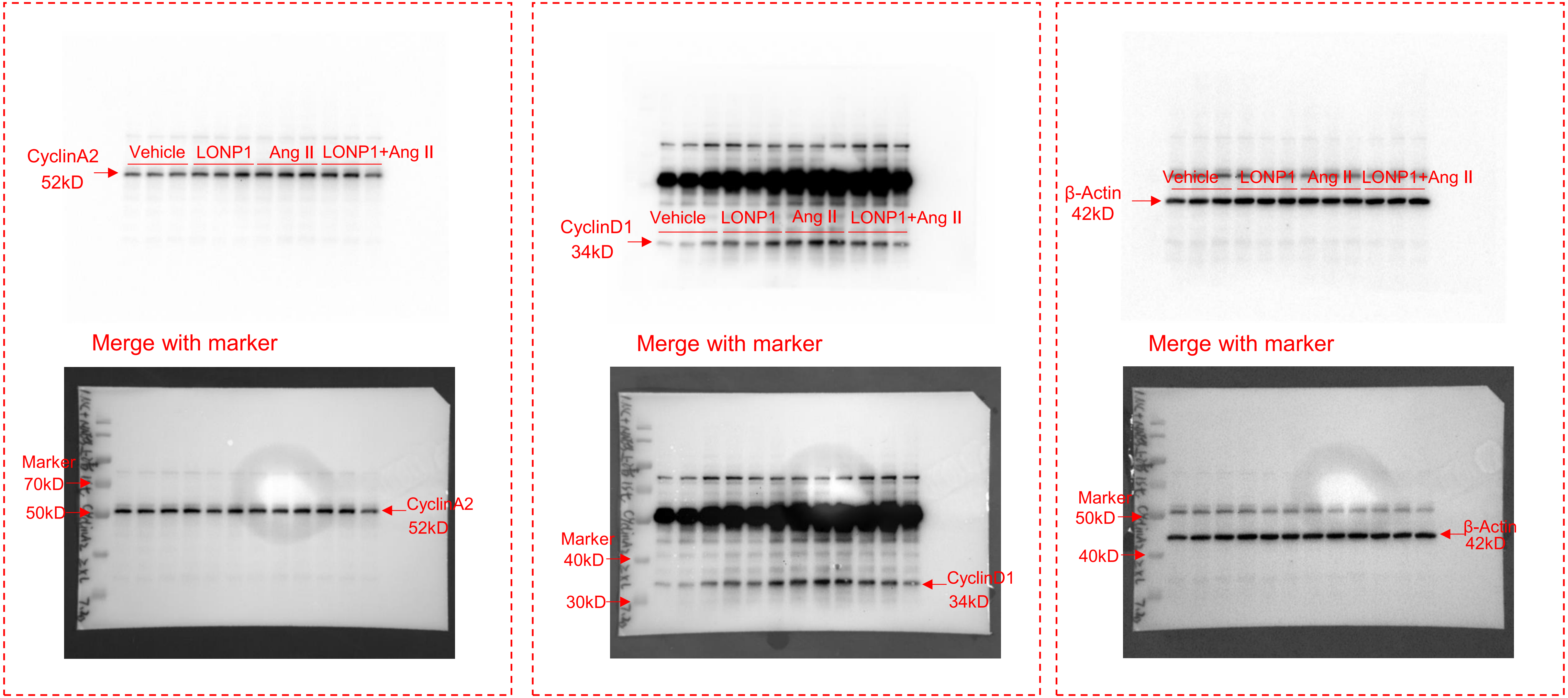

Fig6E MCs treated with the culture supernatant of MAECs from each group

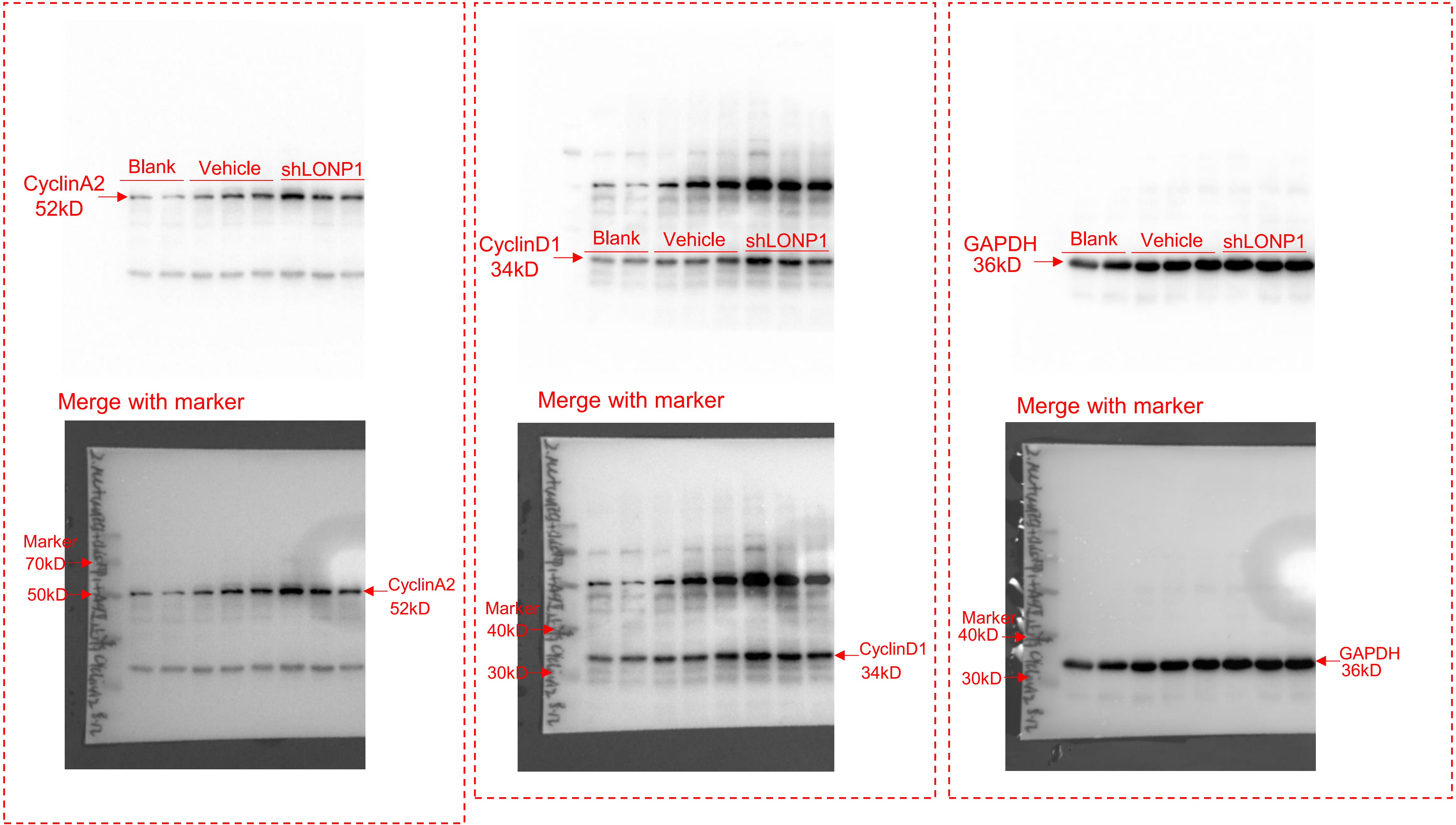

Fig6I MCs treated with the culture supernatant of MAECs from each group

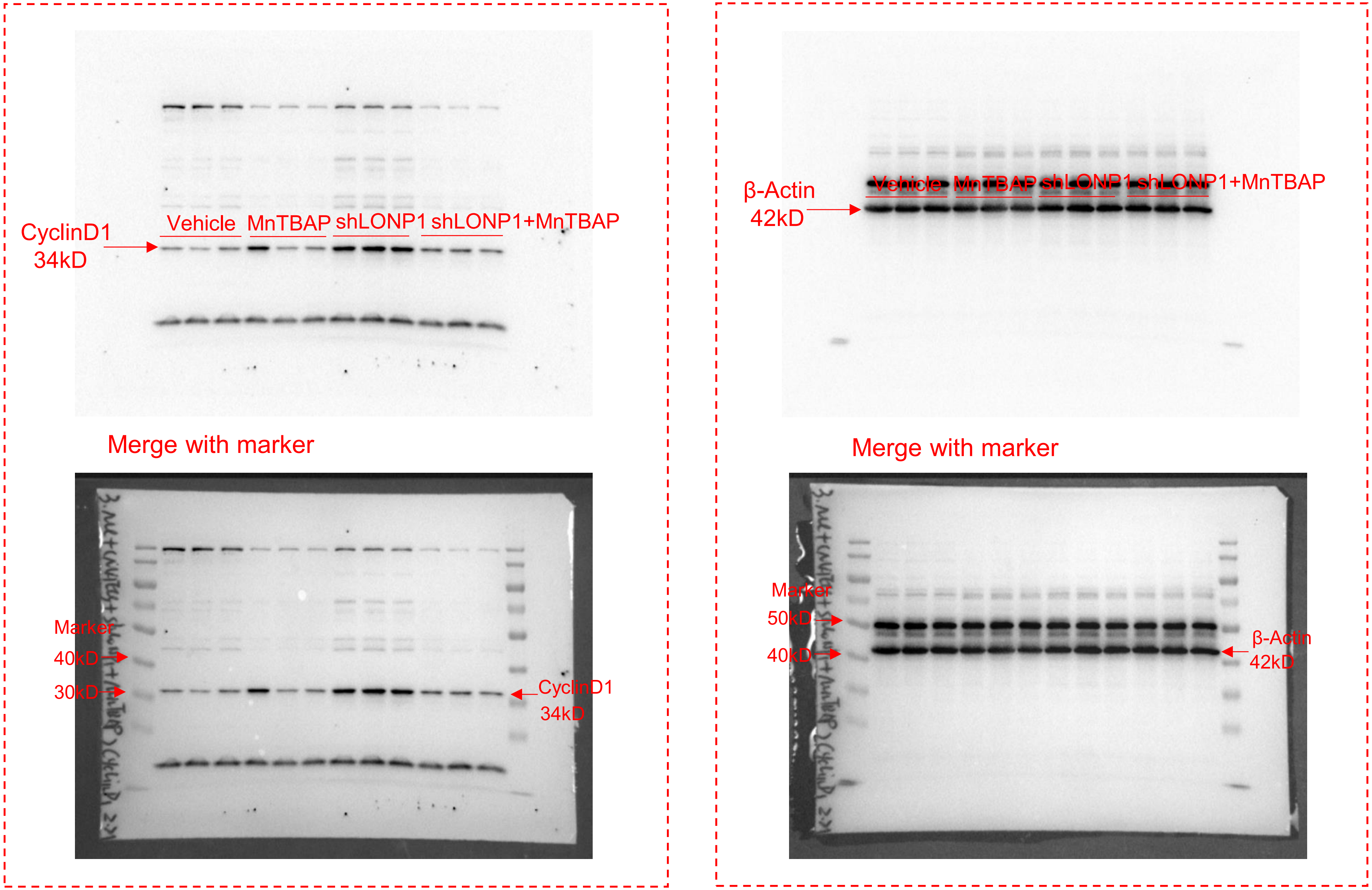

Fig6K MCs treated with the culture supernatant of MAECs from each group

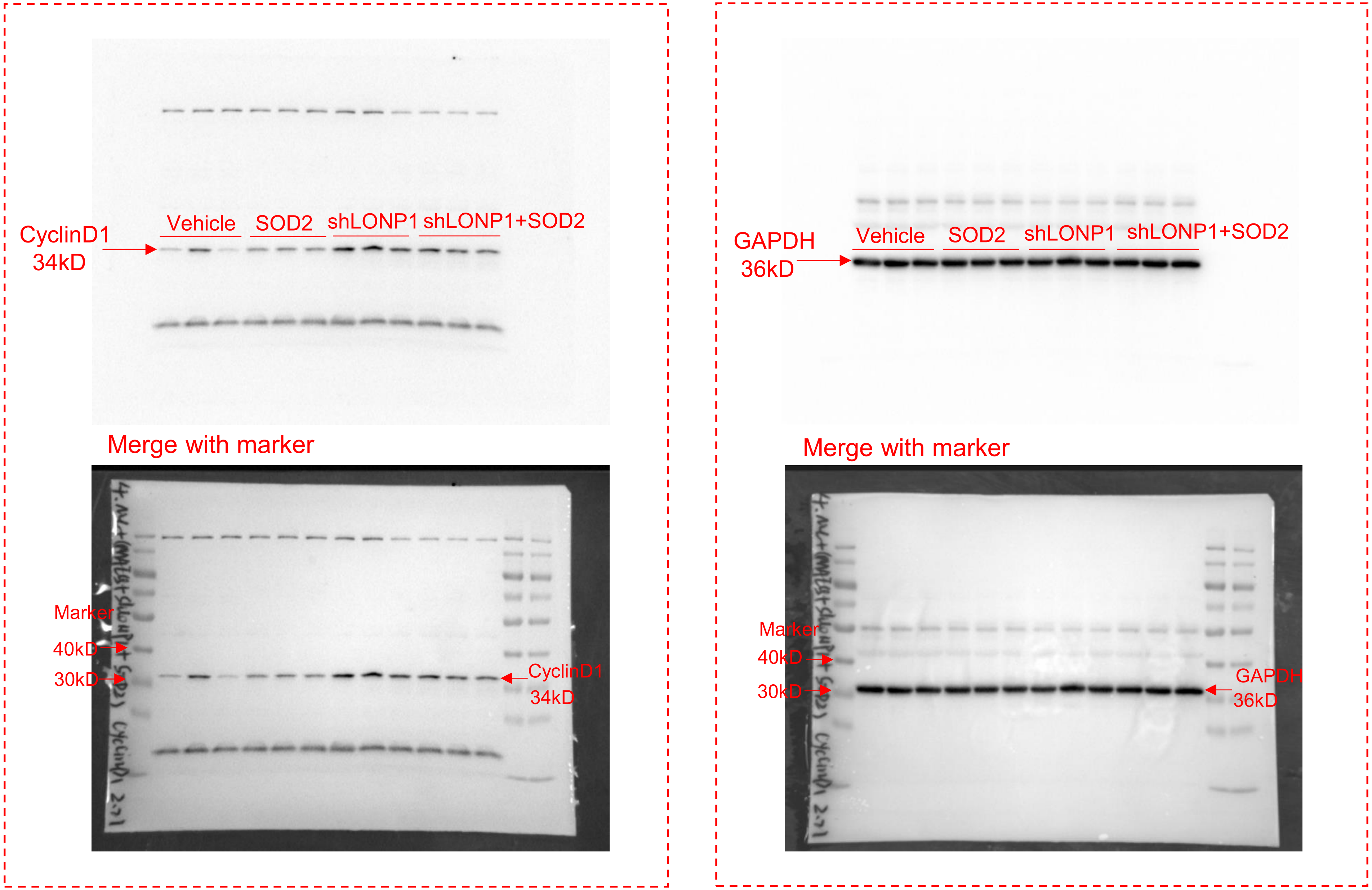

Fig7B MPCs treated with the culture supernatant of MAECs from each group

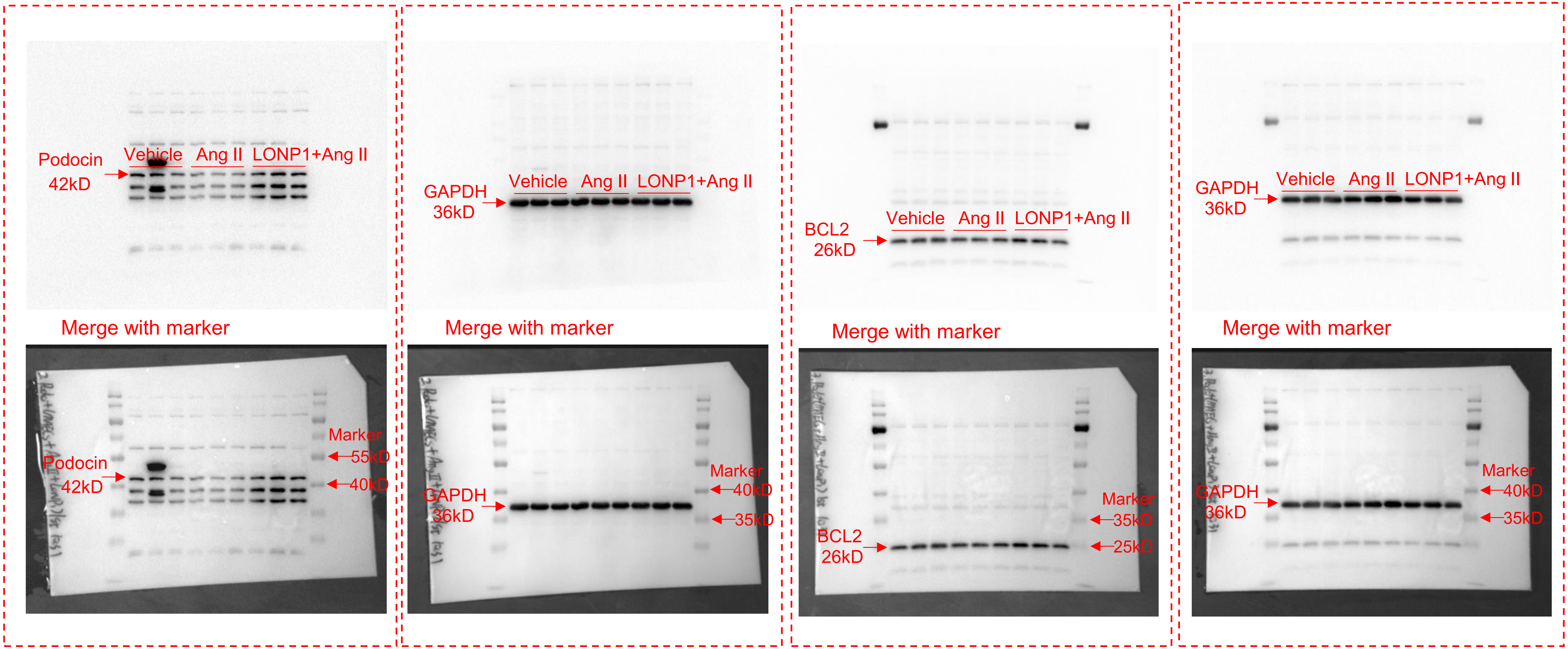

Supplementary Fig3C

glomerular endothelial cells and the rest cells of LONP1 hetero cKO mouse

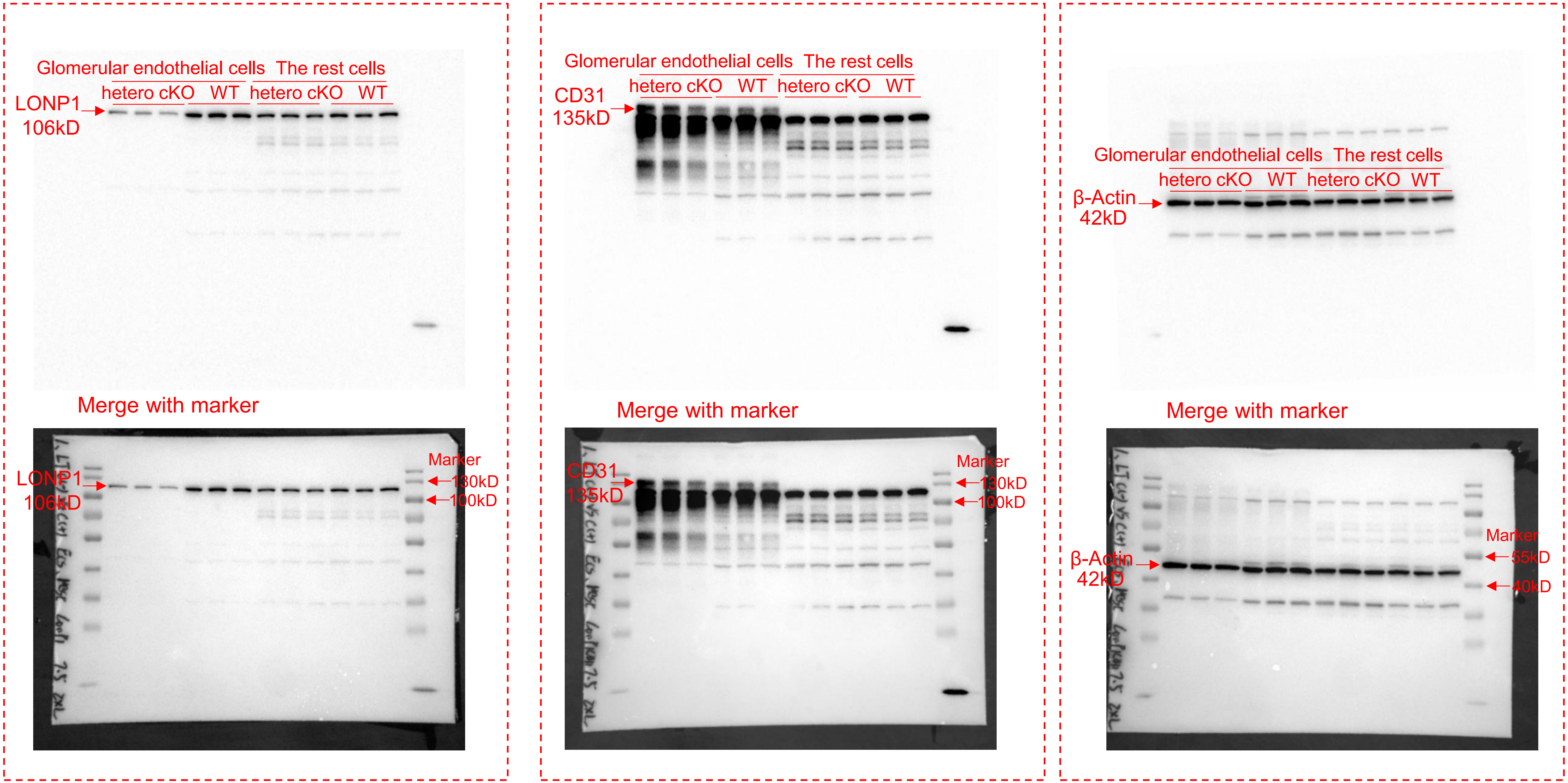

Supplement: Multimedia component 3 [file mmc3.pdf]
